# CLK-dependent exon recognition and conjoined gene formation revealed with a novel small molecule inhibitor

**DOI:** 10.1038/s41467-016-0008-7

**Published:** 2017-02-23

**Authors:** Tyler Funnell, Shinya Tasaki, Arusha Oloumi, Shinsuke Araki, Esther Kong, Damian Yap, Yusuke Nakayama, Christopher S. Hughes, S.-W. Grace Cheng, Hirokazu Tozaki, Misa Iwatani, Satoshi Sasaki, Tomohiro Ohashi, Tohru Miyazaki, Nao Morishita, Daisuke Morishita, Mari Ogasawara-Shimizu, Momoko Ohori, Shoichi Nakao, Masatoshi Karashima, Masaya Sano, Aiko Murai, Toshiyuki Nomura, Noriko Uchiyama, Tomohiro Kawamoto, Ryujiro Hara, Osamu Nakanishi, Karey Shumansky, Jamie Rosner, Adrian Wan, Steven McKinney, Gregg B. Morin, Atsushi Nakanishi, Sohrab Shah, Hiroyoshi Toyoshiba, Samuel Aparicio

**Affiliations:** 1 Department of Molecular Oncology, BC Cancer Agency, 675 West 10th Avenue, Vancouver, British Columbia Canada V5Z 1L3; 2 Department of Pathology and Laboratory Medicine, University of British Columbia, Vancouver, British Columbia Canada V6T 2B5; 3 Pharmaceutical Research Division, Takeda Pharmaceutical Company Limited, 26-1, Muraoka-Higashi 2-chome, Fujisawa, Kanagawa 251-8555 Japan; 4 Michael Smith Genome Sciences Centre, BC Cancer Agency, 675 West 10th Avenue, Vancouver, British Columbia Canada V5Z 1L3; 5 Department of Medical Genetics, University of British Columbia, Vancouver, British Columbia Canada V6H 3N1; 6 Janssen Pharmaceuticals, Toronto, Ontario Canada; 7 Fujirebio Inc., Tokyo, Japan; 8 Department of Innovative Drug Discovery and Development, Japan Agency for Medical Research and Development, Osaka, Japan; 9 Advanced Research Computing, University of British Columbia, Vancouver, British Columbia Canada

## Abstract

CDC-like kinase phosphorylation of serine/arginine-rich proteins is central to RNA splicing reactions. Yet, the genomic network of CDC-like kinase-dependent RNA processing events remains poorly defined. Here, we explore the connectivity of genomic CDC-like kinase splicing functions by applying graduated, short-exposure, pharmacological CDC-like kinase inhibition using a novel small molecule (T3) with very high potency, selectivity, and cell-based stability. Using RNA-Seq, we define CDC-like kinase-responsive alternative splicing events, the large majority of which monotonically increase or decrease with increasing CDC-like kinase inhibition. We show that distinct RNA-binding motifs are associated with T3 response in skipped exons. Unexpectedly, we observe dose-dependent conjoined gene transcription, which is associated with motif enrichment in the last and second exons of upstream and downstream partners, respectively. siRNA knockdown of CLK2-associated genes significantly increases conjoined gene formation. Collectively, our results reveal an unexpected role for CDC-like kinase in conjoined gene formation, via regulation of 3′-end processing and associated splicing factors.

## Abstract (web summary)

The phosphorylation of serine/arginine-rich proteins by CDC-like kinase is a central regulatory mechanism for RNA splicing reactions. Here, the authors synthesize a novel small molecule CLK inhibitor and map CLK-responsive alternative splicing events and discover an effect on conjoined gene transcription.

## Introduction

Alternative splicing (AS) is a central mechanism for physiological regulation of protein diversity^1–3^. AS of pre-messenger RNA (mRNA) occurs through modulation of *trans*-acting splice accessory factors, RNA-binding proteins (RBPs), and their *cis*-acting binding sites (splice signals such as the 3′ and 5′ splice sites, branchpoint, and splicing regulatory elements)^4^. AS has been implicated in a wide range of diseases, including cancer^5–7^ and pre-malignant states such as myelodysplasia^8–11^ through mutation of SF3B proteins. Recently, CLK2 has been suggested as an oncogenic kinase in breast cancer^12^ and CLK1-regulated AS is deregulated in kidney cancers^13^.

The selection of alternatively spliced exonic or intronic pre-mRNA sequences are typically regulated by serine/arginine-rich (SR) proteins, which are RBPs that are modulated through phosphorylation by several protein kinase families, including the CDC-like kinases (CLKs) and the SR protein kinases^14^. The phosphorylation-dependent signaling is one important step for the regulation of cellular localization and activity of these SR proteins during splicing^15,16^. Several small molecule tool compound inhibitors of CLKs have been shown to affect specific exon splicing events^17–19^; however, these molecules are complicated by activity at other targets (e.g., TG003, which does not inhibit CLK3 and shows cross-reactivity with CK1d and CK13, DYRK1B, YSK4, and PIM kinase isoforms^20^). The most potent and CLK family-specific inhibitor described to date (KH-CB19)^19^ has recently been used to identify a novel global class of detained introns (DI) in splicing reactions^21^, but still lacks sufficient potency and selectivity against CLK members for a full exploration of CLK activity. Here, we describe a highly selective, stable, novel CLK small molecule inhibitor with high specificity to CLK1–3 protein isoforms. This compound, herein referred to as T3, contains a modified kinase hinge binder region, compared to the most potent and selective previously described CLK facility tool compound KH-CB19^19^, and has a more stable interaction with the target proteins, exhibiting approximately 2 log order improved potency and selectivity for CLK isoforms. T3 exhibits an overlapping, but greater effect on transcriptome splicing compared to KH-CB19. We show that CLK-dependent reactions occur in distinct genomic sequence contexts with sequence-encoded features that are relatively conserved between different epithelial cell types. We also reveal an unexpected role for CLK proteins in conjoined gene (CG) transcription, implicating CLK in 3′-end processing of transcripts.

## Results

### Synthesis and characterization of a novel CLK inhibitor

**Fig. 1:**
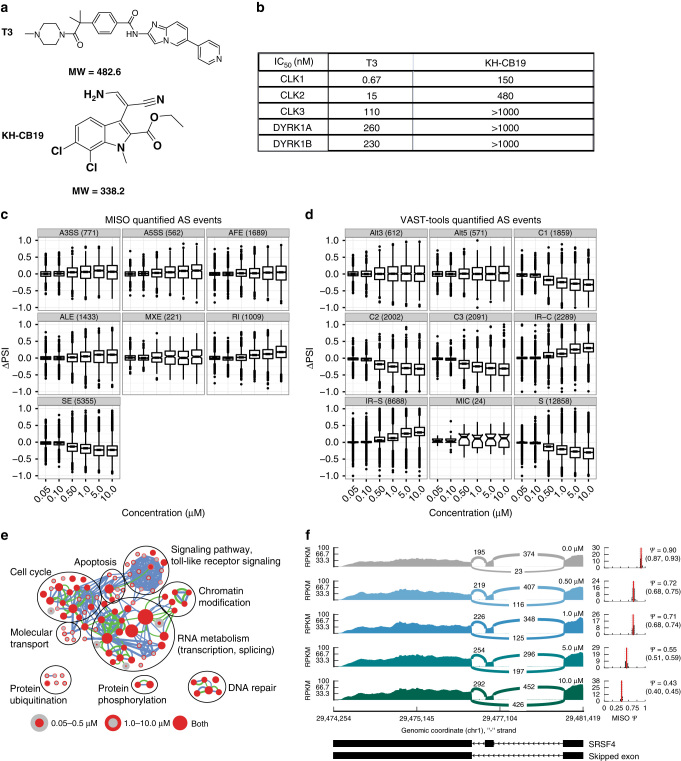
**AS responses to T3 analyzed by event type and biological enrichment.** **a** Chemical structure of “T3” and KH-CB19. **b** Inhibition spectrum of the compounds for CLK1, CLK2, CLK3, DYRK1A, and DYRK1B (1 mM of ATP was used for CLK1, CLK2, CLK3, DYRK1A, and DYRK1B kinase). **c** MISO AS event type ∆PSI distributions across CLK inhibitor concentration for the HCT116 unstranded RNA-Seq data set. Horizontal axis categories are concentrations of T3. SE, RI, A5SS, and A3SS event classes are as described in the main text; AFE: alternative first exon, ALE: alternative last exon, MXE: mutually exclusive exon. Event counts in parentheses. **d** VAST-tools AS event type ∆PSI distributions across CLK inhibitor concentration for the HCT116 unstranded RNA-Seq data set. Horizontal axis categories are concentrations of T3. VAST-tools event types are: S, C1, C2, C3: SE with increasing complexity, MIC: skipped microexons, IR-S: RI not overlapping another annotated event, IR-C: RI overlapping another annotated event, Alt3: alternative acceptor site, Alt5: alternative donor site. Event counts in parentheses. **e** BP enrichment map for differentially spliced genes in the HCT116 unstranded RNA-Seq data set. Each node represents a GO BP gene set. Node cores are colored *red* when that gene set is enriched among genes differentially spliced in the 0.05–0.5 µM samples, and the outer ring is colored *red* when that gene set is enriched in the 1.0–10.0 µM samples. Nodes present in both are *solid* colored. Edge thickness indicates the level of overlap between two gene sets, considering the set of differentially spliced genes in the 0.05–0.5 µM (*green edges*) or 1.0–10.0 µM (*blue edges*) samples. **f** Sashimi style plot^62^ of exon skipping events associated with the SRSF4 gene. RPKM values are displayed as 0–100 along the *vertical axis* and genome coordinates (bp) along the *horizontal axis*. Each strip represents increasing T3 concentration, value displayed on the right. The number of events represented by each splice event are inserted in the center of the curved lines. Right of the main plot, MISO PSI posterior distribution

We developed a novel CLK family small molecule inhibitor with low nM potency, selectivity over other kinases, and favorable cell-based in vitro characteristics, 4-(2-methyl-1-(4-methylpiperazin-1-yl)-1-oxopropan-2-yl)-N-(6-(pyridin-4-yl)imidazo[1,2-a]pyridin-2-yl)benzamide, referred to herein as “T3” (Fig. 1a), by chemical modifications of the structure of previously identified tool compounds^22^ from an extensive high throughput chemical library screen against CLK2 protein (Supplementary Tables 1, 2). The structure–activity relationship (Supplementary Fig. 1 and Supplementary Notes), structure (Supplementary Fig. 2) and synthetic route leading to T3, as well as the kinase inhibition profile (Supplementary Fig. 3a) and stability in culture medium (Supplementary Table 3) are described in the “Methods” and Supplementary Information.

T3 exhibited significant inhibitory activity against CLK1, CLK2, and CLK3 protein kinases with IC_50_ values of 0.67, 15, and 110 nM, respectively (Fig. 1b). This was mirrored in cell-based in vitro conditions in a long-term (>24 h) cytotoxicity assay, when compared with KH-CB19 head to head in the same assay (Supplementary Fig. 3b). Short-term exposure of T3 (>6 h) does not result in cellular cytoxicity or apoptosis even at high concentrations of compound (Supplementary Fig. 4). While the spectrum activity of CLK4 was not directly measured in this assay, T3 is expected to exhibit some inhibition of CLK4 based on amino acid sequence homology with CLK1 (69% identity). The selectivity of T3 over other CMGC sub-family members was further confirmed using other dual specificity kinases such as DYRK1A and DYRK1B as substrates in the kinase enzymatic assay (200–300 times weaker inhibition compared with CLK, Fig. 1b). The inhibition spectrum of this compound was also measured across a panel of 71 kinases involved in multiple signaling pathways critical for proliferation and cell homeostasis, showing no significant inhibition beyond the CMGC family (Supplementary Data 1, Supplementary Fig. 3a). Intracellular on-target activity was confirmed by several routes. First, the strong cell-based in vitro on-target effect of CLK inhibition (CLKi) on phosphorylation of SRSF1, SRSF4, and SRSF6, canonical targets of CLK in exon recognition (Supplementary Fig. 5a), compared with KH-CB19 and consistent with the in vitro differences in potency of these two different chemical scaffolds. Second, RNA-Seq (see below) revealed overlap in a specific novel class of DI observed with KH-CB19. Third, global RNA-Seq of combinations of *CLK1/2/3/4* RNA interference (RNAi) experiments (below and in Supplementary Information) revealed 55% of RNAi alternative splice events overlapped with T3-induced AS. Fourth, the major observed activity on splicing determined by RNA-Seq was exon skipping and retained introns (RI), in a dose-dependent manner, consistent with the known function of CLK in splicing^17,18,21^.

### T3 induces dose-dependent reduction in exon recognition

We first determined the global effects of T3-mediated CLKi on transcript AS by incubating transformed malignant colorectal cancer cells (HCT116) and non-transformed mammary epithelial cells (184 hTERT)^23^ in increasing concentrations of T3 inhibitor during short-duration exposure (6 h, chosen to avoid any cytotoxicity or significant cell cycle arrest) experiments, assaying the splicing consequences using RNA-Seq (39 Illumina libraries and 3 Pacbio long-read libraries, Supplementary Tables 4, 5). Quantification of AS events was undertaken with MISO^24^ and VAST-tools^25^. Details of the technical, biological, and methodological cross-validation and workflow are in the Supplementary Information (Supplementary Figs. 6, 7, Supplementary Data 2, 3, 4, 5, Supplementary Notes). We observed no nonsense mediated decay (NMD) inhibition at the concentrations of T3 used in these experiments (Supplementary Fig. 8), indicating that splice isoforms observed are unlikely to result from the accumulation of transient NMD isoforms.

Considering all event types together, the sum of ∆AS (AS difference between control and drug treatment conditions) events showed a dose-dependent increase (Supplementary Fig. 9, Supplementary Data 6, 7), with the greatest rate of increase in ∆AS events at 0.50 µM (4.1-fold increase vs. 0.10 µM in HCT116 unstranded RNA-Seq), consistent with the potency profile of the T3 compound. We also compared T3 with KH-CB19 in targeted PCR splicing assays, and noted that in these comparisons T3 shows distinct dose-dependent AS effects at T3 concentrations more than 1 log order below that of KH-CB19 (Supplementary Fig. 10), consistent with the in vitro potency measurements. We observed a large number of overlapping T3-dependent AS events between the two different cell types (~75% of hTERT AS events were also detected in HCT116 cells, Supplementary Fig. 11a) and also T3 concentration-dependent ∆AS dynamics (Supplementary Fig. 12, HCT116-stranded, hTERT-stranded). This shows that a large common set of AS events are mediated in different cell types by CLK activity.

To examine transcript structure modulation, we calculated ∆PSI (percent spliced in) value distributions at each T3 concentration for each event type, which revealed AS dynamics (Fig. 1c, d, Supplementary Fig. 12a, b). Skipped exons (SE) are the most prevalent ∆AS event type (52% of ∆AS events across all T3-treated libraries) and also exhibit the strongest PSI dynamics, decreasing with increasing drug concentration, indicating that these exons skip more often with CLKi (e.g., *SRSF4*, Fig. 1f). The greatest rate of PSI decrease occurs at the 0.50 µM concentration (6.5-fold decrease in median vs. 0.10 µM), consistent with the in vitro and cellular IC_50_ of the T3 compound. In a reciprocal manner, RI events increase in PSI with increasing CLK inhibitor concentration (4.5-fold increase in median at 0.50 vs. 0.10 µM), demonstrating a tendency for introns to be retained with increasing CLKi. In contrast to SE and RI events, alternative 3′ and 5′ splice sites (A3SS and A5SS) events both see a more gradual increase in PSI (i.e., increased exon extension) and the overall prevalence of these event types is much lower than other classes (11% of ∆AS events in T3-treated libraries). Very similar patterns (Fig. 1d, Supplementary Fig. 12c, d, Supplementary Data 8, 9) were found with an alternative annotation-quantification method, VAST-tools, which also detects splicing in micro-exons^25^, although the latter do not exhibit differences with CLKi.

DI are a novel class of delayed, post-transcriptionally spliced introns^21^, recently shown to exhibit CLK dependence via KH-CB19-mediated CLKi. We noted that 702 MISO RI events overlap with reported human DIs, and of these 150 (21%), 86 (12%), and 94 (13%) were differentially spliced in the HCT116 unstranded, stranded, and 184hTERT RNA-Seq data sets. We quantified PSI values for all 13,308 DI events and found 1009 (8%), 508 (4%), and 484 (4%) differentially spliced in the HCT116 unstranded, stranded, and 184hTERT data sets (Supplementary Data 10, 11). DI PSI values generally increased with T3 treatment (Supplementary Fig. 13), supporting the notion that a subset of DIs are sensitive to CLKi^21^ with T3.

Despite limited depletion of *CLK* transcripts achievable with RNAi, particularly in combination knockdowns (Supplementary Table 6), >55% of events from siRNA RNA-Seq overlapped with T3-mediated CLKi (Supplementary Fig. 11b, Supplementary Data 12). Biological processes (BP) enriched (FDR < 0.05) among genes differentially spliced in both T3-treated and *CLK* siRNA transfected cells included “gene expression”, “mitotic cell cycle”, “chromatin modification”, and “nuclear mRNA splicing, via spliceosome” (Supplementary Data 13). Taken together, the observed dose-dependent AS patterns to T3-mediated CLKi and overlap with weaker inhibitors and RNAi are consistent with the known role of CLK proteins in exon recognition.

Gene ontology (GO) BP enrichment–Cytoscape enrichment maps^26^ (FDR < 0.05) contrasting low concentration T3 (0.05–0.5 µM) with high concentration T3 (1.0–10.0 µM) (Fig. 1e, Supplementary Fig. 14a, b, Supplementary Data 14) revealed splicing factors to be affected at low and high concentrations. This is consistent with the notion that splicing proteins are themselves strongly auto-regulated^27^. Interestingly, while phosphorylation of CLK-targeted SR proteins is decreased by T3, CLK proteins increase slightly (Supplementary Figs. 5b, 15). Cell cycle regulators were also affected at low and high concentrations consistent with the observation that long-duration (24 h) T3 exposure results in mild cell cycle arrest at the G2/M boundary (Supplementary Fig. 16, Supplementary Fig. 4). In contrast, genes involved in toll-like receptor signaling were statistically over-represented only in the higher concentration 1.0–10.0 µM contrasts (Fig. 1e), suggesting the latter to be a secondary consequence of CLKi or potentially off-target effects at high doses.

### Distinct RBP motifs are associated with ∆AS responses to T3

**Fig. 2:**
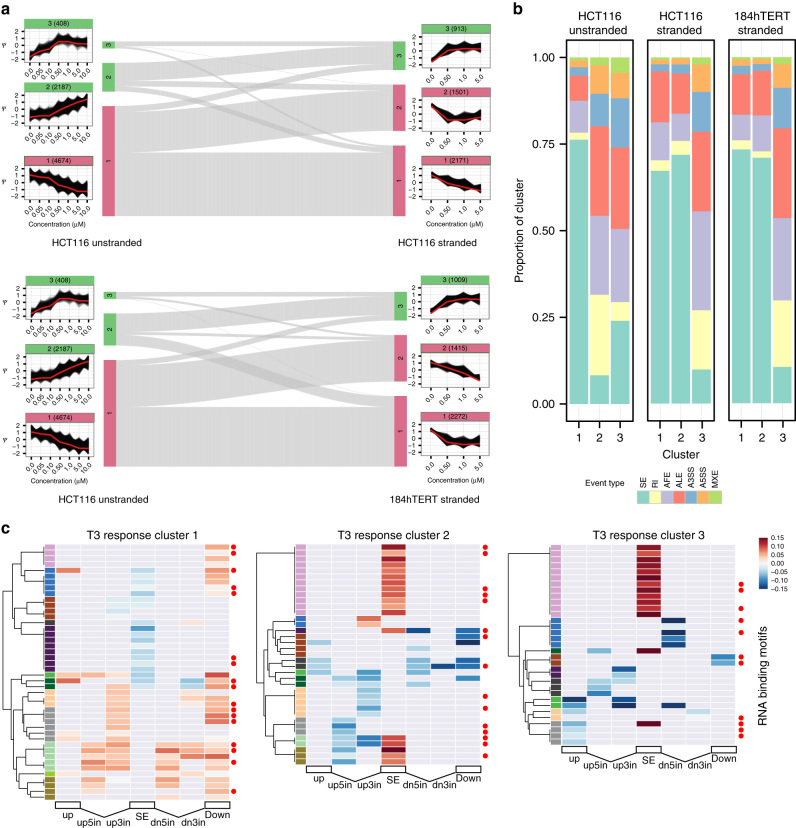
**CLKi responsive AS gene clusters analyzed by event type.** **a** PSI cluster profiles and relationships between HCT116 and 184hTERT cell types. The schematic is organized as a sankey diagram in the center, showing the relationships between AS events within clusters (*vertical bars*, with cluster number), pairwise in each of the three data sets. Events associated with increasing PSI have a *green colored bar*/*title*, those with decreasing PSI a *red colored bar*. The *gray lines* show the relationship between common members. The PSI vs. T3 dose response plots are shown aligned to the cluster they represent. *Black lines* represent AS event PSI profiles. The *vertical axis* represents standardized PSI score. The *horizontal axis* represents T3 concentration (µM). *Red lines* are cluster eigen-events. The number of events in each cluster is shown in *parentheses* in the cluster label, which is colored *green* for clusters that increase in PSI with increasing T3 concentration and *red* for clusters that decrease with T3. **b** Cluster event type proportions. The proportion of MISO event types in each cluster is shown as a *stacked bar plot*. The event key is shown below. **c** Heatmap showing RBP motifs with a significant density difference in PSI cluster 1 (monotonic PSI decrease cluster), 2 and 3 (PSI increase clusters) SE sequences compared to other PSI clusters for HCT116 unstranded RNA-Seq ∆SE events. Only one motif per RBP family is shown, for full display with labels see Supplementary Figs. 21, 22, 23. *Horizontal axis* represents seven SE event sequence regions analyzed: up (upstream exon), up5in and up3in (5′ and 3′ sections of the upstream intron), SE, dn5in and dn3in (5′ and 3′ sections of the downstream intron), and dn (downstream exon). Cells with non-significant differences are colored *gray*. Colored cells represent a positive (*red*) or negative (*blue*) effect size. Color bars on the *left* of vertical axis represent a possible motif cluster assignment based on significance and effect size patterns. *Red dots* indicate splicing factors associated with CLK family proteins

To define genomic features associated with CLK regulation, we first identified a core set of AS events undergoing monotonic response to CLKi by performing WGCNA^28^ clustering on the PSI dose profiles in an event class agnostic manner (Fig. 2a, Supplementary Fig. 17, Supplementary Data 15). In the HCT116 unstranded RNA-Seq, 28 distinct PSI profile clusters were identified, and 7 clusters were found for each of the two stranded (HCT116, 184hTERT) RNA-Seq data sets. In each case, two or three dominant clusters containing ≥10% of clustered AS events (2187 and 4674 events, HCT116 unstranded RNA-Seq), with a variable number of minor clusters (25–408 events, HCT116 unstranded RNA-Seq) were observed. Strong similarities in clustered PSI response patterns were observed between the HCT116 unstranded/stranded RNA-Seq and 184hTERT cell type RNA-Seq (Fig. 2a). Proportions of consistent T3 treatment ∆AS event responses were greater than expected by chance between the stranded and unstranded HCT116 (0.78); unstranded HCT116 and 184hTERT (0.80) and stranded HCT116 and hTERT (0.89) RNA-Seq data sets (*p* < 0.001 permutation test). The monotonic short-duration CLKi response AS events thus define a set of exons and transcript elements proximately regulated by CLK. The common PSI patterns also show that AS response to CLKi is similar between the two different cell types. A strong bias in AS event types was found within the major clusters (Fig. 2b, Supplementary Fig. 18). Clusters with a decreasing PSI response to T3 (Cluster 1 for HCT116 unstranded, 1 and 2 for the HCT116 and 184hTERT stranded RNA-Seq, Fig. 2a) were dominated by SE events, whereas clusters with an increasing PSI response to T3 showed over-representation of alternative last exon, alternative first exon, and RI events (*p* < 0.05 one-tailed hypergeometric test, Supplementary Data 16). This is consistent with the observation that SE events tend to decrease in PSI in a T3 dose-dependent manner, whereas RI events tend to increase in PSI with T3 treatment. The consequences for transcription (Supplementary Fig. 19, Supplementary Data 17, 18, 19) and protein domains (Supplementary Fig. 20) are detailed in the Supplementary Information.

To identify CLK-associated genome sequence features, we next enumerated splicing regulator binding patterns among clustered AS events, focussing on SE events from monotonic response clusters 1–3 (having the greatest number of AS events) from the HCT116 unstranded RNA-Seq analysis. We searched seven regions on each transcript comprising constitutive and alternative exons, along with regions of the intermediate introns and calculated the density of 128 human RBP motifs from cisBP-RNA^29^. We observed distinct and mirror image patterns of RBP motif enrichment (two-sided unequal-variance *t*-test on ranked data, *p* < 0.05) in the SE of the AS event and surrounding introns and upstream/downstream exons. These motifs include many known CLK-interacting RBPs. Within cluster 1, the SE exhibits reduced motif density (21 motif supersets) compared with the surrounding introns and upstream/downstream exons that have a significantly higher RBP motif density (20 motif supersets) (Fig. 2c, Supplementary Fig. 21, Supplementary Data 20). Conversely, PSI clusters 2 and 3 show a mirror image pattern of RBP motif density increase in SE, and reduced motif density in the surrounding regions (Supplementary Figs. 22, 23). In addition to RBP motif density, which was corrected for exon length, SE in cluster 1 also tend to be shorter than SE showing stronger inclusion in cluster 2 (Supplementary Fig. 24). This suggests that the conserved responses of CLKi are in part codified through RBP motif density and exon length and provides a means for predicting the consequences of CLK action in the genome.

### CLKi results in CG formation

**Fig. 3:**
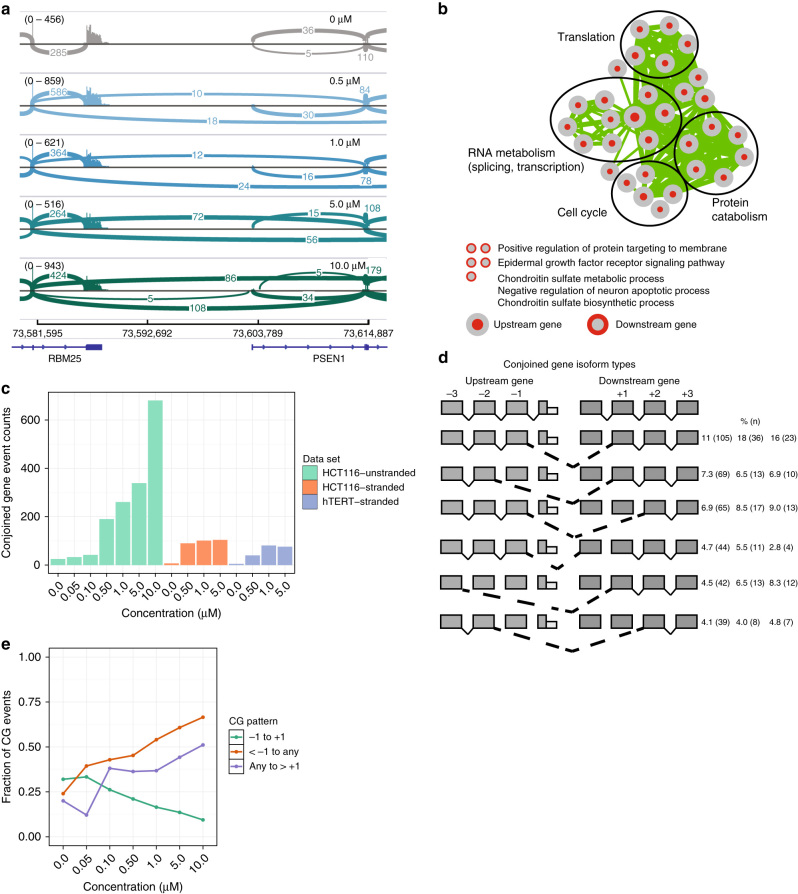
**Characteristics of CG transcripts induced by inhibition of CLK.** **a** IGV-generated sashimi plot^62^ of the RBM25-PSEN1 CG. Plots for T3 treatment concentrations of 0.0, 0.5, 1.0, 5.0, and 10.0 µM are shown from *top* to *bottom*. The control sample plot is colored *gray*, and the treated sample plots are colored according to T3 concentration. RefSeq gene annotations are shown in *blue* at the *bottom* of the plot along with chromosome 12 coordinates (bp). For each sample, the *y*-axis represents read coverage normalized by (1,000,000/total reads), and the value range is indicated between *brackets*. Arcs connecting exons represent reads spliced across introns, with the number of spliced reads annotated over the line. Only arcs representing at least five reads are shown. **b** Enrichment map for genes involved in CGs in the HCT116 unstranded RNA-Seq data set. Each node represents a GO BP gene set. BP enriched in CG upstream partners have *red cores*, while BP enriched in downstream partners have *red outer rings*. Edge thickness indicates the level of CG partner overlap between gene sets. **c** CG counts per RNA-Seq library as detected by a modified deFuse classifier. **d** Top CG splicing patterns for the HCT116 unstranded RNA-Seq data set. Schematic shows the most abundant conjoined splicing relationships between in-cis gene pairs. Right of figure, percentage of all CG events (number of events in class) for HCT116 unstranded, HCT116 stranded, and 184hTERT stranded libraries, respectively. **e** Dose-dependent CG splicing pattern proportions across T3 concentrations for the HCT116 unstranded RNA-Seq data set. As T3 concentration is increased, more exons are skipped. See panel **d** for splicing patterns

An unanticipated observation from CLKi was a dose-dependent increase of CG transcripts detected by gene fusion analysis of the RNA-Seq libraries^30^ (Methods, Supplementary Table 4). We observed many instances of splicing between adjacent genes located *in cis* on the same genomic strand in T3-treated RNA-Seq libraries (e.g., *RBM25-PSE*N1, Fig. 3a). We observed a common pattern of T3 dose-dependent CG prevalence (Fig. 3c, Supplementary Data 21), and importantly with response characteristics showing high sensitivity to CLKi (e.g., the HCT116 unstranded RNA-Seq data set shows the CG transcript abundance increases >4-fold at the 0.50 µM concentration, compared to 0.10 µM). CLKi with T3 produced more CG transcripts than *CLK* knockdown (∼7-fold increase for 0.50 µM T3 over *CLK1+CLK2+CLK3* siRNA, Supplementary Table 4, Supplementary Data 22), possibly due to limited knockdown of *CLK* by RNAi. Nevertheless, the rise in CG events with *CLK* RNAi reproduces the phenomenon and the siRNA-induced events are largely a subset of the T3-induced CG events. Moreover, treatment with the weaker KH-CB19 inhibitor reveals a small number of CG events, all of which are a subset of the T3-induced events (Supplementary Fig. 7f, Supplementary Data 5).

We validated the structure and occurrence of CG transcripts with two orthogonal methods, first using genome-wide PacBio long read sequencing^31^ (Supplementary Table 5) and second by targeted PCR-sequencing of selected events (Supplementary Table 7). The targeted PCR-sequencing validation observed 40/52 selected events (77%, Supplementary Data 23), whereas the proportion of CGs with PacBio sequencing read support was lower, 116/205 (57%) in HCT116 stranded libraries and 344/988 (35%) in HCT116 unstranded libraries (Supplementary Data 24). Many of the CGs detected in the validation data set (22 of 42, 52%) were also detected in untreated samples, but at much lower levels, underscoring their biological relevance. CG isoform expression (normalized to a sequenced housekeeping gene) was compared across T3 treatment concentrations to verify the dose-dependent effect of T3 on CG formation (Supplementary Fig. 25, Supplementary Data 25). CG expression distributions show a dose-dependent increase in both HCT116 and 184hTERT cells. We note that only ∼1/3 of CG transcripts result in an in-frame coding fusion between partner genes.

We next asked the extent to which CGs are shared in common between different cell types by generating a unique subset of upstream–downstream CG partner pairs for each data set. These sets ignore variation in donor and acceptor splice sites from the same CG partners, as these differences can be considered to arise from different isoforms of the same CG. These sets were used to determine the set of CGs common between cell types and exclusive to each cell type (Supplementary Fig. 26). Only 15 of 117 (12.8%) 184hTERT CG calls were not present in the HCT116 CG sets. Only 9 of 161 (5.6%) calls from the HCT116 stranded RNA-Seq data set were not present in the other CG sets. Overall, the majority of both stranded RNA-Seq data set CGs overlap with those in another data set, although cell type-specific gene expression differences account for some degree of non-overlap between HCT116 and 184hTERT cells (Supplementary Fig. 27).

The presence of a limited set of genes susceptible to increased CG transcription upon CLKi prompted us to investigate whether distinct features are associated with CG pairs. We first investigated the possibility that upstream and downstream CG partners are involved in related BP by performing functional enrichment analysis on upstream and downstream gene partners separately (Fig. 3b, Supplementary Fig. 28, Supplementary Data 26). Statistically significant biological pathways were detected in upstream partners, whereas downstream CG partners showed no clear pattern of function enrichment. Interestingly, the dominant functions of upstream CG partners involve RNA processing and RNA splicing (e.g., Fig. 3b), with additional networks of cell cycle, protein catabolism, and translation also observed. Similar BP were found enriched in upstream CG partners in the stranded HCT116 and 184hTERT libraries (Supplementary Fig. 28) and differential splicing analysis (Fig. 1e, Supplementary Fig. 14, Supplementary Data 14), indicating this to be a conserved pattern of gene pairings.

### CLKi-dependent CGs occur in preferred genomic contexts

The pattern of enrichment in 5′ CG gene partners suggested other genomically encoded features may account for CG transcription and we therefore systematically explored the relationships between CG formation and genomic features.

The most commonly observed CG splicing pattern involves the removal of RNA between the second-to-last and second exons of the upstream and downstream CG participants, respectively (Fig. 3d, Supplementary Data 27), consistent with CG transcripts reported in RNA databases^32,33^. We compared the proportion of CGs that follow this common splicing pattern to the proportion of CGs that skip more than one exon in either the upstream or downstream CG participants across T3 concentrations (Fig. 3e, Supplementary Fig. 29). Loss of exon recognition due to CLKi promotes the skipping of exons proximal to the intergenic region in a dose-dependent manner, i.e., with increasing inhibition of CLK more exons are skipped in the CG transcripts formed (Fig. 3e).

**Fig. 4:**
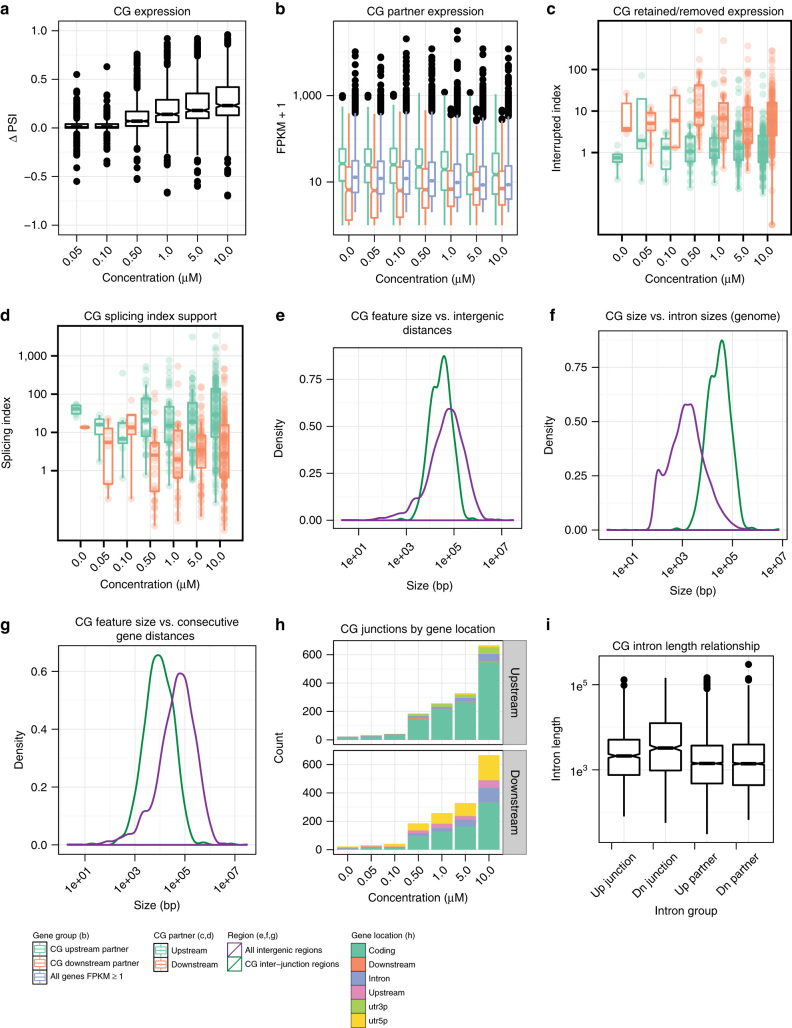
**Expression and genomic features of CGs for HCT116 unstranded libraries.** See Supplementary Figs. 30, 33 for HCT116 stranded and 184hTERT data set plots. **a** CG ∆PSI boxplots, per T3 treatment concentration. **b** Boxplots of FPKM values for CG upstream and downstream participants, compared to all genes with FPKM ≥ 1 (i.e., expressed genes). **c** Boxplots of interrupted indices for CGs across T3 concentrations. Interrupted indices are calculated as the ratio of coverage between the portions of a gene retained and removed by CGs (Supplementary Fig. 32a). Boxplots are shown for the upstream and downstream CG participants. **d** Boxplots of splicing indices for CGs across T3 concentrations. Splicing indices are calculated as the number of concordant read pairs spanning a CG splice junction in a CG participant, divided by the number of CG splice junction spanning reads that support the presence of a CG (Supplementary Fig. 32b). Boxplots are shown for the upstream and downstream CG participants. **e** Density plot of CG splice junction distances vs. gene distances in the genome. **f** Density plot of CG splice junction distances vs. intron lengths of multi-exonic protein coding genes in the genome. **g** Density plot of CG participant distances vs. consecutive gene distances in the genome. **h** Barplots showing the number of CG splice junctions falling within different annotated gene locations, across T3 concentrations. Barplots for the upstream and downstream CG participants are shown. **i** Boxplots of intron lengths for introns adjacent to the upstream and downstream CG splice junction, and all introns in upstream and downstream CG gene partners

We next examined the relationship between canonical transcripts and CG transcripts. We calculated CG ∆PSI values and compared their distributions across each treatment concentration (Fig. 4a, Supplementary Fig. 30a, Supplementary Data 28, 29, 30). CGs in both HCT116 and 184hTERT cells show a dose-dependent PSI increase. A clear increase in PSI value changes can be seen at the 0.5 µM concentration (*p* < 0.0001 ranked two-sided unequal-variance *t*-test, 7-fold increase 0.50 vs. 0.10 µM). CLKi clearly increases the proportion of CG to wild-type transcripts, in a dose-dependent manner. We then compared CG PSI changes to the expression of non-conjoined upstream transcripts, and found that upstream gene non-conjoined transcription decreased with increased CG PSI (Supplementary Fig. 31). This indicates that CGs form at the expense of canonical transcript isoforms, rather than resulting from increased transcription of the upstream partner.

However, gene expression of upstream CG participants is generally higher than other expressed genes, while downstream participants have lower expression (Fig. 4b, *p* < 0.0001 ranked two-sided unequal-variance *t*-test for 0.50 µM T3 HCT116 unstranded RNA-Seq, Supplementary Fig. 30b). Additionally, the ratio of read coverage between gene regions retained and removed by CGs is much higher in downstream participants (Fig. 4c, *p* < 0.0001 ranked two-sided unequal-variance *t*-test, 8-fold increase in median for 0.50 µM T3 HCT116 unstranded RNA-Seq, Supplementary Figs. 30c, 32a). Further, when considering CG splice junction spanning read pairs, the ratio of wild-type to CG read pairs is much higher in upstream participants (Fig. 4d, *p* < 0.0001 ranked two-sided unequal-variance *t*-test, 8.3-fold increase in median for 0.50 µM T3 HCT116 unstranded RNA-Seq, Supplementary Figs. 30d, 32b). Thus, CG transcription generally occurs from a more highly transcribed 5′ partner to a less transcribed 3′ partner. As a result, a greater proportion of downstream gene expression is modulated by CG transcription.

Exon and gene lengths are related to the efficiency of splicing reactions and we explored this question next. We compared the distance between the donor and acceptor splice sites connecting the upstream and downstream CG participants to the distances between consecutive genes in the genome (Fig. 4e, Supplementary Fig. 33a), as well as introns in multi-exonic genes (Fig. 4f, Supplementary Fig. 33b). CG splice junction distances are limited in length compared to consecutive gene distances (*p* < 0.0001, ranked two-sided unequal-variance *t*-test) and are generally longer than gene introns (*p* < 0.0001, ranked two-sided unequal-variance *t*-test). We next compared CG participant distances to consecutive gene distances in the genome (Fig. 4g, Supplementary Fig. 33c). The distances between CG participants are typically shorter than other consecutive genes (*p* < 0.0001, ranked two-sided unequal-variance *t*-test). Thus, CG formation appears to be partly determined by the distance between the participant genes.

Finally, we examined the CG junctions spanning six gene regions (Fig. 4h, Supplementary Fig. 33d). The upstream junctions lie predominantly within coding regions, more so than the downstream junctions (1.6-fold more in 0.50 µM T3 HCT116 unstranded RNA-Seq). Downstream CG splice junctions are located within the 5′ UTR more often than upstream junctions are located within the 3′ UTR (3.1-fold more in 0.50 µM T3 HCT116 unstranded RNA-Seq). They are also more often found at exons proximal to the intergenic region than upstream CG splice junctions (Fig. 3d). Recognition of splice sites in the upstream CG participant may therefore rely on CLK activity to a greater degree than splice sites in the downstream participant. As previously noticed for endogenous CG transcription^33^, introns located next to CG splice junctions in the downstream participant are longer than other introns in either the upstream or downstream participants (Fig. 4i, *p* < 0.001 ranked two-sided unequal-variance *t*-test, Supplementary Fig. 33e).

### CG transcription reflects RNA processing factor activity

In addition to a defects in terminal exon recognition, CG formation is also likely to involve aberrant 3′-end processing. Furthermore, since CLK family proteins regulate the activity of SR proteins and are found within complexes containing 3′-end binding processing factors, we examined whether these motifs are enriched or depleted in regions surrounding CG junctions. We investigated regulatory signals in 3′ ends of transcripts by first identifying the annotated locations of terminal poly(A) sites in the genome. Upstream CG participants have a greater number of annotated poly(A) sites, compared to all genes (*p* < 0.0001 ranked two-sided unequal-variance *t*-test, rank biserial correlation effect size: 0.16). However, we did not observe a significant difference in the proportion of terminal poly(A) sites with canonical A(A/U)UAAA poly(A) signals, CFIm-bound UGUA motifs, or U/GU-rich regions between upstream participants and all genes. Nevertheless, CG upstream partner genes appear to possess the intrinsic potential for alternative poly (A) site regulation.

**Fig. 5:**
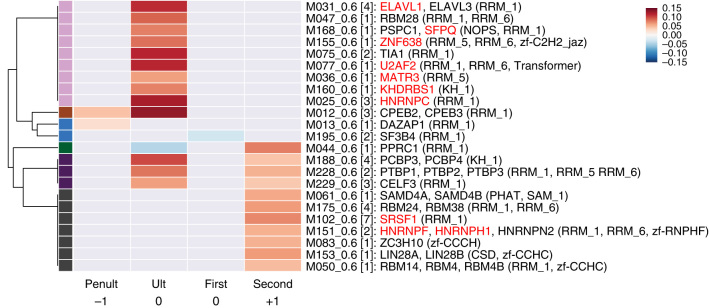
**Heatmap showing significant RBP motif density differences in CG sequences.** Heatmap is for all HCT116 and 184hTERT CGs compared to non-CG sequences. *X*-axis represents four CG sequence regions: penultimate (−1 exon) exon, last exon (0, terminal exon of upstream participant), first exon downstream (0, first exon of downstream participant), second exon (+1, downstream partner). Only one motif per RBP family is shown, the number of motifs in *square brackets*. *Y*-axis shows the RBP motif id and RBP name (highlighted *red*: CLK interactors) and protein domains associated with the listed motif. Cells with non-significant differences are colored *gray*. Colored cells represent a positive (*red*) or negative (*blue*) effect size. Color bars on the left *y*-axis represent a possible motif cluster assignment based on significance and effect size patterns

We next asked whether differences in RBP motif density and 3′ processing signals could be found in CG junction regions. We first conducted a motif analysis of the terminal exons of upstream partners and the initial exons of downstream partners, correcting for exon length as was done for ∆PSI AS clusters. Strikingly, CLK-dependent CG partner genes show statistically significant motif enrichment (*p* < 0.05 ranked two-sided unequal-variance *t*-test, Supplementary Data 31) in the last exon of upstream partners and the second exon of the downstream partners (Fig. 5, Supplementary Fig. 34). This suggests a potential short list of RBPs that could be involved in the biological process of CG formation, which we systematically assay later.

**Fig. 6:**
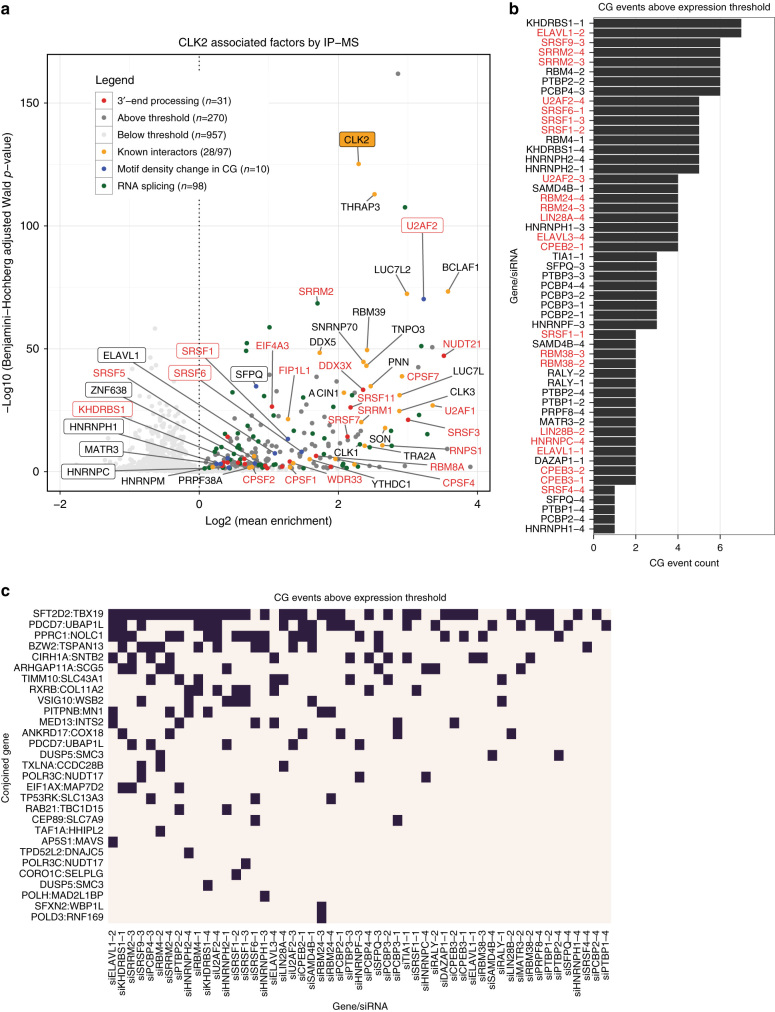
**Proteins associated with CLK2 and implicated in CG expression.** **a** Volcano plot ranking proteins associated with CLK2 IP identified by four pull downs of N-terminal FLAG tagged, one untagged CLK2 and one control empty vector, determined by TMT mass spectrometry. The log_2_ mean fold enrichment (*horizontal axis*) and enrichment *p*-value were determined by linear model fits to MS determined peptide abundance. Known CLK2 interactors^34,35,36^, *orange dots*; known spliceosome-associated proteins (PantherGO^52^), *green dots*; factors associated with RNA-binding motifs enriched in CG exons are boxed and their dots *blue*; factors associated with 3′-end processing highlighted in *red*. Threshold is adjusted to <0.05 and log_2_(mean enrichment) >0. **b** Number of CG events above expression threshold after siRNA knockdown. siRNA to splicing factors (*vertical axis*, highlighted *red*: 3′-end processing factors) assessed by a quantitative, targeted NGS sequencing panel for specific CG events was used to quantify CG expression in relation to non-targeting controls. The plot displays the number of CG events per knockdown condition, expressed more than 2 SD above the mean of ≥3 non-targeting controls or the number of CG events per knockdown condition where CG expression in the non-targeting controls was undetectable (see also Supplementary Fig. 36). **c** Heatmap showing distribution of individual CG event occurrence (*vertical axis*) above the control expression threshold (as in panel **b**). The presence of a *black square* indicates one or more CG events

Prompted by the CG exon motif enrichment observed, we investigated whether the known CLK protein complex associations^34–37^ with relevant splicing factors and 3′-end processing factors could be detected in the mammalian cell line used here (HCT116). We performed immunoprecipitation-mass spectrometry (IP-MS) identification of N-terminal-tagged CLK2-associated proteins (4 FLAG-tagged replicates, one untagged CLK2), using tandem mass tags (TMT) mass spectrometry and observed highly significant enrichment (Fig. 6a, Supplementary Data 32) of both known CLK-interacting proteins (28/97 known, B-H adjusted Wald *p* < 0.05) including CLK1 and additional splicing factors (98/270 total enriched, B-H adjusted Wald *p* < 0.05). GO (GO term) analysis revealed (Supplementary Fig. 35) the most strongly enriched proteins in the IP-MS experiment were 3′-end processing factors, transcription termination factors and spliceosomal proteins, as expected. Notably, several factors associated with enriched or depleted motifs in CG exons were found in this experiment (boxed in Fig. 6a).

To test the requirement for individual factors, we first systematically tested the efficacy of siRNA knockdown of 235 individual siRNAs representing 60 motif-enriched factors (from the short list previously obtained, Supplementary Fig. 34) and identified 103 effective siRNAs (RQ < 0.4) targeting 39 proteins. In most cases where >2 effective siRNAs represented the same gene, the 2 most effective were used. Some genes were only represented by a single siRNA as either other siRNAs were ineffective or the knockdown experiment did not yield enough mRNA to proceed. The effect of each selected siRNA was individually tested in replicate experiments for the ability to promote CG formation by targeted singleplex RT-PCR followed by sequence validation and analysis on the MiSeq system for the panel of 52 CG events (validated to be T3 dose-dependent), for each factor examined (Supplementary Table 7). A total of 33 genes tested (55 siRNAs) exhibited significant CG expression (>2 SD above mean control condition CG expression where >2 CGs were detected in controls, or >1 CG expressed where no CGs were detected in controls, Supplementary Fig. 36, Supplementary Data 33), with multiple CG events detected above controls (Fig. 6b, c) in many cases.

We observed that many 3′ UTR-binding or processing factors (highlighted red, Fig. 6b) exhibited multiple CG events per siRNA, including genes such as SRRM1/2 (validated independently, Supplementary Fig. 37, Supplementary Data 34), known to be involved in 3′-end processing and poly(A) cleavage, independent of a role in the exon junction complex. Together these data provide support for CG expression associated with CLKi via the functions of 3′-end factors and SR proteins associated with CLK containing complexes.

## Discussion

We have synthesized a novel small molecule inhibitor of CLK family proteins, named T3, which is structurally distinct and two log orders more potent and selective than previously described CLK inhibitors, in addition to having favorable cell permeability and stability. T3-mediated CLKi results in loss of exon recognition, with increased exon skipping and RI. By applying graded inhibition and clustering AS based on response pattern, we have defined globally the sequence features associated with CLK splicing functions for the first time.

An unanticipated effect of CLKi was the discovery of a dose-dependent increase of CGs—which are splice transcripts formed between two adjacent genes on the same genomic strand and orientation. CG formation through transcriptional read-through can occur naturally in cells and has received some attention in the literature^32,33^, although their regulatory and functional significance remains unclear. We show here that loss of CLK activity is associated with CG transcription and we define the genomic context in which CLK-dependent CG transcription occurs. In approximately 1/3 of cases, the CG pairs result in an in-frame transcript and thus potentially fusion proteins will be produced from these events. The consequences of CG transcription could be reduced expression of 5′ and 3′ canonical transcripts and the generation of neomorphic chimeric proteins. The observed CG transcripts in fact occur at the expense of canonical transcription of the upstream partner gene, i.e., CG formation should reduce the expression of the upstream partner genes, with possible downstream consequences. The number and pattern of terminal/initial exons skipped in the formed CGs is dependent on the strength of CLKi and shows a similar inhibition sensitivity to AS events. This suggests one mechanism by which CLK may regulate CG formation, i.e., directly through RS domain or other phosphorylation events in recognition of terminal exons. The genomic sequence context of CG pairs shows distinct features related to intron length, gene pair distances, and RBP motif density, which suggests that CG formation is partly defined by sequence context. Many splicing factors, including 3′-end processing factors have been described in association with CLK proteins and we confirmed by IP-MS in the cell line used here that several of the CG exon motif-associated factors as well as known SR proteins associate with CLK2. Notably, the most strongly enriched CLK2-associated protein families are 3′-end processing/binding factors and poly adenylation associated factors. Through systematic siRNA knockdown of 39 of the CLK2 and CG motif-associated factors, we demonstrate that 33 genes (55 siRNAs) result in significant increases in CG formation by targeted RNA-Seq of a selected panel of T3 dose-dependent CGs. This points to a possible involvement of these particular RBPs in CLK2-mediated terminal exon recognition and/or 3′-end processing and suppression of CG formation. Thus, we furnish evidence supporting a novel role of CLK in regulation of CG splicing and gene boundaries, providing a route to future exploration of this process and for pharmacological manipulation.

In terms of the potential applications of T3 in disease, previous reports demonstrate that knockdown of CLK2 inhibits growth of breast tumors with overexpresseed CLK2 in cell and animal models of the disease, correlating with splicing alterations^12^. Inhibition of CLK1 by TG003 or KH-CB19 blocks proliferation^13^ similar to T3 treatment, suggesting that T3 may be relevant in the treatment of diseases, such as breast and kidney cancers, where respective CLKs are deregulated.

## Methods

### Synthetic route and characteristics of CLK inhibitor T3

An overview of the synthetic intermediates is shown in Supplementary Fig. 1a.

Methyl 2-(4-((6-iodoimidazo[1,2-a]pyridin-2-yl)carbamoyl)phenyl)-2-methylpropanoate (1) was prepared by similar method described in Terao et al.^38^ A mixture of methyl 2-(4-((6-iodoimidazo[1,2-a]pyridin-2-yl)carbamoyl)phenyl)-2-methylpropanoate (1) (2.1 g, 4.53 mmol) and 2 N NaOH (11.33 ml, 22.67 mmol) in MeOH (25 ml)-THF (12 ml) was stirred at 50 °C for 3 h. After cooling, the mixture was acidified with 2N HCl and diluted with water (50 ml). The precipitate was collected by filtration, washed with water, and dried in vacuo to give 2-(4-((6-iodoimidazo[1,2-a]pyridin- 2-yl)carbamoyl)phenyl)-2-methylpropanoic acid (2) (1.440 g, 3.21 mmol, 70.7%) as a off-white solid.

HATU (1.777 g, 4.67 mmol) was added to a mixture of 2-(4-((6-iodoimidazo[1,2-a]pyridin-2-yl)carbamoyl)phenyl)-2-methylpropanoic acid (2) (1.40 g, 3.12 mmol), 1-methylpiperazine (0.468 g, 4.67 mmol), and N-ethyldiisopropylamine (1.089 ml, 6.23 mmol) in DMF (15 ml) at room temperature. After stirring at room temperature for 2 h, the mixture was diluted with sat.NaHCO_3_ aq. and extracted with EtOAc. The organic layer was separated, washed with water and brine, dried over MgSO_4_ and concentrated in vacuo. The residue was purified by column chromatography (NH silica gel, eluted with 50–100% EtOAc in hexane) to give N-(6-iodoimidazo[1,2-a]pyridin-2-yl)-4-(2-methyl-1-(4-methylpiperazin-1-yl)-1-oxopropan-2-yl)benzamide (3) (1.560 g, 2.94 mmol, 94%) as a off-white solid.

Pd(dppf)2Cl_2_ *·*CH_2_Cl_2_ (0.231 g, 0.28 mmol) was added to a mixture of N-(6-iodoimidazo[1,2-a]pyridin-2-yl)-4-(2-methyl-1-(4-methylpiperazin-1-yl)-1-oxopropan-2-yl)benzamide (3) (1.5 g, 2.82 mmol), 4-(4,4,5,5-tetramethyl-1,3,2-dioxaborolan-2-yl)pyridine (0.868 g, 4.23 mmol), and Cs_2_CO_3_ (1.839 g, 5.65 mmol) in DME (9 ml)-water (1.5 ml)-DMF (6 ml) at room temperature. The mixture was heated at 100 °C for 30 min under microwave irradiation. After cooling, the mixture was diluted with water and extracted with EtOAc—THF three times. The combined organic layers were washed with brine, dried over MgSO_4,_ and concentrated in vacuo. The residue was purified by column chromatography (NH silica gel, eluted with 0–10% EtOAc in hexane) to give 4-(2-methyl-1-(4-methylpiperazin-1-yl)-1-oxopropan-2-yl)-N-(6-(pyridin-4-yl)imidazo[1,2-a]pyridin-2-yl)benzamide (T3) (0.990 g, 2.051 mmol, 72.7%) as a pale yellow solid. Recrystallization from MeOH-EtOAc-IPE gave off-white crystals.

M.p.: 240 °C 1H-NMR (300 MHz, DMSO-d6) 𝛿11*.*28 (s, 1H), 9.22 (s, 1H), 8.67 (d, *J* = 5.0 Hz, 2H), 8.38 (s, 1H), 8.11 (d, *J* = 7.9 Hz, 2H), 7.53–7.85 (m, 4H), 7.34 (d, *J* = 7.8 Hz, 2H), 2.60–3.80 (br, 4H), 2.05 (s, 3H), 1.60–2.40 (br, 4H), 1.46 (s, 6H); HRMS (*m*/*Z*): [M+H]+ calculated for C28H30N6O2, 483.2503; found, 483.2469; analysis (% calcd, % found for C28H30N6O2); C (69.69, 69.30), H (6.27, 6.34), N (17.41, 17.17).

ChemDraw files of T3 and the synthetic route are included in Supplementary Data 37, 38.

### RNA-Seq acquisition and data processing

Total mRNA was isolated and strand-specific or non-stranded-specific whole transcriptome RNA-Seq was performed by the Michael Smith Genome Sciences Centre, BC Cancer Agency, Vancouver, Canada, on an Illumina HiSeq 2000 or 2500 machine. RNA-Seq libraries were aligned to the hg19 (GRCh37) reference genome assembly using the GSNAP aligner^39^. Aligned libraries had mate-pair information fixed, were removed of potential PCR duplicates, and sorted using SAMtools^40^.

Differential splicing detection was performed using MISO^24^ using the appropriate configuration for stranded or unstranded RNA-Seq libraries. AS events with a Bayes factor < 20, ∆PSI < 0.1, 0 reads supporting the inclusion or exclusion isoform, or <10 reads supporting either of the event isoforms were removed. T3-treated samples were compared to control DMSO vehicle-treated samples. CLK siRNA transfected samples were compared to a control NT3 siRNA transfected sample. The NT3 siRNA transfected sample was also compared to a vehicle Lipofectamine 2000-treated sample as an additional control.

CG discovery was carried out using a version of the deFuse gene fusion detection software^30^ with the classifier modified by removing the est_breakseqs_percident and break-seqs_estislands_percident features. Inclusion of these classifier features can result in reduced probability for CG (read-through) fusion calls. CGs were identified by selecting deFuse gene fusion calls where both participating genes were located on the same strand of the same chromosome and where deletion = “Y”, expression ≥ 50 reads for both genes, splice score = 4, exonboundaries = “Y”, probability ≥ 0.9. These filters were chosen to produce a set of CG event calls that are likely due to splicing as opposed to genomic aberrations, and occur with a high probability. deFuse analysis of CG-targeted sequencing libraries was performed using a similar set of filters, with the exception of probability ≥ 0.5, and no expression filters. The T3 concentration curve replicate experiments also used a similar set of filters, excluding the expression filter. CG features, including splicing index and CG splice junction gene location, were extracted from the deFuse output files.

Cufflinks^41^ was used to quantify gene expression FPKM values using the --frag-bias-correct, --multi-read-correct, and --GTF options.

### PacBio acquisition and data processing

Total mRNA was isolated for HCT116 cells and fractionated by size (<1.5 kb, 1.5–3 kb, >3 kb) and each fraction individually sequenced using SMRT technology on a PacBio RS by Tomy Digital Biology Company, Ltd (Tokyo, Japan). All the respective size fractions of a sample were pooled and aligned to the hg19 (GRCh37) reference genome assembly using the GMAP aligner^42^. Aligned reads were filtered to remove those with aligned proportion less than 90%, or less than 80% sequence identity with the reference.

PacBio reads were used to validate deFuse-identified CGs by first extracting exon junction positions from aligned PacBio reads. A PacBio read was considered to support a deFuse CG if two consecutive read exon junctions matched the reported CG splice junction coordinates.

### CG PSI quantification

CG isoform annotations were generated using deFuse predictions as well as potential CGs identified in the PacBio libraries. As deFuse was designed to use paired-end RNA-Seq reads, an alternative method for CG detection was necessary for the PacBio data. CG transcripts were detected by selecting reads that mapped across two different genes located on the same chromosome strand. For a PacBio read to be considered as “mapped” to a gene for the purposes of CG detection, at least three exon junctions within a read must match an exon junction belonging to a single gene in the GENCODE version 19 level 1 and 2 transcript annotations. Cases where one gene is encapsulated within another gene (e.g., a miRNA located within the intron of another gene) are not considered CGs (Supplementary Data 28).

Inclusion (CG) isoform annotations include the CG splice junction-adjacent exons from the upstream and downstream CG participants, and any detected intergenic exons. Exclusion (wild-type) isoform annotations include the CG splice junction-adjacent exon and up to one downstream exon, if available, from the upstream CG participant. The generated CG isoform annotations were input to MISO, and PSI values were calculated for each CG event.

### CG sequence context analysis

Gene annotations were collected from Ensembl version 69. Genome-wide and CG-associated genomic features were extracted and compared (e.g., intergenic distances, intron lengths). CG-specific data, e.g., the interrupted (Supplementary Fig. 32a) and splicing indices (Supplementary Fig. 32b), were extracted from the deFuse result files.

Here, the definition of an interrupted index is slightly different from the deFuse output. Whereas deFuse defines the interrupted index as the ratio of coverage before and after the break-point in a fusion participant, we define the interrupted index as the ratio of coverage between the retained and removed portion of a gene participating in a CG (Supplementary Fig. 32a). Therefore, the interrupted indices for both the upstream and downstream CG participants maintain a consistent interpretation: the ratio of a gene’s total vs. wild-type expression.

### CG-associated RBP knockdown analysis

CGs in RNAi libraries were detected by deFuse as described above. CG expression values were calculated by counting the number of reads supporting a CG as reported by deFuse. CG expression values in each RNAi library were upper-quartile normalized. Detected CGs were broken into three groups: those detected in at least three control libraries, those detected in zero control libraries, and all other CGs. Expression values for each CG were collected from all siRNA treatments, then filtered. For CGs detected in at least three control libraries, expression values were retained if they were greater than the mean + 2 standard deviations of the control library expression values. CGs detected in zero control libraries are assumed to be unexpressed under control conditions and all expression values are retained. Other expression values are discarded. CGs passing the expression filters described above were counted for each siRNA treatment.

### CG poly(A) analysis

GENCODE^43^ version 19 poly(A) site annotations were identified for CG participants. Poly(A) site annotation counts were compared between CG participants and all genes with at least one poly(A) site annotation.

Canonical A(A/U)UAAA poly(A) signals, UGUA motifs (recognized by CFIm), and U/GU-rich downstream element (DSE) elements were located in sequences up to 50 bp, 150 bp upstream, or 50 bp downstream of the poly(A) site, respectively. For the purposes of this analysis, a DSE is defined as a sequence of at least six nucleotides, comprising uracils and interspersed with up to three non-sequential guanines. The number of terminal poly(A) site regions containing the previously mentioned sequences were compared between upstream CG partners and all genes.

### DI PSI quantification

Human DI coordinates were obtained from Boutz et al.^21^ supplemental data. DI coordinates were matched with adjacent exon coordinates from AceView^44^ to generate MISO compatible annotation files. DI annotations were input into MISO and PSI values were quantified for the HCT116 unstranded and stranded, and 184hTERT RNA-Seq data sets.

### Clustering analysis

AS and gene expression response profiles were clustered using the WGCNA R package^28^. Soft-threshold selection was facilitated by calculating the scale-free network topology model fit𝑅^2^ values for soft thresholds 1–30 using the pickSoftThreshold function. Threshold values were manually chosen by selecting values at which the scale-free topology model fit begins to plateau on the model fit vs. threshold curve. In cases where model fit𝑅^2^ values did not reach above 0.8, values above 20 were chosen, as this produced visually distinct clusters and agreed with the suggested guidelines for threshold selection^45^. WGCNA was run with networkType = “signed”, minModuleSize = 25. Similar clusters were merged using the mergeCloseModules function. Finally, a representative cluster member (e.g., “eigenevent”) was calculated for each cluster, and cluster members that did not strongly correlate with the cluster representative (Pearson correlation coefficient < 0.75) were removed.

AS events were selected for PSI profile clustering if they were differentially spliced in any of the treated samples. Events with missing PSI values were removed unless they contained only two non-consecutive missing values in the case of the HCT116 unstranded RNA-Seq data set, or only one missing value in the stranded RNA-Seq data sets. Missing values were replaced using linear interpolation. Soft power thresholds of 17, 28, and 24 were selected for the HCT116 unstranded and stranded RNA-Seq, and 184hTERT data sets, respectively. Genes selected for clustering were required to have FPKM values ≥1 in at least four libraries for the unstranded HCT116 RNA-Seq data set, and three libraries for the two stranded RNA-Seq data sets. Each gene must also have an FPKM fold change ≥2 for at least one treated library when compared with the untreated control library. Soft power thresholds of 28, 27, and 30 were selected for the HCT116 unstranded and stranded RNA-Seq, and 184hTERT data sets.

### Comparison of AS event ∆PSI tendencies between data sets

Robust linear models were estimated using PSI profiles from ∆AS events common between each pairwise combination of T3-treated RNA-Seq data sets. Huber’s T robust criterion function was used to downweight outliers. Regression model slopes were compared between each pair of data sets for each common ∆AS event. The proportion of events that have a similar regression line slope direction (i.e., positive or negative) were computed for each data set comparison.

Event proportion *p*-values were computed by randomly shuffling the event labels for the regression slopes 10,000 times and each time recomputing the proportion of common events with similar regression slope directions. *P*-values are calculated as per Phipson et al.^46^:

1 $$p=\frac{b+1}{m+1},$$

where *b* is the number of shuffled data sets with calculated proportion values greater than the original event proportion, and *m* is the number of iterations.

Cluster memberships of common ∆AS events between data sets were identified for each set of PSI clusters. Proportions of events common between the top three PSI clusters for each set of PSI clusters were computed and visualized as Sankey diagrams.

#### ∆SE affected Pfam domains

Pfam domain annotations were collected from Ensembl version 70. Domain coordinates for each transcript were converted to genomic coordinates. SE whose coordinates overlapped at least one Pfam domain annotation were marked as affecting a Pfam domain. The proportion of SE events in each AS PSI cluster that affect at least one Pfam domain was computed.

#### RNA-binding motif analysis

One hundred twenty-eight human RBP motif position weight matrices (PWMs) published by Ray et al.^29^ were obtained from the CISBP-RNA Database^29^. A total of 4551 SE events from the PSI clustering analysis were selected, and 7 regions from each event were chosen for analysis: the SE, their adjacent constitutive exons, and up to 600 bp from the introns flanking each SE. Intronic sequences are broken into two halves: upstream and downstream, and are located next to the 5′ and 3′ splice sites. Sequences from each event region were collected from the hg19 reference genome. Each of the seven regions were scanned for instances of binding motifs using the PWMs, correcting for background nucleotide rates. Matches with score ≥80% of the maximum PWM score were retained. For each binding motif and region, a “motif density”, *d*, was calculated:

2 $$d=\frac{m}{l},$$

where *m* is the number of motif matches passing the score threshold, and *l* is the length of the sequence region in base pairs (bp).

SE events were grouped according to their PSI cluster memberships. Event regions for PSI clusters 1–3 were checked for changes in motif densities. Density values for each (region, motif) pair were compared to corresponding densities in all other clusters. This comparison scheme was chosen to identify differences associated with specific response profiles, compared to other affected events. A comparison to unaffected events may detect signal assocated with affected events in general, rather than with specific response profiles. Each comparison was performed by rank-transforming the motif densities, then performing an unequal variance *t*-test. Unequal variance *t*-tests on ranked data are a robust alternative to Student’s *t*-test when assumptions of normality are violated^47^. *P*-values were adjusted using the Benjamini–Hochberg method^48^, and tests were deemed significant with an adjusted *p*-value threshold of 0.05.

Effect sizes were calculated for significant tests using the Kerby simple difference formula for rank-biserial correlation^49^. Reported effect sizes may be interpreted as the probability of a randomly selected motif density from the PSI cluster of interest being higher, minus the probability of being lower than a randomly selected density from other clusters. Positive effect sizes indicate increased motif density, while negative values indicate reduced motif density.

The motifs provided by Ray et al.^29^ include multiple, similar motifs for some proteins. Some motifs are listed for multiple similar proteins. To reduce redundancies, motifs were grouped according to RBP family as reported in Gerstberger et al^50^. For each motif group, one motif was retained and the remaining removed. If possible, a motif belonging to the representative protein family member was chosen. Selection between multiple candidate motifs was performed by choosing the motif with the greatest number of regions in which it is enriched or depleted.

For each tested PSI cluster, motifs that were enriched or depleted in at least one region were hierarchically clustered according to its pattern of enrichment and depletion. Cluster assignments were determined by grouping motifs with co-phenetic distance no greater than half the maximum distance between any two motifs. Clustered motifs were annotated using information obtained from Ray et al.^29^ and Gerstberger et al^50^.

RNA motif analysis for CGs was performed as above, except the scanned sequences were collected from the penultimate and ultimate exons of the upstream participants, and the first and second exons of the downstream participants. CG region motif densities were compared to corresponding regions from a set of 5000 randomly selected non-CG genes with at least four exons.

### GO term enrichment analysis and enrichment map generation

GO BP term enrichment for a set of genes was performed by generating functional interaction networks using the ReactomeFI Cytoscape plugin^51^. BP term enrichment was performed using genes in the resulting network and reported terms have false discovery rate controlled at 0.05. Enrichment maps were generated for ReactomeFI BP enrichment results using the EnrichmentMap Cytoscape plugin^26^. For assessment of the top BP by fold enrichment, the gene list was submitted to GeneOntology (http://www.pantherdb.org/)^52^ and the statistical overrepresentation test was used. The results were filtered by the score of the functional enrichment testing^52^ for each of the BP (cut-off, Fold Enrichment > 23.5).

### siRNA and primer sequences

The genomic locations and primer sequences to the CG panel used for targeted sequencing to assess their expression can be found in Supplementary Data 35. The siRNA sequences and their effectiveness at reducing expression of the respective target genes, as assessed by relative expression (RQ), is listed in Supplementary Data 36.

### Plasmids and cloning

Human *CLK2* cDNA was PCR cloned into pcDNA3.1(+). For the generation of the tagged expression construct, the *CLK2* cDNA was sub-cloned into pDONR221 entry vector and then shuttled by Gateway LR cloning (ThermoScientific, MA, USA) into pdcDNA-FLAG (LMBP 4704, The CABRI Consortium, http://www.cabri.org/) to produce N-terminal FLAG-tagged CLK2 constructs. NMD reporter plasmid was constructed based on the literature^53^. Briefly, the SV40 promoter and the *β-*globin genomic sequence from exon II to the PolyA signal were inserted into pGL4.13 (Promega, Madison, WI).

### In vitro kinase assays

Kinase assays for CLK1–3, DYRK1A and 1B were performed using the LANCE Ultra kinase assay system (Perkin-Elmer, Waltham, MA). All enzymes were purchased from Life Technologies (Carlsbad, CA). The kinase buffer contained 25 mm HEPES (pH 7.5), 10 mm magnesium acetate, 1 mm DTT, 0.01% BSA, and 0.01% Tween-20. The reaction mixtures contained 100 ng ml^−1^ CLK1, 30 ng ml^−1^ CLK2, 300 ng ml^−1^ CLK3, 1000 ng ml^−1^ DYRK1A, or 300 ng ml^−1^ DYRK1B. 50 nM ULight^TM^-myelin basic protein (MBP) peptide (Perkin-Elmer) was used as a substrate except that ULight-CREBtide (Perkin-Elmer) for CLK3. After incubating the enzyme, peptide, and compounds for 10 min, kinase reactions were initiated by adding ATP to a final concentration of 1 mm, followed by incubation for 45 min. The enzyme reactions were terminated by adding EDTA to a final concentration of 10 mM, and phosphorylated substrate peptides were detected using a Europium-labeled anti-phospho-MBP antibody or a Europium-labeled anti-phospho-CREB (Ser113) antibody diluted with detection buffer (Perkin-Elmer). The plates were read by an En-Vision 2102 multilabel reader (Perkin-Elmer). We defined the fluorescence signals of the reaction without enzyme as 100% inhibitory activity and those of the complete reaction mixture as 0% inhibitory activity. Curve fittings and calculations of IC50 values were performed with the program XLfit ver. 5 (ID Business Solutions Ltd., London, UK) with the maximum and minimum of the curve constrained to 100 and 0, respectively.

### Cell lines

HCT116, a transformed malignant colorectal carcinoma cell line, was obtained from ATCC and had its whole genome sequenced in our laboratory. We further authenticated a late passage in the laboratory using a panel of 48 HCT116-specific SNPs. 184hTERT is a telomerase immortalized normal mammary epithelial cell line that we have whole genome sequencing information for ref. 23. HeLa, an immortal cervical cancer cell line historically and extensively used in research, was obtained from ATCC and only utilized for in vitro studies. All cell cultures were grown in mycoplasma-free conditions and routinely tested to ensure that they are negative.

### NMD reporter assay

The reporter activity was confirmed by siRNA for NMD factor such as SMG1 and Upf1. HeLa cells were transfected in 96-well plate with the NMD reporter and control reporter plasmid (phRL-TK) (Promega, Madison, WI) using FuGENE 6 transfection reagent (Roche, Basel, Switzerland). After 48 h of the transfection, the cells were treated with T3 for 6 h. Luciferase activity was measured using Dual-Glo Luciferase Assay System (Promega), according to the manufacturer’s instructions.

### siRNA transfection and RNA isolation and expression analysis

HCT116 cells were transfected in 6-well or 96-well plates with siGenome or ON-TargetPlus Smart pools or the respective individual siRNA (Dharmacon, GE, USA) using Lipofectamine 2000 (LF2K) or RNAiMAX reagent and according to the manufacturer’s instruction (ThermoFisher/Life Technologies, Waltham, MA). When using LF2K transfection reagent, the cells were split 1 day after the first transfection and were re-seeded in 6-well plates for a second hit of siRNA to achieve maximum knockdown. When RNAiMAX was used, cells were transfected with siRNA only once and left for the duration of the experiment. Total RNA was isolated 48 h after (the second, for LF2K only) transfection using RNAeasy Mini Kit (Qiagen). Isolated total RNA was used for reverse transcription with Superscript III (Life Technologies) in the presence of oligo-dT following the manufacturer’s protocol. Real-time qPCR assays were designed using either Universal ProbeLibrary (Roche) or TaqMan gene expression assays (Applied Biosystems) or analyzed by next-generation targeted sequencing^54^ using the MiSeq (Illumina, San Diego, CA) as indicated in the results. qRT-PCR protocols were set up in 384-well plates and performed in ABI 7500 real-time PCR system (Life Technologies).

### Western blot analysis

T3-treated (3 or 6 h) or transfected (48 h) or untreated HCT116 cells were harvested and lysed in 1× sample buffer containing 0.16% SDS, 2% glycerol, 5 mm Tris-HCl (pH 6.8) and 0.0008% Bromophenol Blue, and were boiled for 5 min. Following electrophoresis on a gradient gel (5–15% or 4–12%) and transfer to a nitrocellulose or polyvinylidene flouride polymer (PVDF) membrane, the blot was blocked with 5% milk or 3% BSA and TBS-T solution and was subsequently incubated with anti-SR protein (MABE126 Clone 16H3, Millipore), anti-phospho SR antibody (1/1000, 1H4, Invitrogen), anti-CLK1 (N-17, sc-47959, Santa Cruz), anti-CLK2 (D-16, sc-74912, Santa Cruz), anti-CLK3 (D-10, sc-365225, Santa Cruz), anti-SRRM1 (1/250, B1C8, gift from Prof Ben Blencowe), or anti-GAPDH (loading control, 1/5000, MAB8374, EMD Millipore, USA or 1/1000, I-19, sc-48166, Santa Cruz) overnight at 4 °C. Detection with ECL Plus was carried out following washing and incubation with the respective HRP-conjugated secondary antibody and the images captured digitally on an ImageQuant LAS4000 (GE Healthcare, Chicago, IL, USA) (Supplementary Fig. 15).

### Immunoprecipitation

IP eluates were prepared for trypsin digest using SP3 protein clean-up^55^. Briefly, SP3 bead-protein mixture was washed with 50% (v/v) acetonitrile, 70% absolute ethanol, and 100% acetonitrile. Proteins were eluted into 50 µL of 50 mm HEPES pH 8 that contained Trypsin/rLysC enzyme mix (Promega) at a 1:25 ratio of enzyme to protein and incubated for 14 h at 37 °C. Peptide mixtures were labeled with TMTs (Pierce) to facilitate quantification. For each sample, 10 µg of peptide was labeled with 10 µg of TMT label per 1 µg protein,in two volumetrically equal steps, 30 min apart, quenched, pooled, concentrated, and desalted using StageTips prior to MS analysis.

### TMT mass spectrometry data acquisition

Analysis of TMT labeled peptide fractions was carried out on an Orbitrap Fusion Tribrid MS platform (Thermo Scientific) with an Easy-nLC 1000 system (Thermo Scientific). Columns used for trapping and separations were packed in-house. Elution was performed with a gradient of mobile phase A (98.9% water, 1% DMSO, and 0.1% formic acid) to 22% B (98.9% acetonitrile, 1% DMSO, and 0.1% formic acid) over 120 min, and to 40% B over 30 min, with final elution and equilibration using a further 25 min at a flow rate of 350 nL per min. Data acquisition on the Orbitrap Fusion (control software version 2.0) was carried out using a data-dependent method with multi-notch synchronous precursor selection MS3 scanning for TMT tags^56,57^. Survey scans covering the mass range of 350–1500 were acquired at a resolution of 120,000 (at m z^−1^ 200). For MS2 scan triggering, monoisotopic precursor selection was enabled, charge state filtering was limited to 2–5, an intensity threshold of 5^3^ was employed, and dynamic exclusion of previously selected masses was enabled for 45 s with a tolerance of 10 ppm. Fragment ions were selected for MS3 scans based on a precursor selection range of 400–2000 m z^−1^, ion exclusion of 20 m z^−1^ low and 5 m z^−1^ high, and isobaric tag loss exclusion for TMT. A total of 10 precursors were selected for MS3 scans that were acquired in the orbitrap after high energy collision dissociation (HCD) fragmentation.

### Mass spectrometry data acquisition and analysis

Data from the Orbitrap Fusion were processed using Proteome Discoverer Software (ver. 2.1.0.62). MS2 spectra were searched using Sequest HT against the UniProt Human proteome database appended to a list of common contaminants (20,239 total sequences). Sequest parameters were specified as: trypsin enzyme, two missed cleavages allowed, minimum peptide length of 6, precursor mass tolerance of 20 ppm, and a fragment mass tolerance of 0.8 daltons. Peptide spectral match error rates were determined using the target-decoy strategy coupled to Percolator modeling of positive and false matches^58,59^. Reporter ions were quantified from MS3 scans using an integration tolerance of 20 ppm with the most confident centroid setting. Output quantification values represented the signal-to-noise of the TMT value relative to the orbitrap preamplifier. Only unique peptides were retained for all quantification analyses. Data were transformed and normalized using the R package vsn, a process similar to that used for data from multiple gene chip experiments^60^. Normalized data were then analyzed using a blocked two-way ANOVA for each gene target (blocks represent peptide sequences and the treatments applied). An omnibus test for any treatment effect was conducted for each gene target, and resultant *p*-values adjusted for multiple comparisons using the method of Benjamini and Hochberg^48^ (BH-adjusted). BH-adjusted *p*-values were used in constructing a volcano plot. A test for treatment-associated trend was conducted only for gene targets showing a BH-adjusted *p*-value of <0.05.

### Data availability

The mass spectrometry proteomics data have been deposited to the ProteomeXchange Consortium via the PRIDE partner^61^ repository with the data set identifier PXD003839. Raw sequence reads used in this study are available at the Short Read Archive under the accession code SRP091981 and BioProject identifier PRJNA32156. All other data available from the authors upon reasonable request.

## Electronics supplementary material

Supplementary Information Supplementary Figures, Supplementary Tables, Supplementary Notes, and Supplementary References

Supplementary Data 1 Percent inhibition represents a ratio of phosphorylated product formed in the presence of the compound compared with phosphorylated product formed in reactions containing 1% DMSO. Inhibition data indicates the mean value of kinase inhibition from duplicate data sets.

Supplementary Data 2 ∆AS events from MISO for the HCT116 T3 replicate dataset. Columns: t3_concentration (T3 concentration, μM; float), sample* (sample/library id; text), event_type (MISO AS event type; text), event_name (MISO AS event name; text), bayes_factor (Bayes factor for comparison; float), diff (difference between sample1 and sample2 PSI; float), gene (gene name; text), isoforms (names of events considered; text), sample*_counts (counts for each read type; text), sample*_assigned_counts (inferred assignments of reads to isoforms; text), sample*_posterior_mean (posterior PSI estimate; float), sample*_ci_high (upper bound of 95% confidence interval for PSI; float), sample*_ci_low (lower bound of 95% confidence interval for PSI; float).

Supplementary Data 3 deFuse detected CGs in the HCT116 T3 repeat dataset. See deFuse documentation for output format: https://bitbucket.org/dranew/defuse.

Supplementary Data 4 ∆AS events from MISO from KH-CB19 treated HCT116 cells. Columns: event_type (MISO AS event type; text), event_name (MISO AS event name; text), sample*_posterior_mean (posterior PSI estimate; float), sample*_ci_low (lower bound of 95% confidence interval for PSI; float), sample*_ci_high (upper bound of 95% confidence interval for PSI; float), diff (difference between sample1 and sample2 PSI; float), bayes_factor (Bayes factor for comparison; float), isoforms (names of events considered; text), sample*_counts (counts for each read type; text), sample*_assigned_counts (inferred assignments of reads to isoforms; text), chrom (chromosome name; text), strand (chromosome strand of event; text), mRNA_starts (start coordinates of mRNA entries in event; text), mRNA_ends (end coordinates of mRNA entries in event; text), Gene (gene name of event; text), sample* (sample/library id; text), KH-CB19_concentration (T3 concentration, μM; float).

Supplementary Data 5 deFuse detected CGs in KH-CB19 treated HCT116 cells. See deFuse documentation for output format: https://bitbucket.org/dranew/defuse.

Supplementary Data 6 ∆AS events from MISO for the HCT116 and 184hTERT datasets. Columns: dataset (cell and RNA-Seq library type; text), t3_concentration (T3 concentration, μM; float), sample1 (sample1 sample/library id; text), sample2 (sample2 sample/library id; text), event_type (MISO AS event type; text), event_name (MISO AS event name; text), bayes_factor (Bayes factor for comparison; float), diff (difference between sample1 and sample2 PSI; float), gene (gene name; text), isoforms (names of events considered; text), sample*_counts (counts for each read type; text), sample*_assigned_counts (inferred assignments of reads to isoforms; text), sample*_posterior_mean (posterior PSI estimate; float), sample*_ci_high (upper bound of 95% confidence interval for PSI; float), sample*_ci_low (lower bound of 95% confidence interval for PSI; float).

Supplementary Data 7 AS event PSI values across all T3 concentrations in HCT116 and 184hTERT datasets. Columns: event_type (event type name; text), event_name (event identifier; text), t3_concentration (T3 concentration, μM; float), psi (percent spliced in value; float), dataset (cell and RNA-Seq library type; text).

Supplementary Data 8 ∆AS events from VAST-TOOLS for the HCT116 and 184hTERT datasets. Columns: GENE (gene name; text), EVENT (AS event name; text), COORD (event coordinates; text), LENGTH (event length; int), FullCO (full event coordinates; text), COMPLEX (AS event type; text), sample*_psi (event PSI; float), sample*_scores (quality scores; text), sample* (sample/library id; text), dataset (cell and RNA-Seq library type; text), sample2_t3_concentration (T3 concentration, μM; float).

Supplementary Data 9 VAST-TOOLS AS event PSI values across all T3 concentrations in HCT116 and 184hTERT datasets. Columns: EVENT (event identifier; text), COMPLEX (event type; text), PSI (percent spliced in value; float), LIBRARY (library/sample id; text), dataset (cell and RNA-Seq library type; text).

Supplementary Data 10 ∆DI events from MISO in the HCT116 and 184hTERT datasets. Columns: event_name (MISO AS event name; text), event_type (MISO AS event type; text), sample*_posterior_mean (posterior PSI estimate; float), sample*_ci_high (upper bound of 95% confidence interval for PSI; float), sample*_ci_low (lower bound of 95% confidence interval for PSI; float), diff (difference between sample1 and sample2 PSI; float), bayes_factor (Bayes factor for comparison; float), isoforms (names of events considered; text), sample*_counts (counts for each read type; text), sample*_assigned_counts (inferred assignments of reads to isoforms; text), chrom (chromosome name; text), strand (chromosome strand of event; text), mRNA_starts (start coordinates of mRNA entries in event; text), mRNA_ends (end coordinates of mRNA entries in event; text), dataset (cell and RNA-Seq library type; text), t3_concentration (T3 concentration, μM; float), sample* (sample/library id; text).

Supplementary Data 11 DI PSI values across all T3 concentrations in HCT116 and 184hTERT datasets. Columns: event_type (event type name; text), event_name (event identifier; text), t3_concentration (T3 concentration, μM; float), psi (percent spliced in value; float), dataset (cell and RNA-Seq library type; text).

Supplementary Data 12 ∆AS events from MISO for the HCT116 CLK siRNA dataset. Columns: event_name (MISO AS event name; text), event_type (MISO AS event type; text), sample*_posterior_mean (posterior PSI estimate; float), sample*_ci_high (upper bound of 95% confidence interval for PSI; float), sample*_ci_low (lower bound of 95% confidence interval for PSI; float), diff (difference between sample1 and sample2 PSI; float), bayes_factor (Bayes factor for comparison; float), isoforms (names of events considered; text), sample*_counts (counts for each read type; text), sample*_assigned_counts (inferred assignments of reads to isoforms; text), chrom (chromosome name; text), mRNA_starts (start coordinates of mRNA entries in event; text), mRNA_ends (end coordinates of mRNA entries in event; text), treatment (siRNA treatment; text), sample* (sample/library id; text).

Supplementary Data 13 Enriched GO BP terms for genes affected by MISO ∆AS events shared between the T3-treated and CLK RNAi datasets. Columns: topic (GO BP term; text), hit.num (# input genes topic; int), number.in.topic (# annotated genes in topic; int), ratio.of.topic (ratio of # genes in topic to total genes in Reactome FI network; float), p.value (P-value; float), fdr (Benjamini-Hochberg corrected P-values; float), hits (input genes found in gene set; text).

Supplementary Data 14 Enriched GO BP terms for genes affected by MISO ∆AS events at lower (0.05– Samuel AJR Aparicio BM BCh PhD FRCPath BC Cancer Research Centre Nan and Lorraine Robertson Chair of Breast Cancer Research, UBC/BCCA 675 West 10th Avenue CRC Chair, Molecular Oncology Vancouver, BC saparicio@bccrc.ca V5Z 1L3, Canada +1 604 675 8201(T) +1 604 675 8214 (fax) 0.5 μM) or higher (1.0–10.0 μM) concentrations in the HCT116 and 184hTERT datasets. Columns: topic (GO BP term; text), hit.num (# input genes topic; int), number.in.topic (# annotated genes in topic; int), ratio.of.topic (ratio of # genes in topic to total genes in Reactome FI network; float), p.value (P-value; float), fdr (Benjamini-Hochberg corrected P-values; float), hits (input genes found in gene set; text), dataset (cell and RNA-Seq library type; text), t3_concentration_range (T3 concentration range, μM; text).

Supplementary Data 15 RBP motif clusters for ∆AS PSI motif density heatmaps. RBP clusters for both full and condensed heatmaps are included. Columns: event_type (MISO AS event type; text), event_name (MISO AS event name; text), cluster (PSI response cluster; int), dataset (cell and RNA-Seq library type; text).

Supplementary Data 16 Hypergeometric test P-values for the enrichment of AS event types within AS PSI clusters. P-values have been multiple-test corrected using the Benjamini-Hochberg procedure. Columns: cluster (PSI response cluster; int), A3SS–SE (enrichment P-values; float), dataset (cell and RNA-Seq library type; text).

Supplementary Data 17 FPKM values for genes in the HCT116 and 184hTERT datasets. Columns: gene (gene name; text), library (library/sample id; text), fpkm (gene expression; float), dataset (cell and RNASeq library type; text), t3_concentration (T3 concentration, μM; float).

Supplementary Data 18 Gene expression profile clusters for the HCT116 and 184hTERT datasets. Columns: gene (gene name; text), cluster (FPKM response cluster; int), dataset (cell and RNA-Seq library type; text).

Supplementary Data 19 Enriched GO BP terms for FPKM gene clusters in the HCT116 and 184hTERT datasets. Columns: topic (GO BP term; text), hit.num (# input genes topic; int), number.in.topic (# annotated genes in topic; int), ratio.of.topic (ratio of # genes in topic to total genes in Reactome FI network; float), p.value (P-value; float), fdr (Benjamini-Hochberg corrected P-values; float), hits (input genes found in gene set; text), cluster (FPKM gene cluster; int), dataset (cell and RNA-Seq library type; text).

Supplementary Data 20 RBP motif clusters for ∆AS PSI motif density heatmaps. RBP clusters for both full and condensed heatmaps are included. Columns (some info from Ray et al. [4] and Gerstberger et al. [5]): dend_cl (dendrogram cluster; int), motif_id (motif id; text), gene_id (Ensembl gene id; text), rna_binding_domains (RNA binding protein domains in gene; text), gene_name (gene name; text), gene_desc (gene description; text), domains (protein domains in gene; text), paralogous_family (Gerstberger, et al. RNA binding protein family; text), consensus_rna_target (RNA binding target; text), go_rna (RNA related GO terms for the gene; text), go (GO terms for the gene; text), psi_cluster (PSI response cluster; int), heatmap_type (full or condensed heatmap; text).

Supplementary Data 21 deFuse detected CGs in the HCT116 and 184hTERT datasets. See deFuse documentation for output format: https://bitbucket.org/dranew/defuse.

Supplementary Data 22 deFuse detected CGs in the CLK siRNA datasets. See deFuse documentation for output format: https://bitbucket.org/dranew/defuse.

Supplementary Data 23 CGs detected in the targeted sequencing dataset. Columns: cg (CG name as upstream-gene@splicesite:downstream-gene@splicesite; text), validation_input (‘Y’ if CG was selected for validation; text), other columns (‘Y’ if CG was detected in this sample/library).

Supplementary Data 24 CG-supporting PacBio read counts in the HCT116 unstranded, stranded and 184hTERT datasets. Columns: chrom (chromosome name; text), strand (genomic strand; text), up_pos (upstream CG junction position; int), dn_pos (downstream CG junction position; int), pacbio_reads (the number of PacBio reads supporting the CG; int), dataset (cell and RNA-Seq library type; text).

Supplementary Data 25 Normalized expression values for CGs detected in the CG validation targeted sequencing experiment. Columns: sample_id (sample id; text), chrom (chromosome; text), strand (genomic strand; text), gene* (CG partner name; text), breakpt* (CG splice junction position; int), *_norm (CG expression, normalized by expression of named gene; float).

Supplementary Data 26 Enriched GO BP terms for upstream and downstream CG participants in the HCT116 and 184hTERT datasets. Columns: topic (GO BP term; text), hit.num (# input genes topic; int), number.in.topic (# annotated genes in topic; int), ratio.of.topic (ratio of # genes in topic to total genes in Reactome FI network; float), p.value (P-value; float), fdr (Benjamini-Hochberg corrected P-values; float), hits (input genes found in gene set; text), dataset (cell and RNA-Seq library type; text), participant (upstream or downstream CG participant; text).

Supplementary Data 27 CG event counts with particular splicing patterns for the HCT116 and 184hTERT datasets. Splicing patterns are indicated by the number of upstream and downstream partner gene exons skipped in the CG event. Columns: up_skipped (# exons skipped in upstream CG partner; int), dn_skipped (# exons skipped in downstream CG partner; int), count (CG count; int), fraction (fraction of CGs in dataset; float), dataset (cell and RNA-Seq library type; text).

Supplementary Data 28 CG candidate events from PacBio libraries. T3 concentration is included in the “Note” field of the attribute column.

Supplementary Data 29 ∆CG events from MISO in the HCT116 and 184hTERT datasets. Columns: event_name (MISO AS event name; text), event_type (MISO AS event type; text), sample*_posterior_mean (posterior PSI estimate; float), sample*_ci_low (lower bound of 95% confidence interval for PSI; float), sample*_ci_high (upper bound of 95% confidence interval for PSI; float), diff (difference between sample1 and sample2 PSI; float), bayes_factor (Bayes factor for comparison; float), isoforms (names of events considered; text), sample*_counts (counts for each read type; text), sample*_assigned_counts (inferred assignments of reads to isoforms; text), chrom (chromosome name; text), strand (chromosome strand of event; text), mRNA_starts (start coordinates of mRNA entries in event; text), mRNA_ends (end coordinates of mRNA entries in event; text), dataset (cell and RNA-Seq library type; text), t3_concentration (T3 concentration, μM; float), sample* (sample/library id; text).

Supplementary Data 30 CG PSI values across all T3 concentrations in HCT116 and 184hTERT datasets. Columns: event_type (event type name; text), event_name (event identifier; text), t3_concentration (T3 concentration, μM; float), psi (percent spliced in value; float), dataset (cell and RNA-Seq library type; text).

Supplementary Data 31 RBP motif clusters for CG motif density heatmaps. Both full and condensed heatmap clusters are included. Columns (some info from Ray et al. and Gerstberger et al.): dend_cl (dendrogram cluster; int), motif_id (motif id; text), gene_id (Ensembl gene id; text), rna_binding_domains (RNA binding protein domains in gene; text), gene_name (gene name; text), gene_desc (gene description; text), domains (protein domains in gene; text), paralogous_family (Gerstberger, et al. RNA binding protein family; text), consensus_rna_target (RNA binding target; text), go_rna (RNA related GO terms for the gene; text), go (GO terms for the gene; text), heatmap_type (full or condensed heatmap; text).

Supplementary Data 32 CLK2 associated factors identified by IP-MS. Table of the factors associated with CLK2 as identified by IP-MS experiment by Gene name, Accession ID and unique peptide sequences summarized by gene level. Also shown are the log2 average treatment effect difference from control and the Benjamini-Hochberg adjusted Wald P-value for average treatment effect, which were used to generate the volcano plot. Genes encoding factors whose motif density are altered in CGs on T3 treatment are annotated, as are the previously identified interactors of CLK2 downloaded from three public online databases (Uniprot [6], BioGrid [7] and IntAct [8]). Those genes encoding factors annotated to be involved in RNA splicing and/or RNA binding and/or RNA 3′ -end processing from GO [3] are also indicated on the sheet.

Supplementary Data 33 CGs detected from siRNA knockdowns of splicing factors. See deFuse documentation for output format: https://bitbucket.org/dranew/defuse.

Supplementary Data 34 Normalized expression values for CGs detected from siRNA knockdowns of SRRM1/2. Columns: sample_id (sample id; text), chrom (chromosome; text), strand (genomic strand; text), gene* (CG partner name; text), breakpt* (CG splice junction position; int), *_norm (CG expression, normalized by expression of named gene; float).

Supplementary Data 35 Primers to assess expression of conjoined genes. Table of primers and the genomic locations of the respective amplicons (hg19) used to assess the expression of the various conjoined genes. Also listed length of the amplicon (on cDNA transcripts - Amplicon length) and the length between the genomic coordinates (un-spliced length).

Supplementary Data 36 Confirmation of knock down of individual factors with respective siRNAs. Table of the siRNAs used, their corresponding sequences and the effect on the respective target genes as assessed by qPCR and reported by relative expression (RQ) when compared to si-non-targeting (siNT) controls.

Supplementary Data 37 ChemDraw file for T3.

Supplementary Data 38 ChemDraw file for T3 synthetic route.

Peer Review File Reviewer reports and authors' response from the peer review of this Article at Nature Communications.
