# Supplementary material for: CLK-dependent exon recognition and conjoined gene formation revealed with a novel small molecule inhibitor
Source: Nat Commun. 2017 Feb 23;8:7. doi: 10.1038/s41467-016-0008-7 (PMC5431906; doi:10.1038/s41467-016-0008-7)
Supplement: Supplementary file 1 — Supplementary Figures, Supplementary Tables, Supplementary Notes, and Supplementary References [file 41467_2016_8_MOESM1_ESM.pdf]

a

T3 synthesis route intermediates

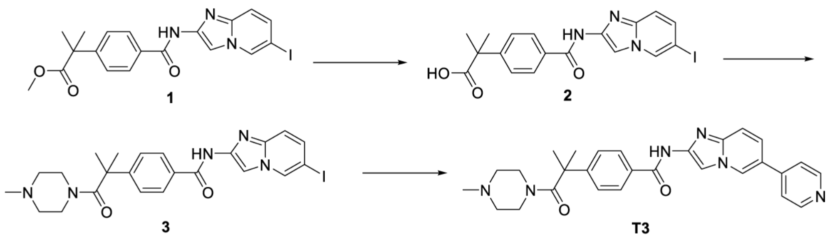

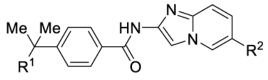

b

Compound No.	R^1	R^2	IC_{50} (nM)
1	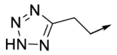	CF_3	360
2	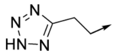	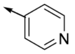	50
3	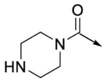	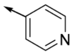	150
T3	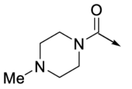	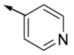	15
4	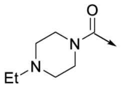	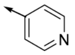	15

c

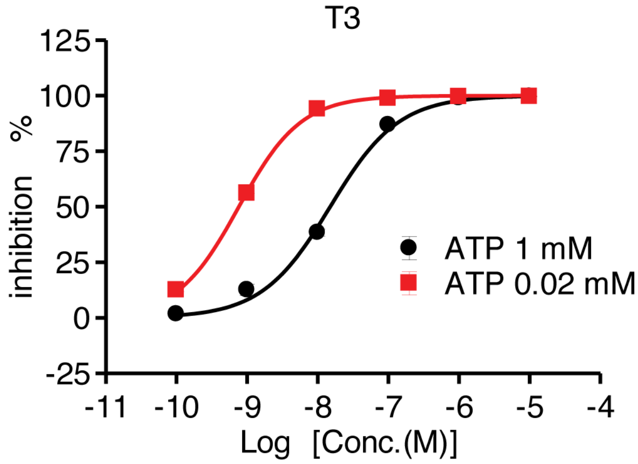

Supplementary Figure 1: Synthetic intermediate and structure-activity relationships (SAR) leading to ATP competitive inhibitor T3. **a** Structural schematic showing the main intermediates used to generate the compound T3. **b** The substitutions of the scaffold compound-1 identified by HTS and described in Araki *et al.* 2015 [1], are shown, with the IC_{50} for each compound. **c** Inhibition curves of T3 for CLK2 enzyme are shown under different ATP concentrations. IC_{50} values were assessed under 20 µM ATP with closed red squares or 1000 µM ATP with closed black circles. Curve fittings and calculations of IC_{50} value were performed with the program XLfit version 5.

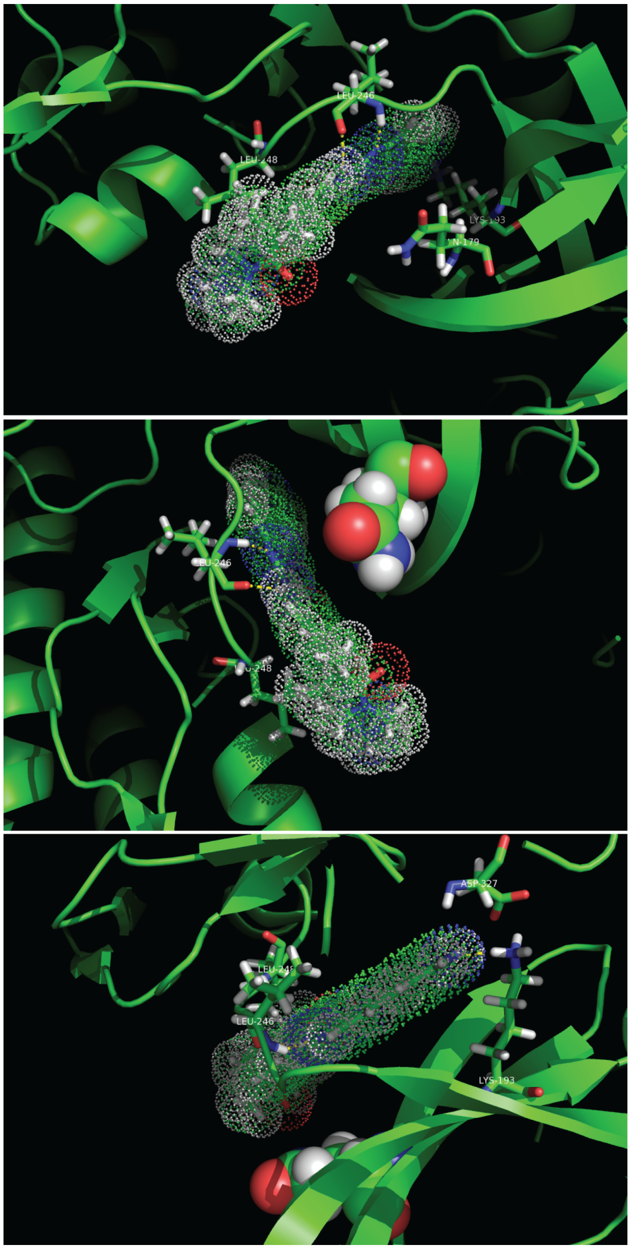

Supplementary Figure 2: Model fitted structure of CLK with docking of T3. Three views are shown to display the molecular contacts for T3 in the CLK structure

a

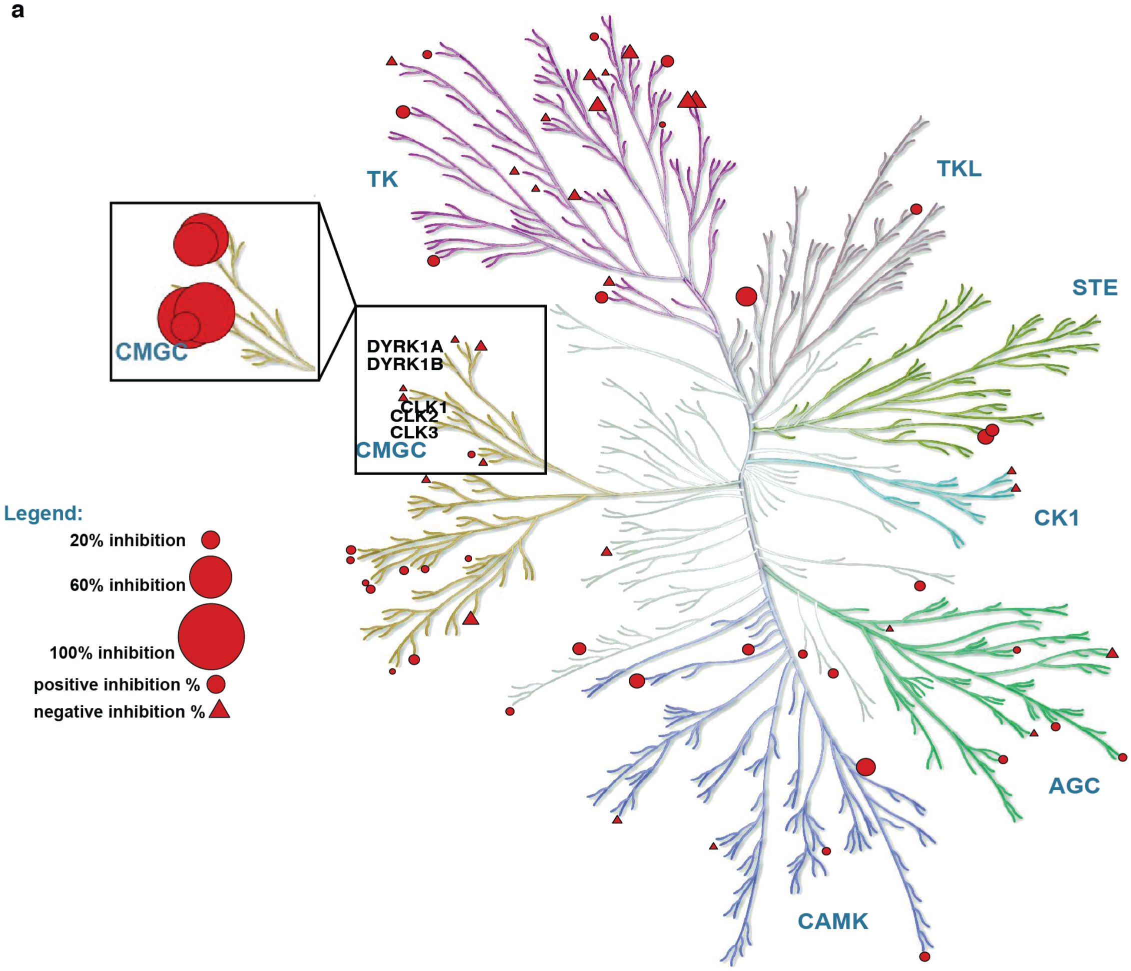

b

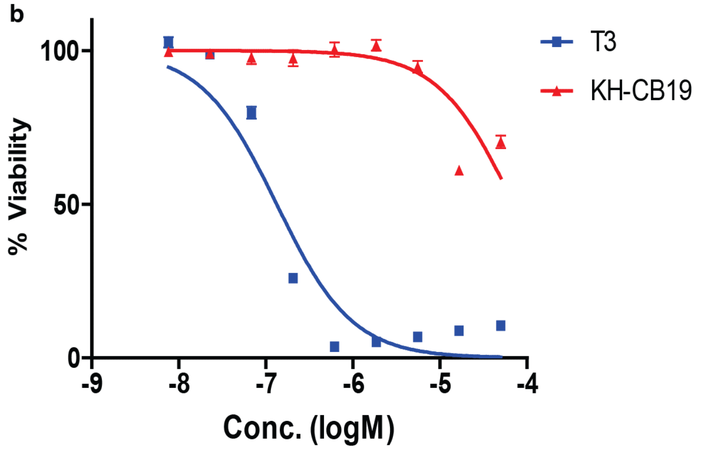

Supplementary Figure 3: Cellular activity and kinase profile of T3. Figure legend on next page

Supplementary Figure 3: Cellular activity and kinase profile of T3. **a** Selectivity of T3 *in vitro*, across a 71 kinase panel. Size of circles corresponds to the degree of inhibition (see legend). Kinome tree illustration reproduced courtesy of Cell Signaling Technology, Inc. (www.cellsignal.com). **b** Cell viablility assay comparison between T3 and KH–CB19 in HCT116 shows a GI value of 0.133 µM with T3 while it was >50 µM with KH–CB19. Error bars represent s.e.m.

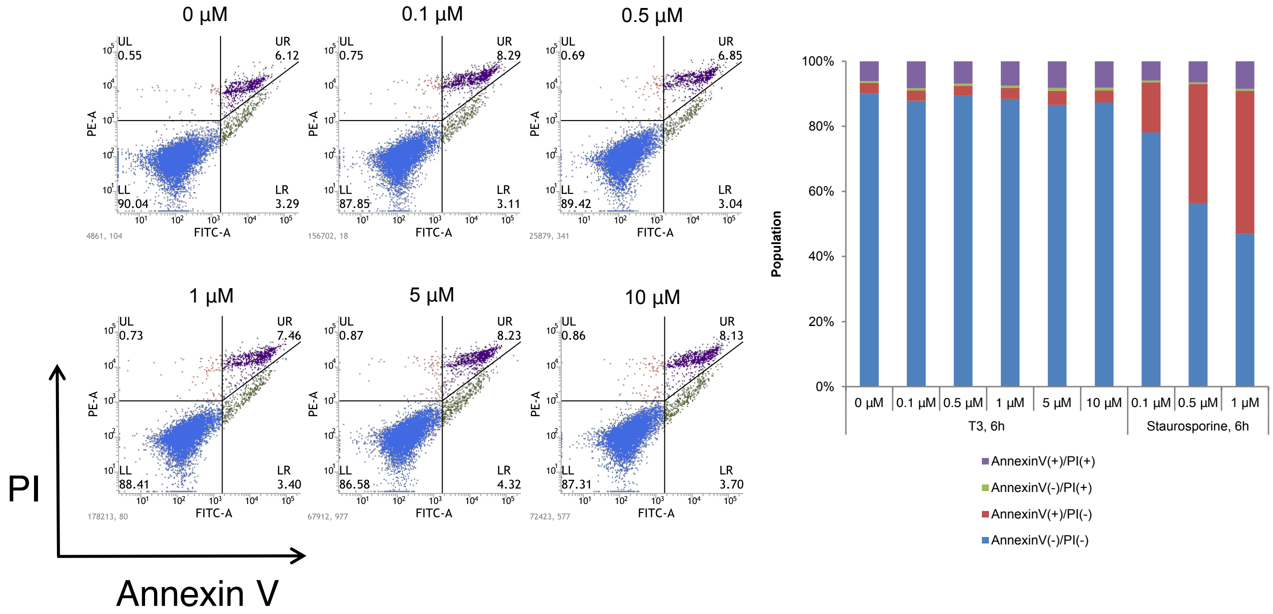

Supplementary Figure 4: Annexin V/PI timecourse with T3 and control (staurosporine)

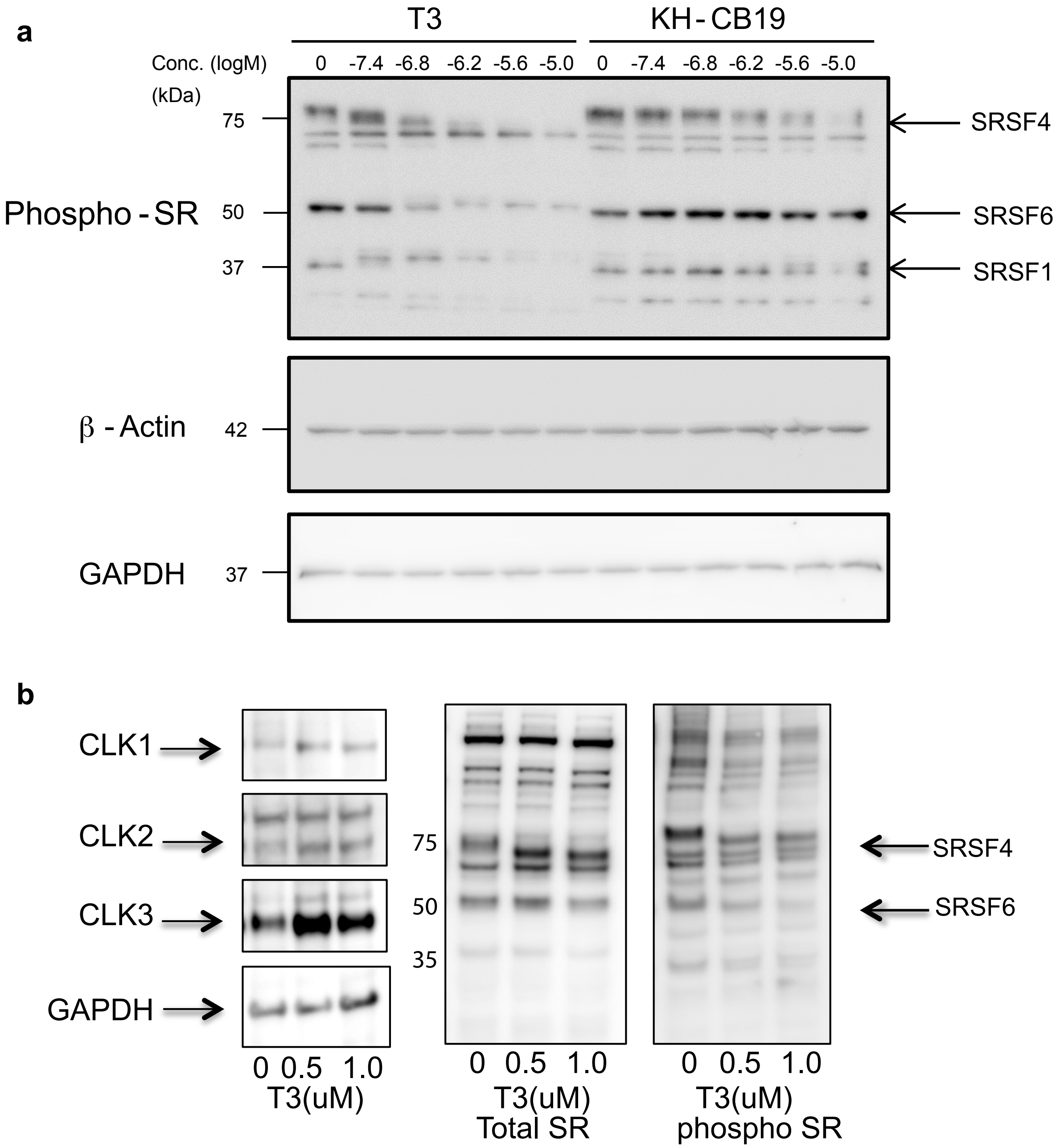

Supplementary Figure 5: Effect of CLKi on phosphorylation of SR and CLK proteins. **a** Immunoblot of phospho-SR isoforms (arrowed) in MDA-MB-468 cells treated with T3 or KH–CB19 for 3 hr. The positions of standard molecular weight markers are indicated on the left. The data shown are representative of two independent experiments. **b** HCT-116 was treated for 6 hr with T3 and immnoblotted for CLKs, total SR and phospho-SR proteins. The uncropped blots can be found in Supplementary Fig. 15.

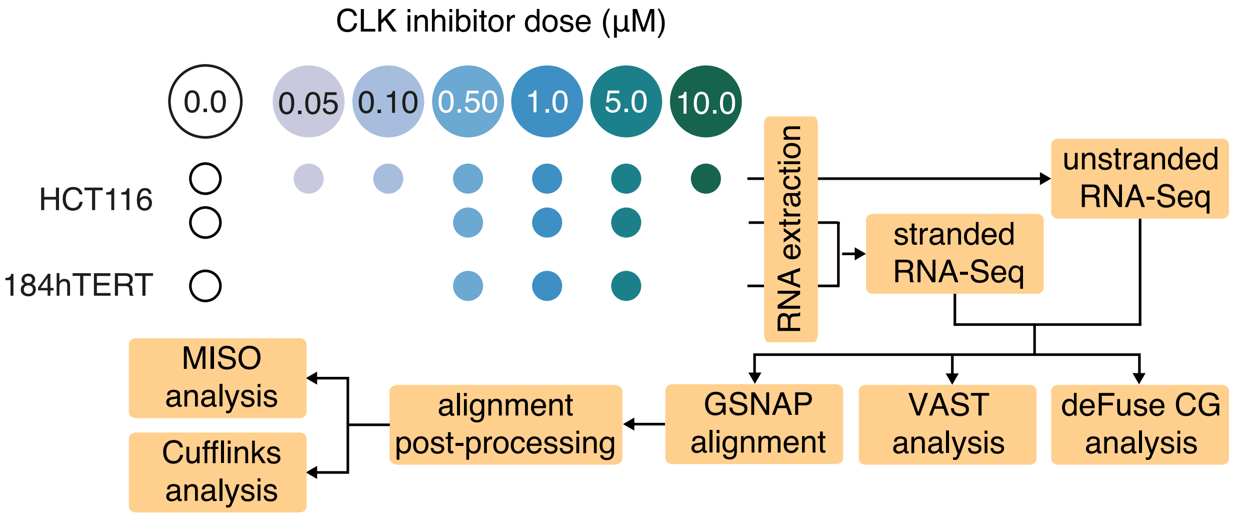

Supplementary Figure 6: Workflow diagram for analysis of T3 concentration gradient RNA-Seq datasets.

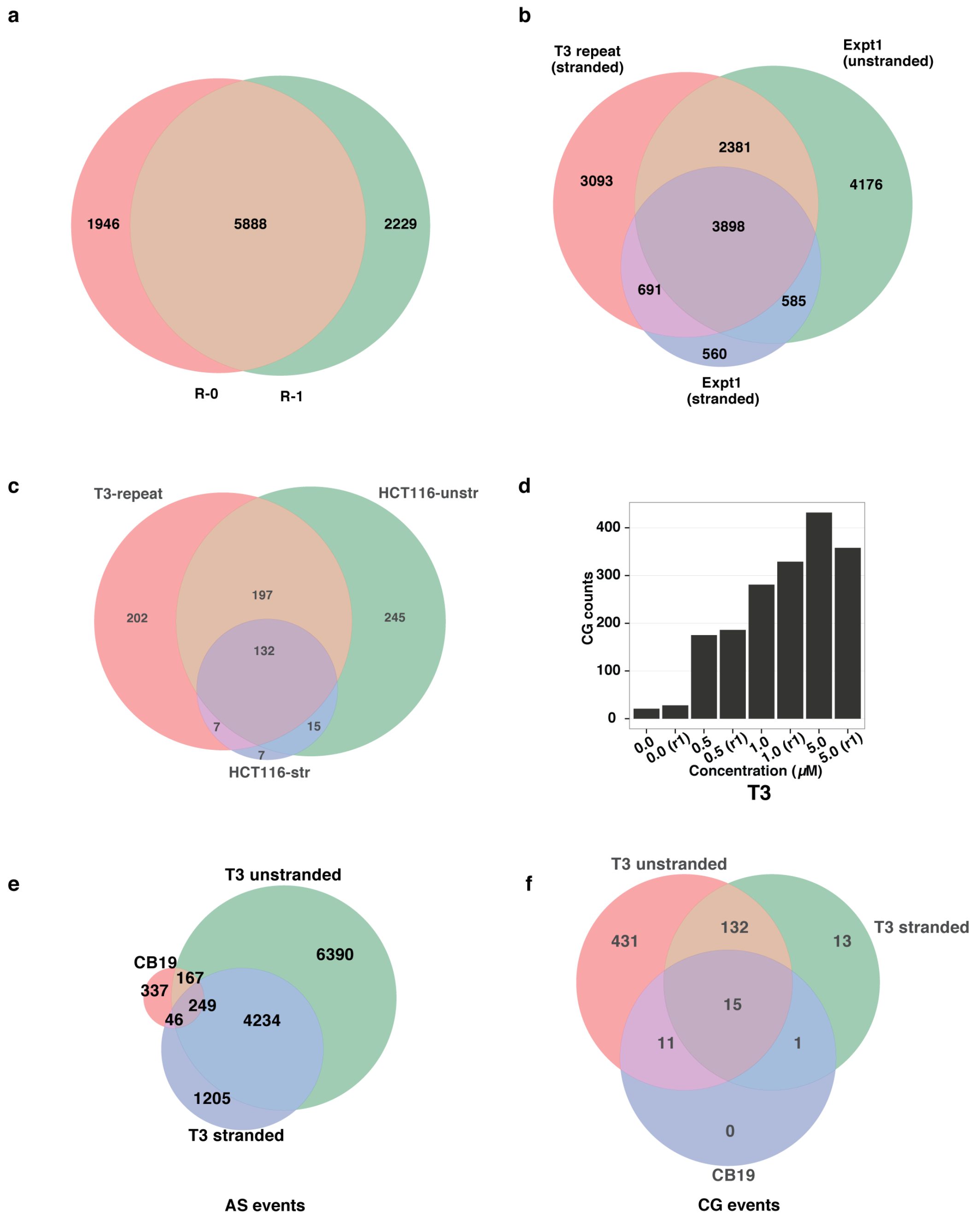

Supplementary Figure 7: Replication of RNA-seq, technical, biological and with KHCB19. **a** Total AS event overlap (MISO) between technical replicates of RNA library construction. **b** AS events overlapping between biological replicates of T3 inhibition and of stranded and unstranded libraries. **c** Conjoined gene participant pair overlap between the HCT116 unstranded and stranded RNA-Seq datasets, and the T3 concentration curve repeat experiment dataset. **d** Conjoined genes detected by deFuse in the T3 concentration curve repeat experiment. **e** Overlap between KH–CB19 AS events and T3 AS event (stranded, unstranded). **f** Overlap between KH–CB19 and T3 conjoined gene participant pairs.

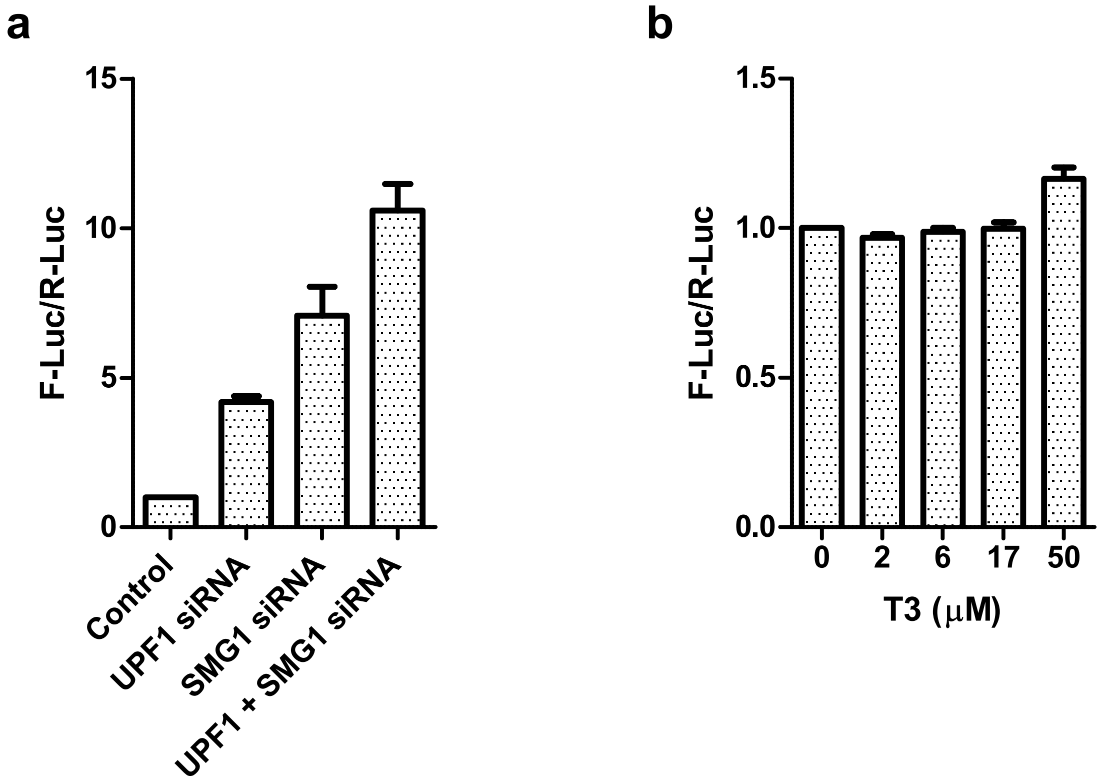

Supplementary Figure 8: NMD assay performed with T3. **a** HeLa cells transfected with siRNA against *UPF1* and/or *SMG1*, positive regulators of NMD, show an increase in NMD activity measured by Luciferase reporter assay compared to non-targeting control. **b** The relative F-Luc/R-Luc ratio in HeLa cells treated with increasing amounts of T3 for 6 h shows the relative NMD activity. The data represent means ± SD from three independent analyses.

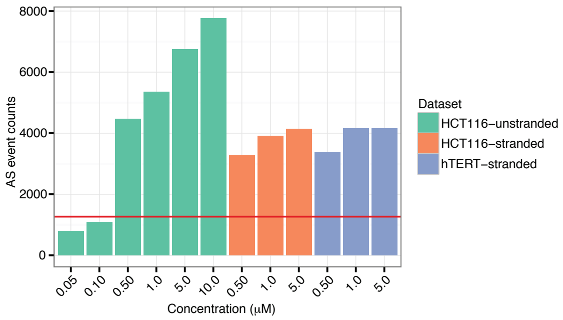

Supplementary Figure 9: Differentially spliced AS event counts for the HCT116 and 184hTERT datasets. Events have a Bayes-factor ≥ 20. Red line denotes the background baseline of 1267 AS events disjoint between HCT116 and 184hTERT in the non-treated state, determined by MISO.

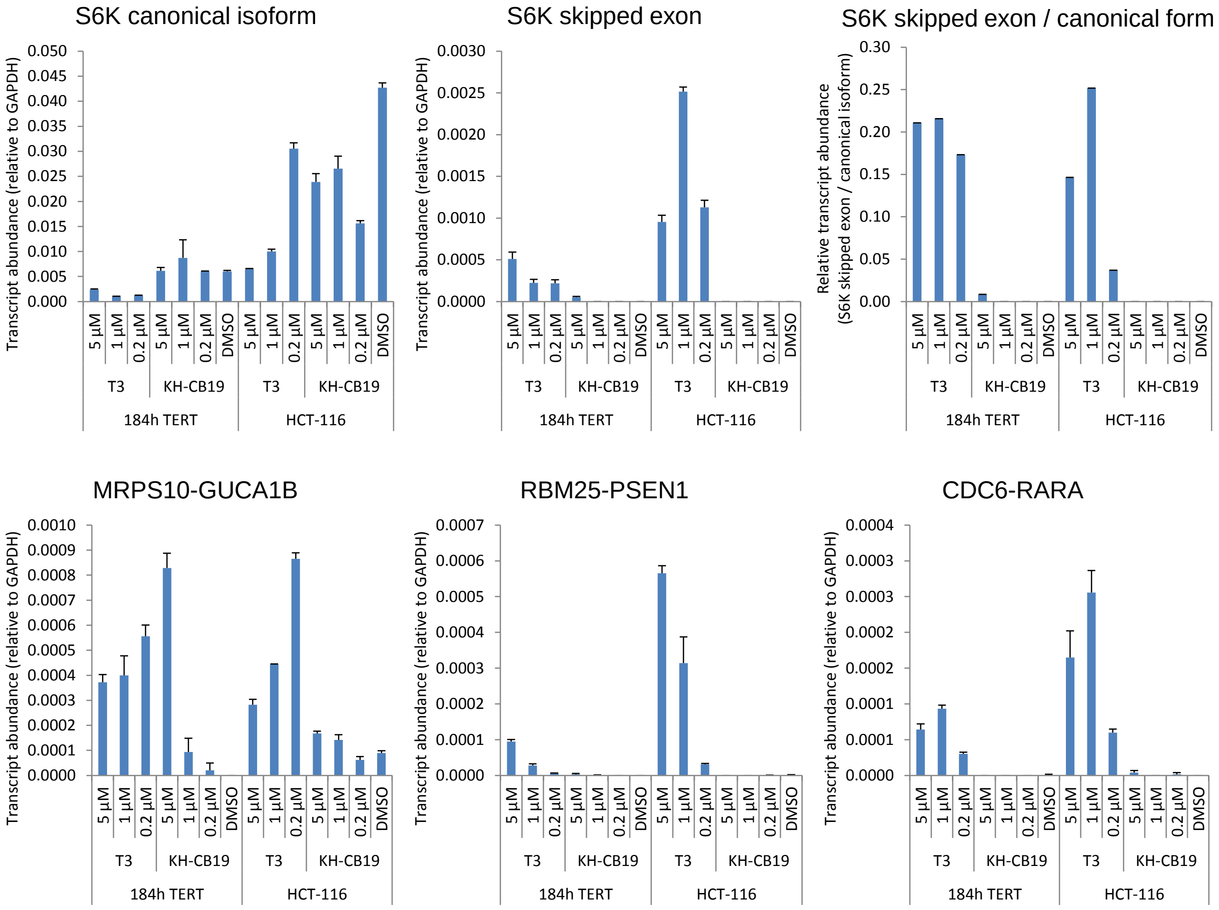

Supplementary Figure 10: HCT116 and 184hTERT cells were treated with T3 or KH–CB19 for 6 h at the indicated concentrations. Quantitative RT–PCR analyses were performed for the expression of *S6K* canonical isoform mRNA (exons 6–7), exon 7 skipped isoform (exons 6–8), and conjoined genes as shown in the figure. The data represents means ± SD from three independent analyses.

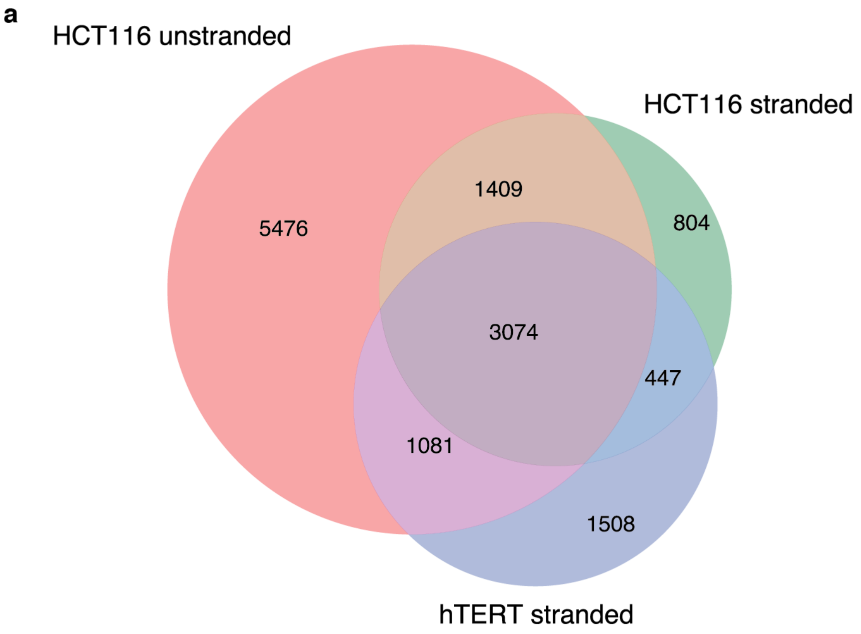

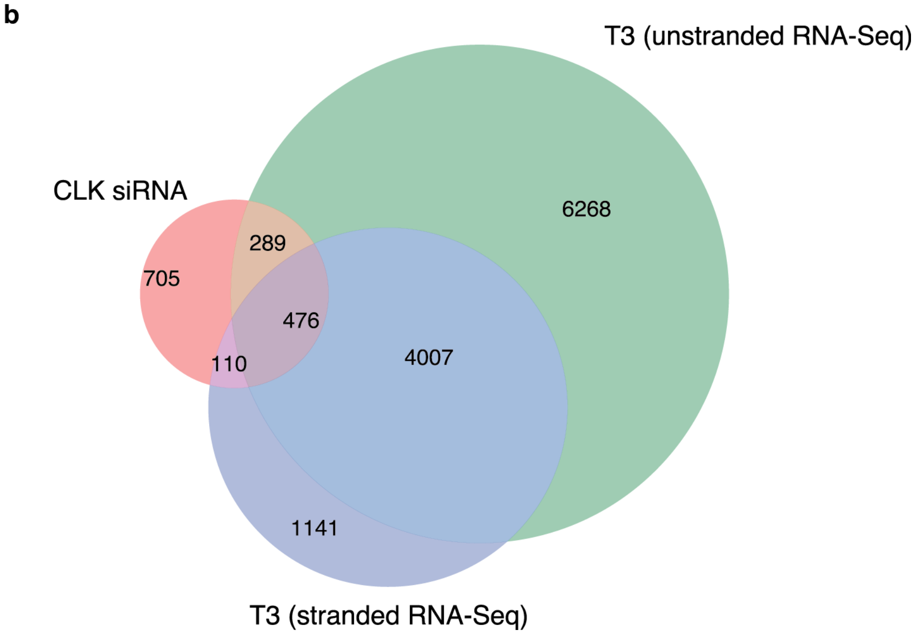

Supplementary Figure 11: Venn diagrams illustrating the number of unique overlapping and dataset-specific differentially spliced MISO events. **a** Comparison between the T3-treated HCT116 and 184hTERT RNA-Seq datasets, numbers indicate the number of overlapping ΔAS MISO events between each library. **b** Comparison between *CLK* RNAi and T3-treated HCT116 datasets, showing the number of dataset-specific and common ΔAS MISO events.

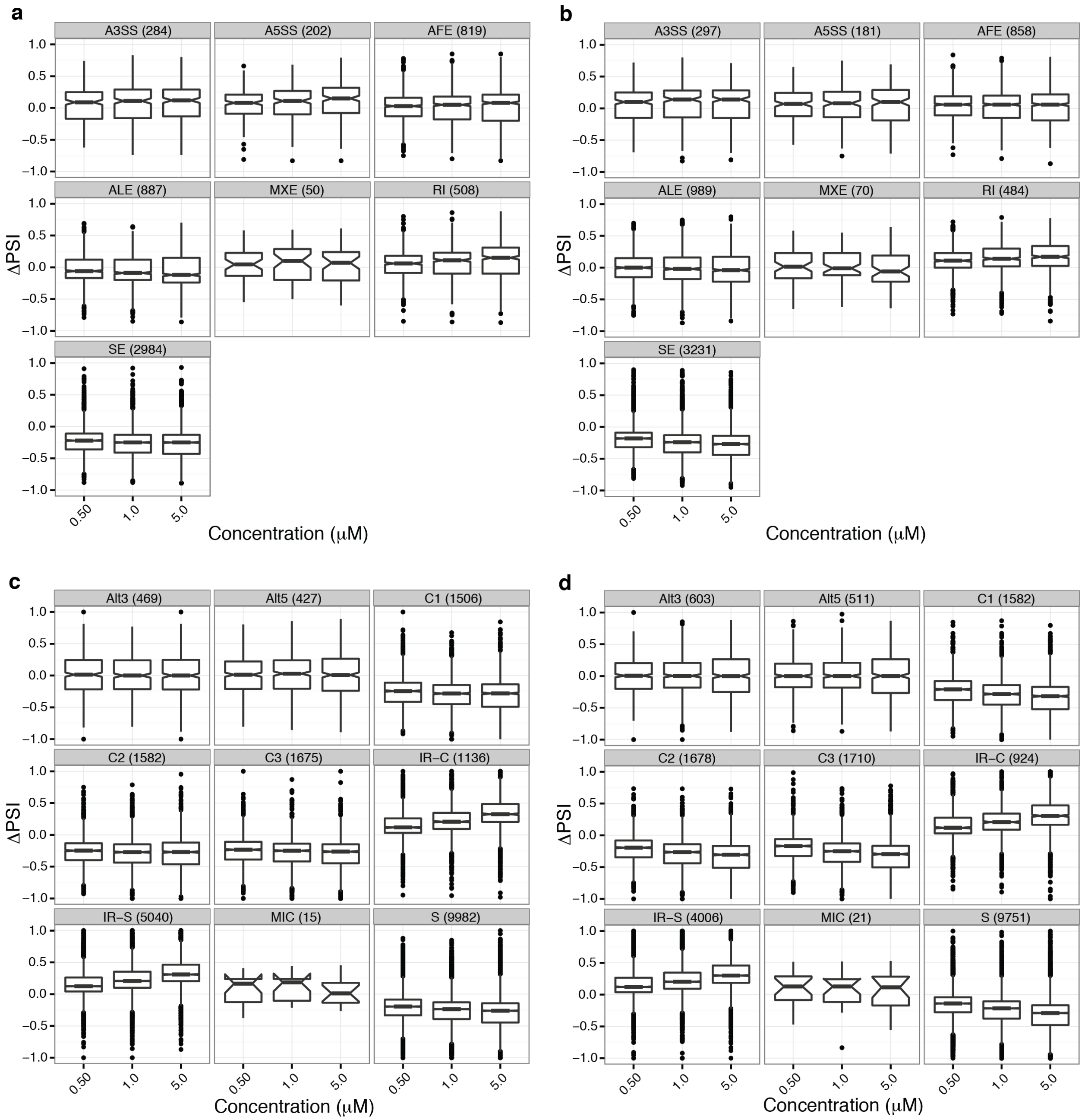

Supplementary Figure 12: ΔAS event type ΔPSI (*vs.* control) distributions across T3 CLK inhibitor concentrations. MISO ΔPSI distributions for the **a** HCT116 stranded and **b** 184hTERT RNA-Seq datasets. Event types as for MISO, see main text. VAST-TOOLS ΔAS distributions for the **c** HCT116 stranded and **d** 184hTERT RNA-Seq datasets. S, C1, C2, C3: skipped exons with increasing complexity, MIC: skipped microexons, IR-S: retained introns not overlapping another annotated event, IR-C: retained introns overlapping another annotated event, Alt3: alternative acceptor site, Alt5: alternative donor site.

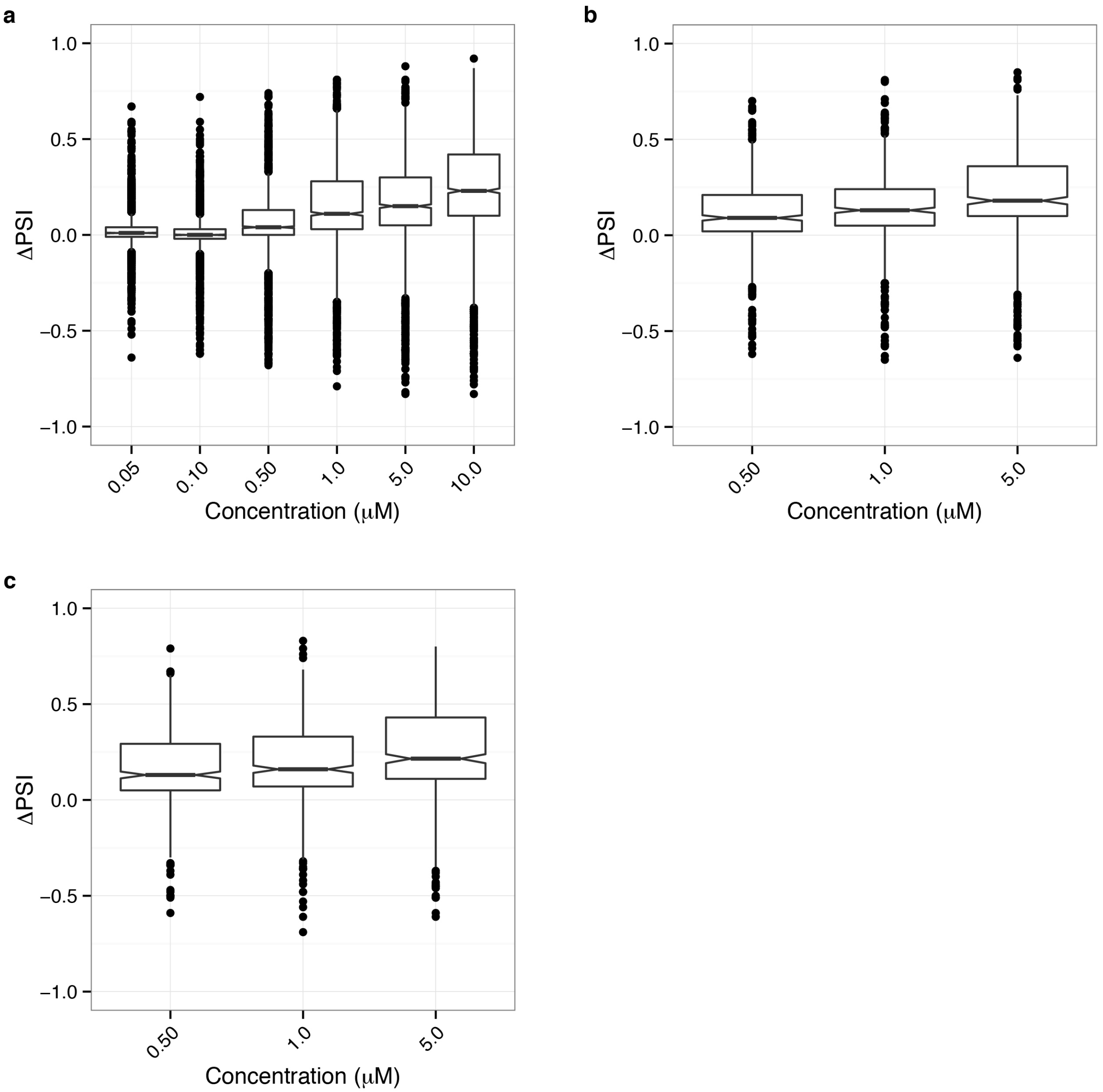

Supplementary Figure 13: Detained intron (DI) event ΔPSI boxplots across T3 concentrations. **a** HCT116 unstranded RNA-Seq dataset. **b** HCT116 stranded RNA-Seq dataset. **c** 184hTERT RNA-Seq dataset.

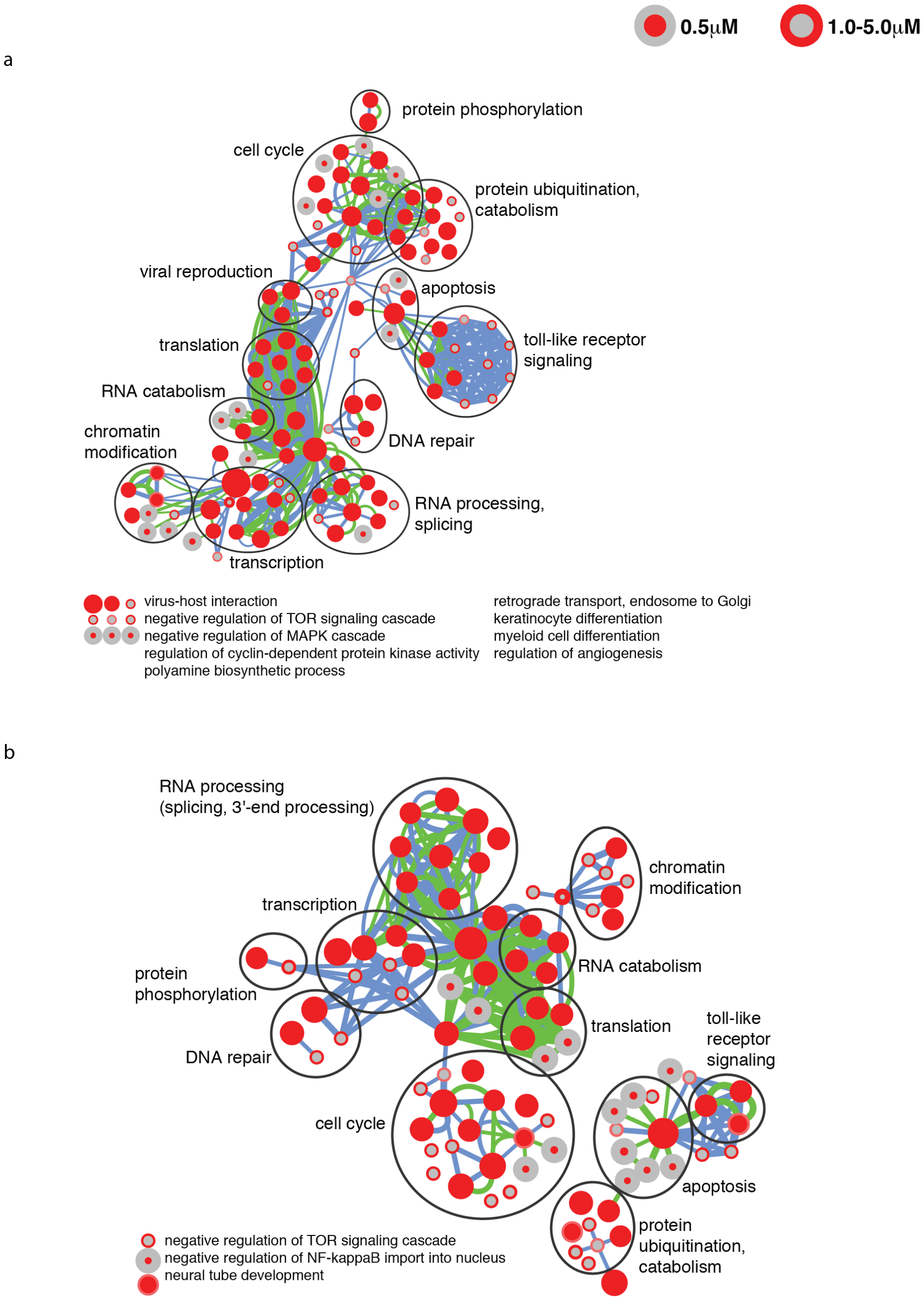

Supplementary Figure 14: Biological process enrichment maps calculated with the Cytoscape enrichment map tool. Biological process enrichment map for differentially spliced genes in the **a** HCT116 stranded and **b** 184hTERT stranded RNA-Seq datasets. Each node represents a GO biological process gene set. Node cores are coloured red when that gene set is enriched among genes differentially spliced in the 0.05 µM sample, and the outer ring is coloured red when enriched in the 1.0–5.0 µM samples. Edge thickness indicates the level of overlap between two gene sets, considering the set of differentially spliced genes in the 0.05 µM (green edges) or 1.0–5.0 µM (blue edges) samples.

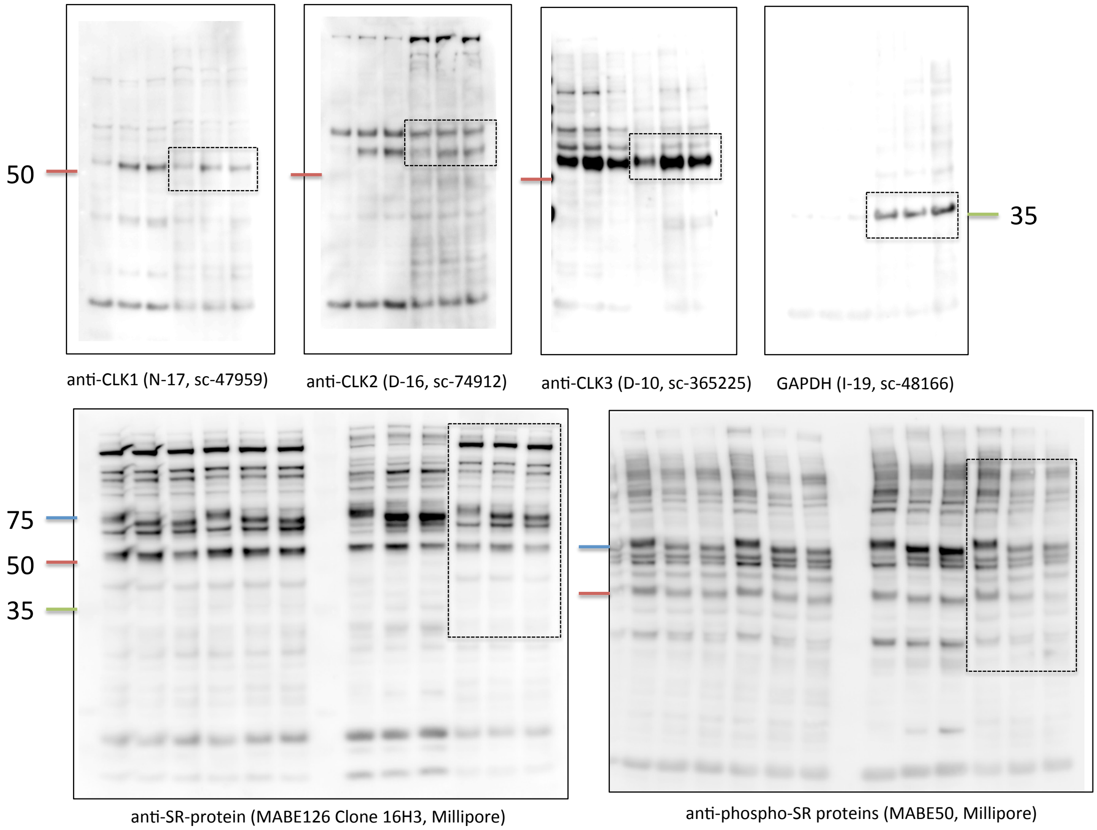

Supplementary Figure 15: Relevant uncropped images of blots in Supplementary Fig. 5

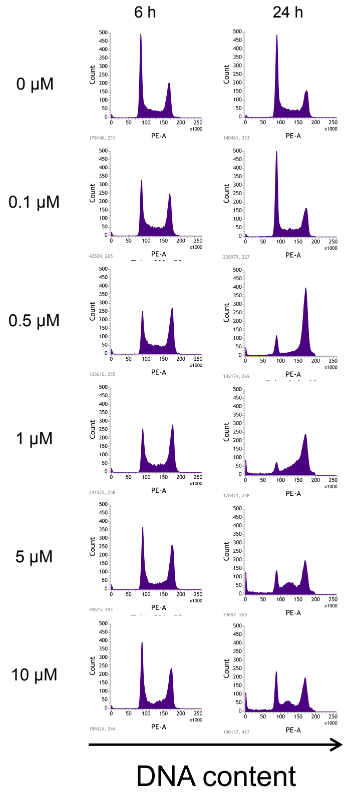

	6 h					
	0 μM	0.1 μM	0.5 μM	1 μM	5 μM	10 μM
sub G0/G1	1.6	1.5	1.6	1.5	2.2	1.7
G0/G1	35.0	24.0	18.4	18.4	28.1	28.4
G2/M	25.8	30.5	34.8	34.6	34.5	26.3
S	39.3	45.5	46.9	47.0	37.4	45.3

	24 h					
	0 μM	0.1 μM	0.5 μM	1 μM	5 μM	10 μM
sub G0/G1	2.5	2.8	13.1	20.4	20.9	19.0
G0/G1	35.7	38.7	11.4	7.3	13.3	21.3
G2/M	17.9	20.7	68.1	46.0	41.2	37.7
S	46.4	40.7	20.5	46.7	45.5	41.0

Supplementary Figure 16: Flow cytometry estimation of cell cycle state with T3 exposure

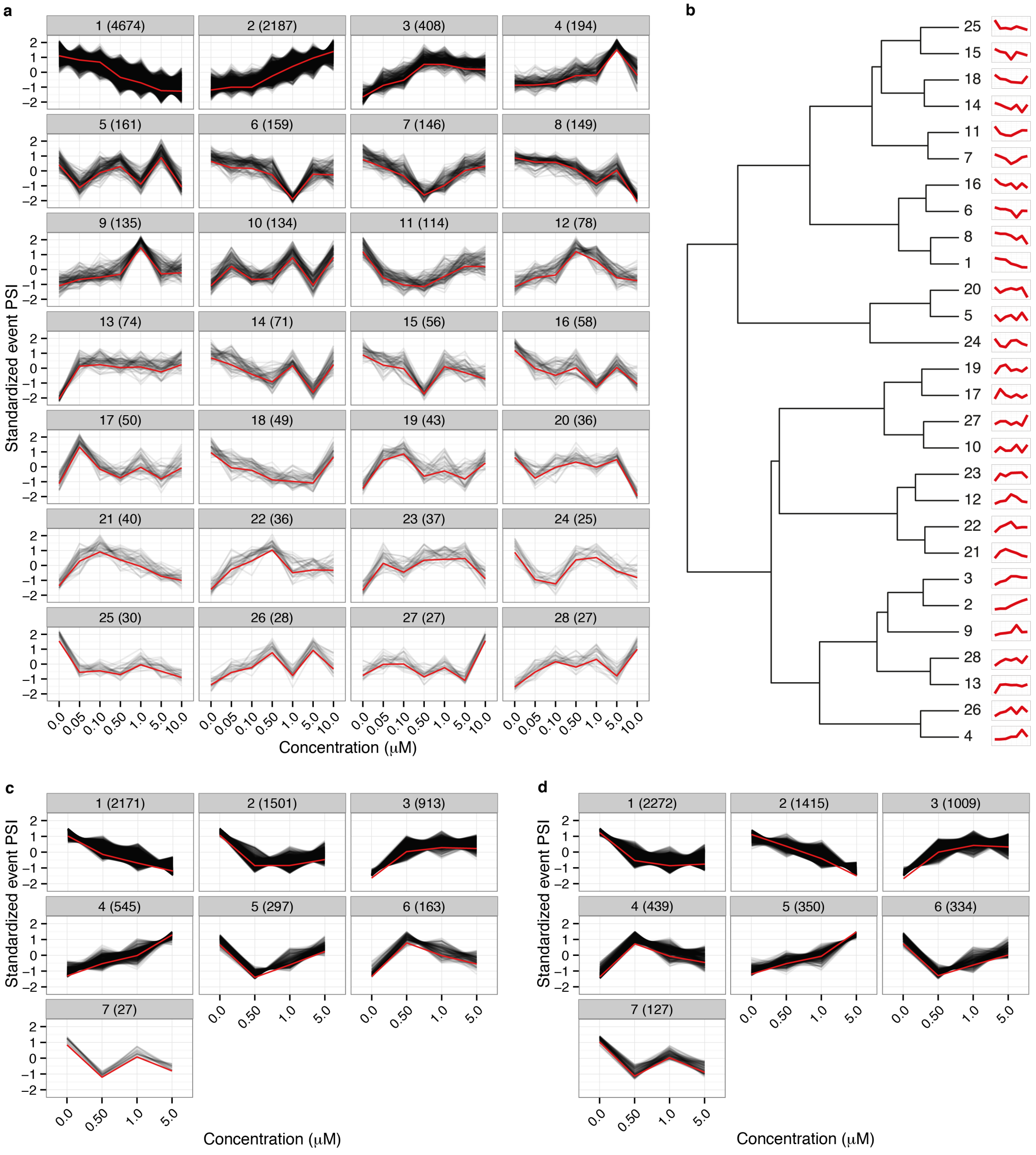

Supplementary Figure 17: AS event PSI clusters. **a** Clusters for the HCT116 unstranded RNA-Seq dataset. Black lines represent AS event PSI profiles. Red lines are cluster eigen-events. The number of events in each cluster is shown in parentheses in the cluster label. **b** Dendrogram showing the relationships between HCT116 unstranded RNA-seq PSI clusters. The dendrogram was obtained from the correlation between cluster eigen-events, branch lengths scaled proportionately. Red lines are cluster eigen-events from **a**. AS event PSI clusters for the **c** HCT116 stranded and **d** 184hTERT stranded RNA-Seq datasets.

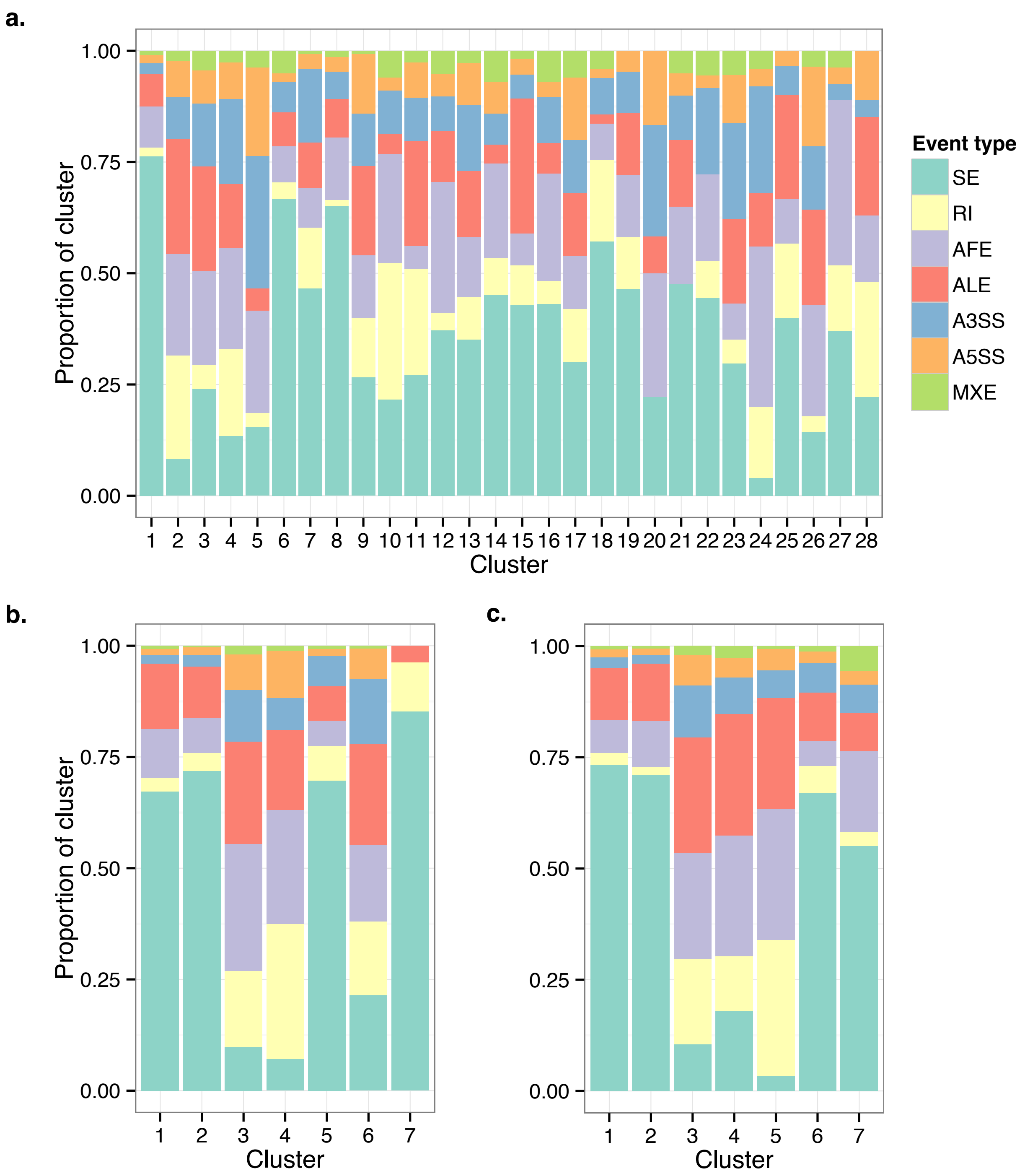

Supplementary Figure 18: AS event type proportions across AS PSI clusters. HCT116 **a** unstranded, **b** stranded RNA-Seq, and **c** 184hTERT datasets.

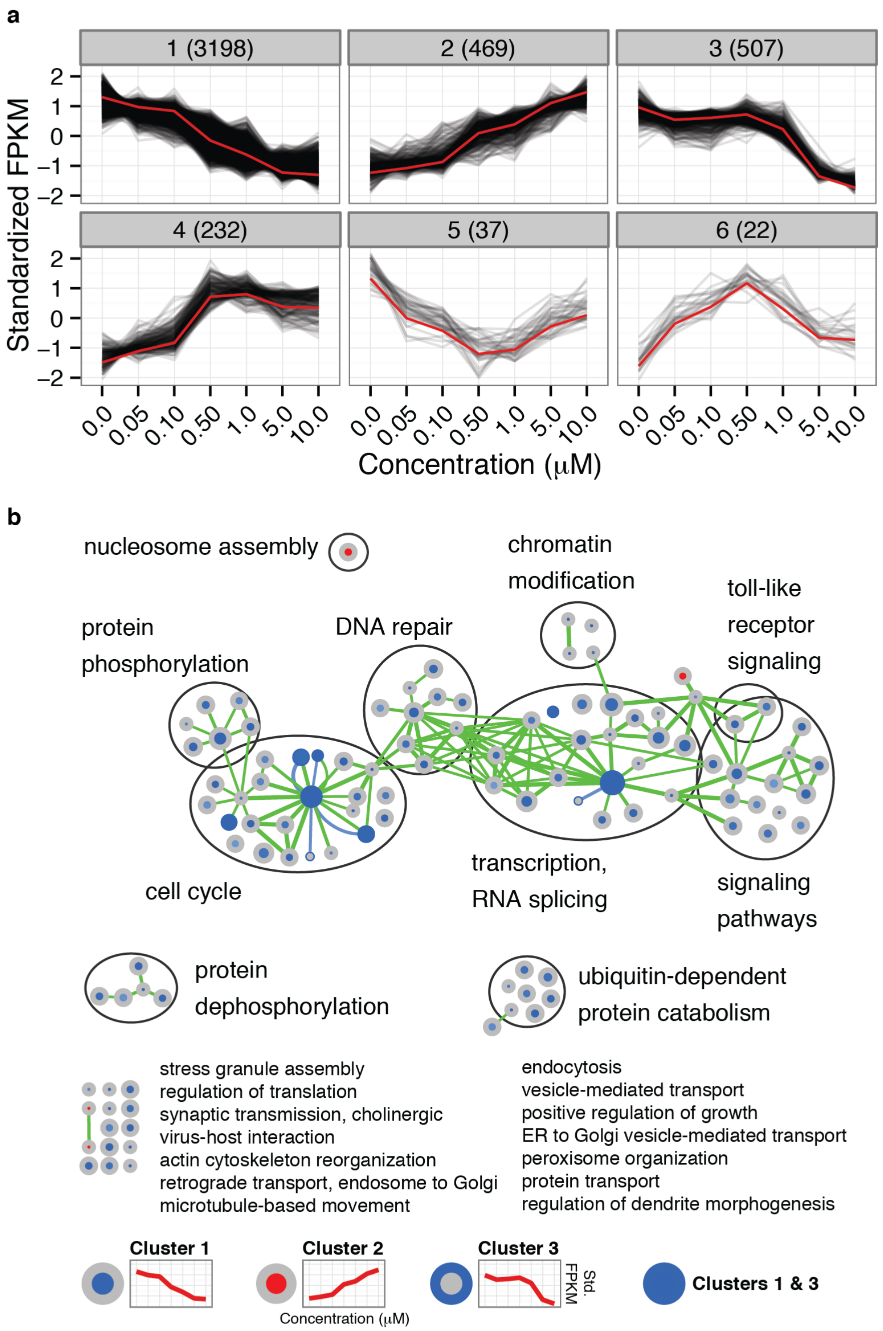

Supplementary Figure 19: Gene expression responses to T3 from FPKM analysis of RNA-seq libraries. **a** Standardized gene expression profiles from the HCT116 unstranded RNA-Seq dataset. Genes have been clustered using WGCNA [2] based on FPKM profiles. **b** Biological process enrichment map for differentially expressed genes in the HCT116 unstranded RNA-Seq dataset. Each node represents a GO biological process gene set. Red nodes represent biological processes enriched among up-regulated genes, likewise blue for down-regulated genes. Node cores are coloured blue when that gene set is enriched among genes in cluster 1, red for cluster 2. The outer ring is coloured blue when that gene set is enriched among genes in cluster 3. Edge thickness indicates the level of overlap between two gene sets, considering the set of up- or down-regulated genes.

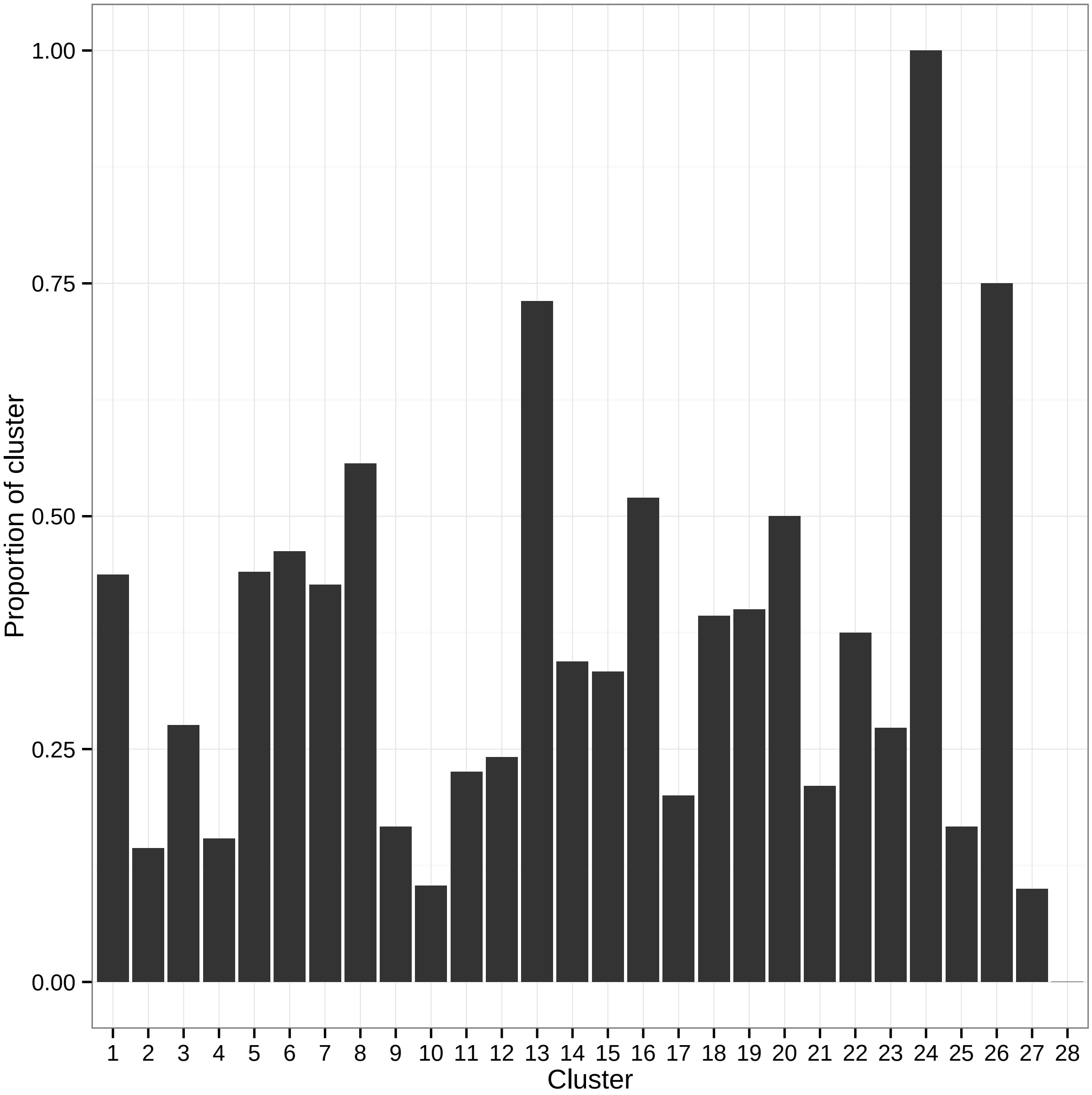

Supplementary Figure 20: Proportion of PSI clustered SE events that affect Ensembl annotated Pfam domains in the HCT116 unstranded RNA-Seq dataset.

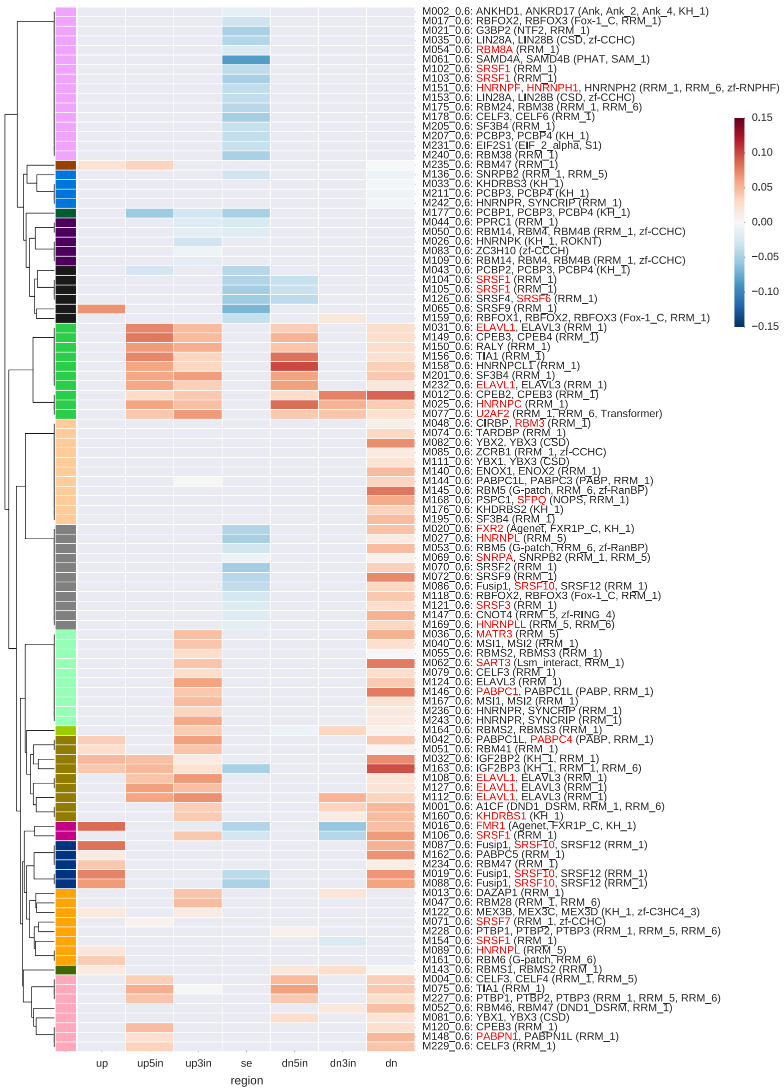

Supplementary Figure 21: Heatmap showing RNA binding protein (RBP) motifs with a significant density difference in PSI cluster 1 SE sequences compared to other PSI clusters. Heatmap is for HCT116 unstranded RNA-Seq dataset SE events. X-axis represents 7 SE event sequence regions: up (upstream exon), up5in and up3in (5′ and 3′ sections of the upstream intron), se (skipped exon), dn5in and dn3in (5′ and 3′ sections of the downstream intron), and dn (downstream exon). Y-axis shows the RBP motif id and RBP name (highlighted red: CLK interactors). Cells with non-significant differences are coloured grey. Coloured cells represent a positive (red) or negative (blue) effect size. Color bars on the left y-axis represent a possible motif cluster assignment based on significance and effect size patterns.

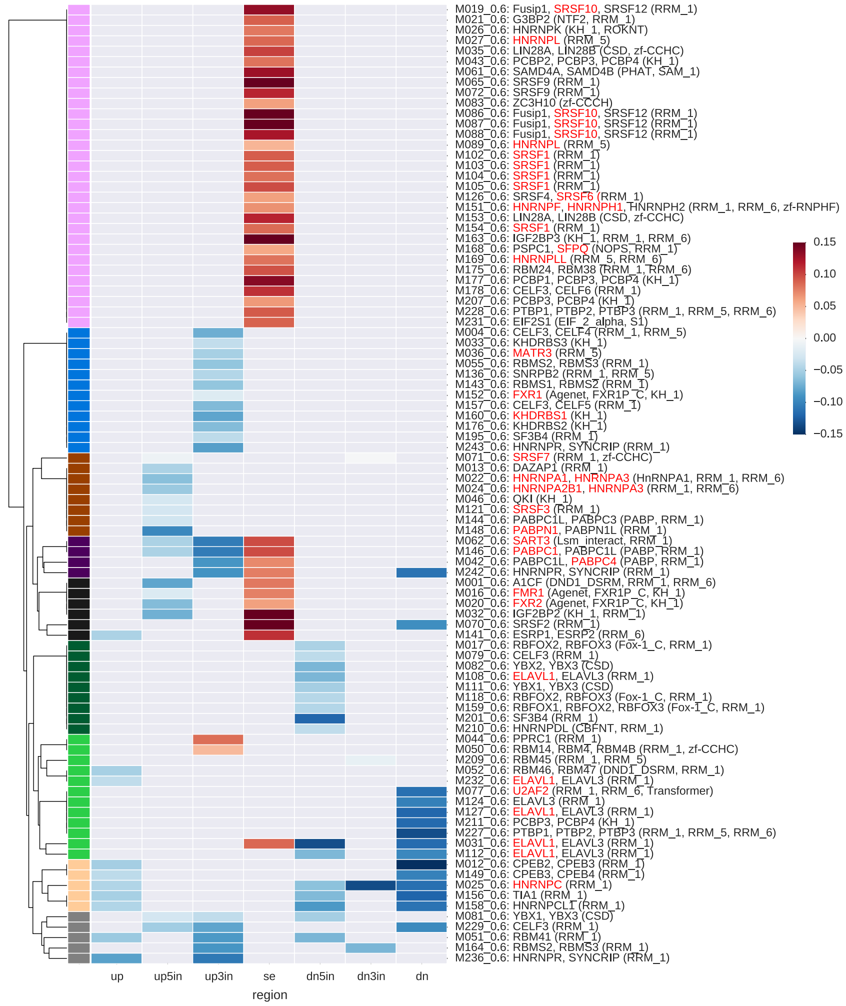

Supplementary Figure 22: Heatmap showing RNA binding protein (RBP) motifs with a significant density difference in PSI cluster 2 SE sequences compared to other PSI clusters. Heatmap is for HCT116 unstranded RNA-Seq dataset SE events. X-axis represents 7 SE event sequence regions: up (upstream exon), up5in and up3in (5′ and 3′ sections of the upstream intron), se (skipped exon), dn5in and dn3in (5′ and 3′ sections of the downstream intron), and dn (downstream exon). Y-axis shows the RBP motif id and RBP name (highlighted red: CLK interactors). Cells with non-significant differences are coloured grey. Coloured cells represent a positive (red) or negative (blue) effect size. Color bars on the left y-axis represent a possible motif cluster assignment based on significance and effect size patterns.

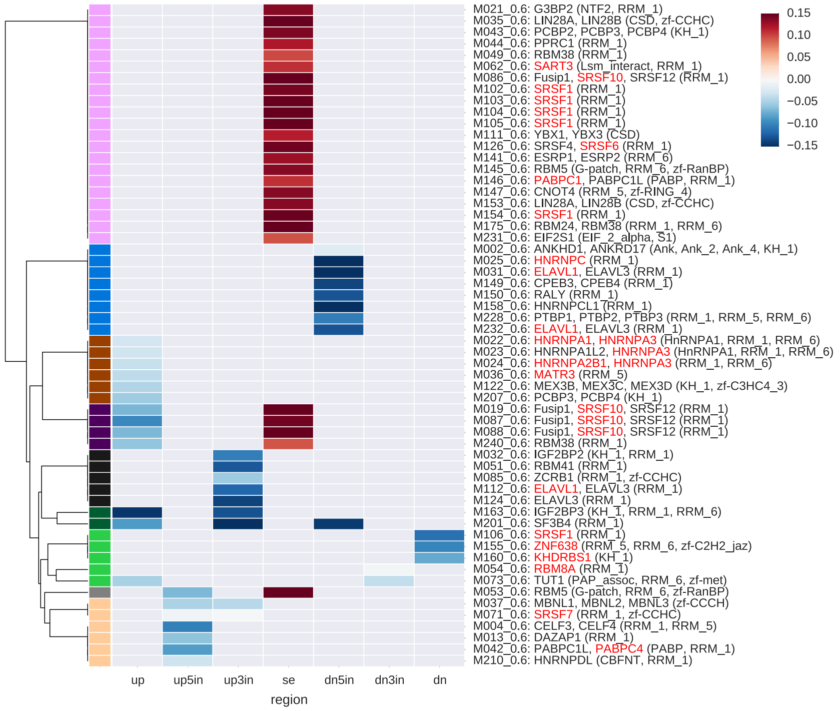

Supplementary Figure 23: Heatmap showing RNA binding protein (RBP) motifs with a significant density difference in PSI cluster 3 SE sequences compared to other PSI clusters. Heatmap is for HCT116 unstranded RNA-Seq dataset SE events. X-axis represents 7 SE event sequence regions: up (upstream exon), up5in and up3in (5′ and 3′ sections of the upstream intron), se (skipped exon), dn5in and dn3in (5′ and 3′ sections of the downstream intron), and dn (downstream exon). Y-axis shows the RBP motif id and RBP name (highlighted red: CLK interactors). Cells with non-significant differences are coloured grey. Coloured cells represent a positive (red) or negative (blue) effect size. Color bars on the left y-axis represent a possible motif cluster assignment based on significance and effect size patterns.

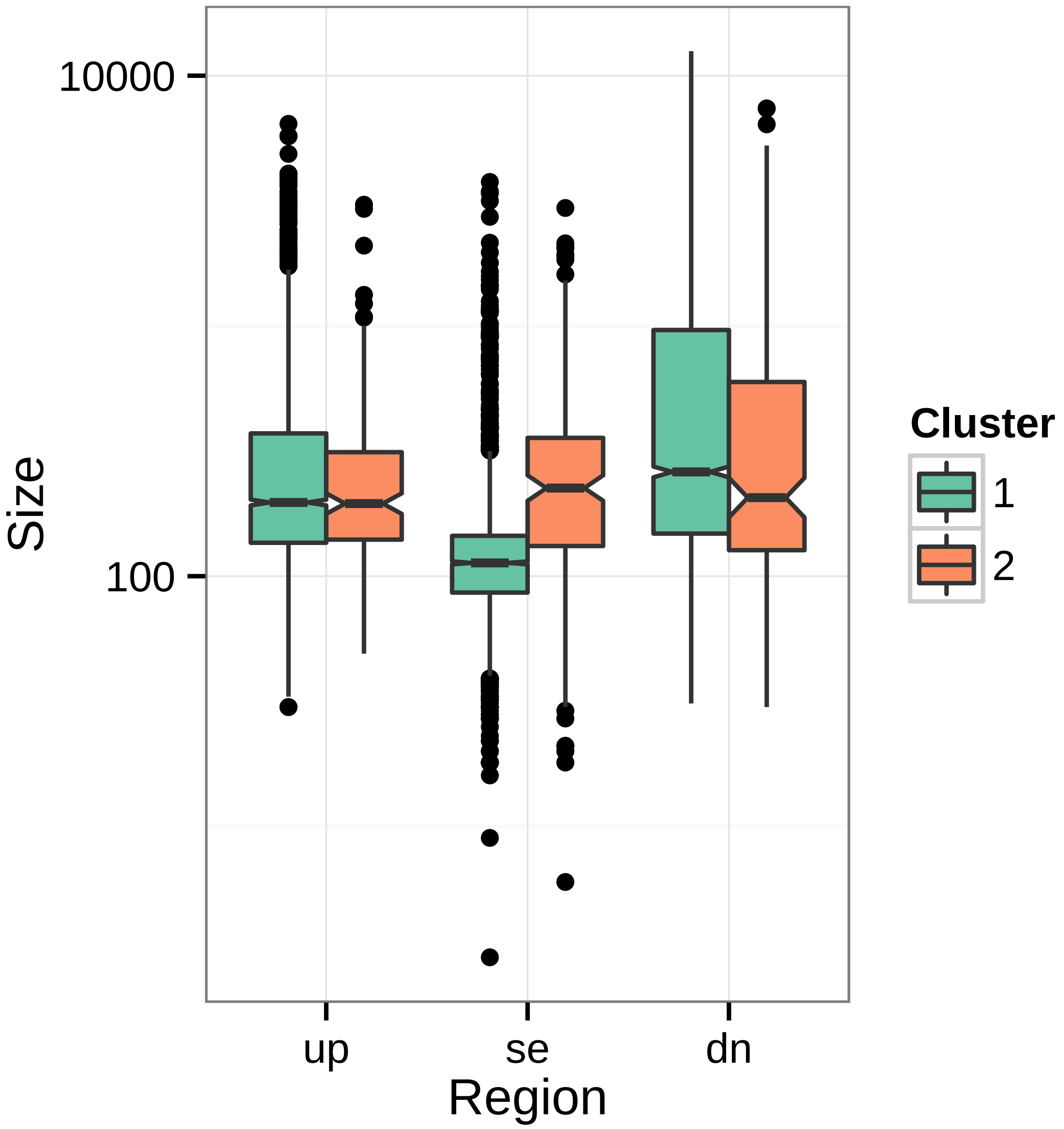

Supplementary Figure 24: Exon size (bp) boxplots for SE events in PSI clusters 1 and 2. dn: downstream exon, se: skipped exon, up: upstream exon.

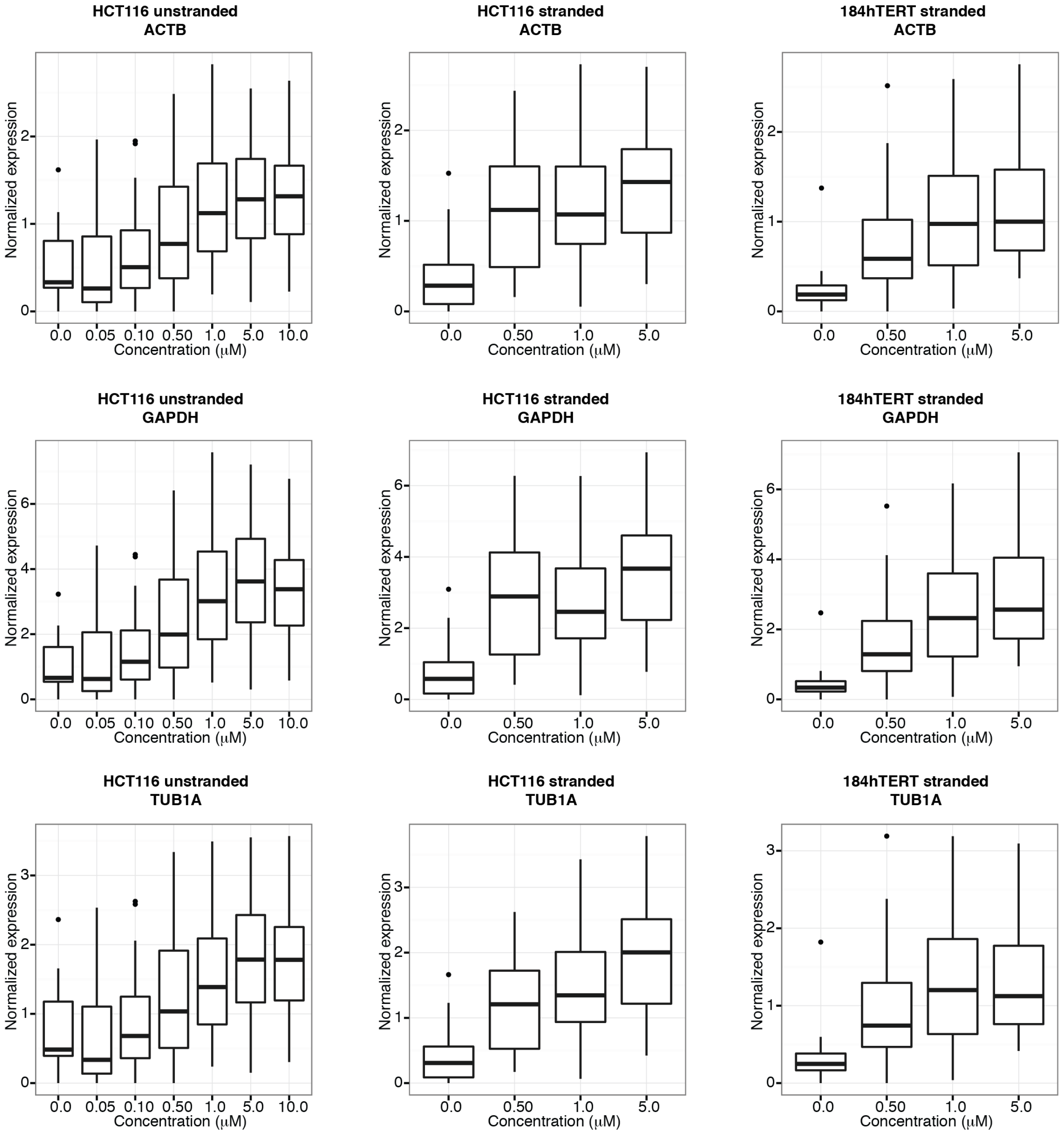

Supplementary Figure 25: Normalized conjoined gene expression boxplots across T3 concentrations for HCT116 unstranded, HCT116 stranded and 184hTERT RNA samples. Conjoined gene expression has been normalized to *ACTB*, *GAPDH*, *TUB1A* expression as indicated above each panel. This dataset is generated from the same RNA samples used to generate the corresponding RNA-Seq libraries analysed for AS events.

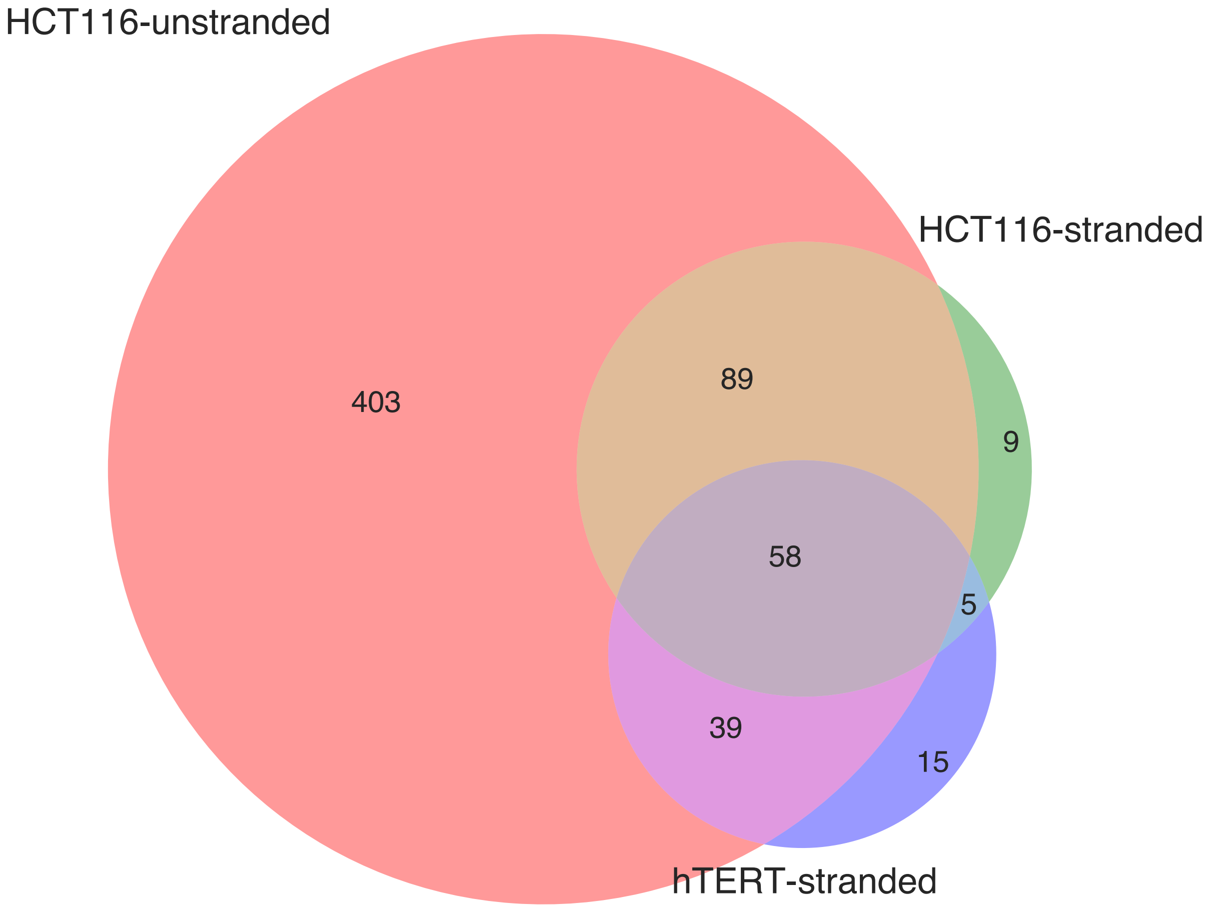

Supplementary Figure 26: Venn diagram of conjoined genes detected in the two HCT116 and one 184hTERT RNA-Seq datasets.

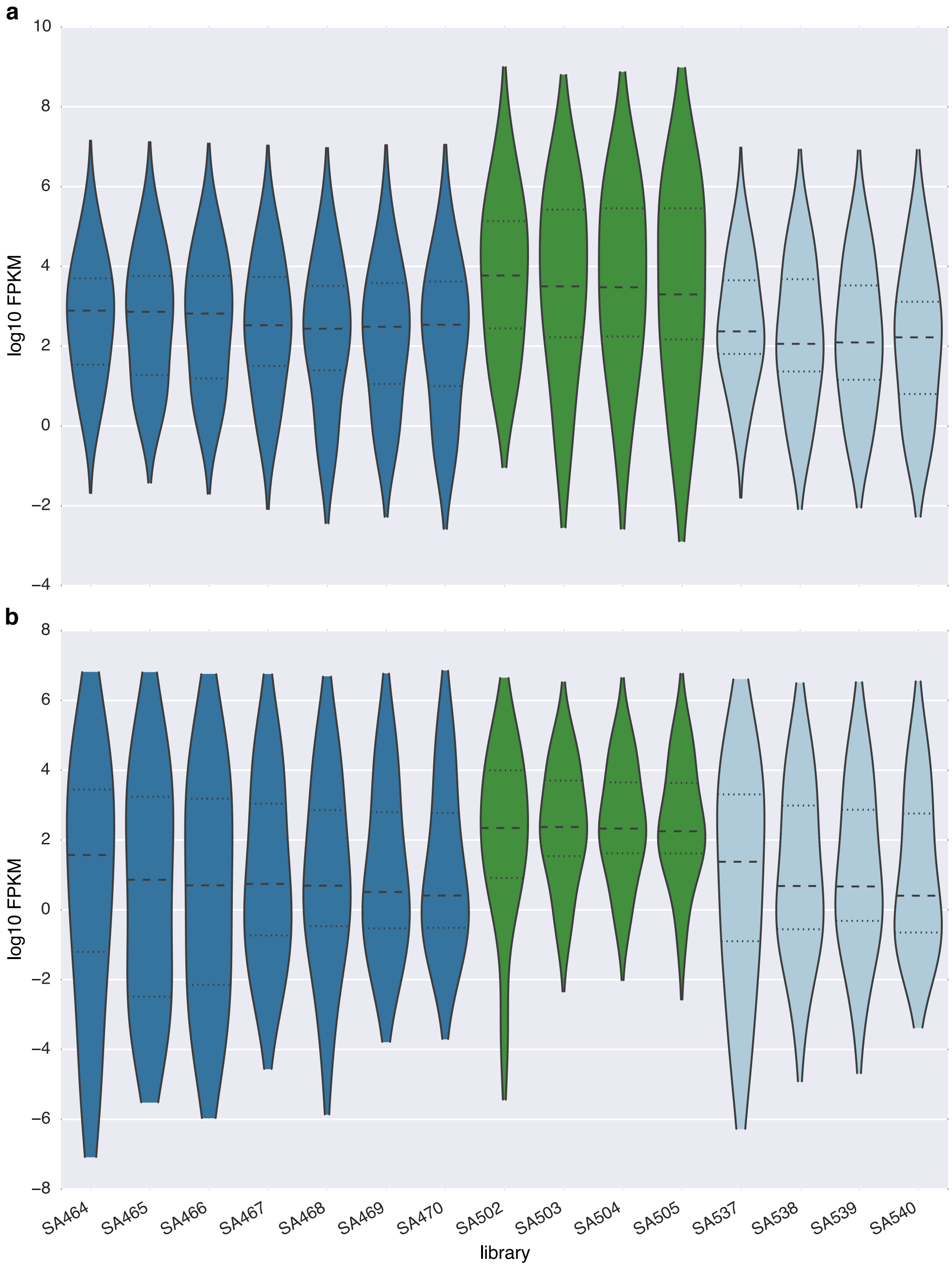

Supplementary Figure 27: Violin plots of log-transformed FPKM values for 184hTERT exclusive CG partners. **a** Upstream and **b** downstream gene partner FPKM values are plotted for both HCT116 RNA-Seq datasets and the 184hTERT dataset. SA464–470 are the HCT116 samples used for unstranded RNA-Seq (dark blue), SA537–540 are HCT116 samples used for stranded RNA-Seq (light blue), and SA502–505 are 184hTERT samples (green). Violin plots for each dataset are ordered by increasing T3 concentration.

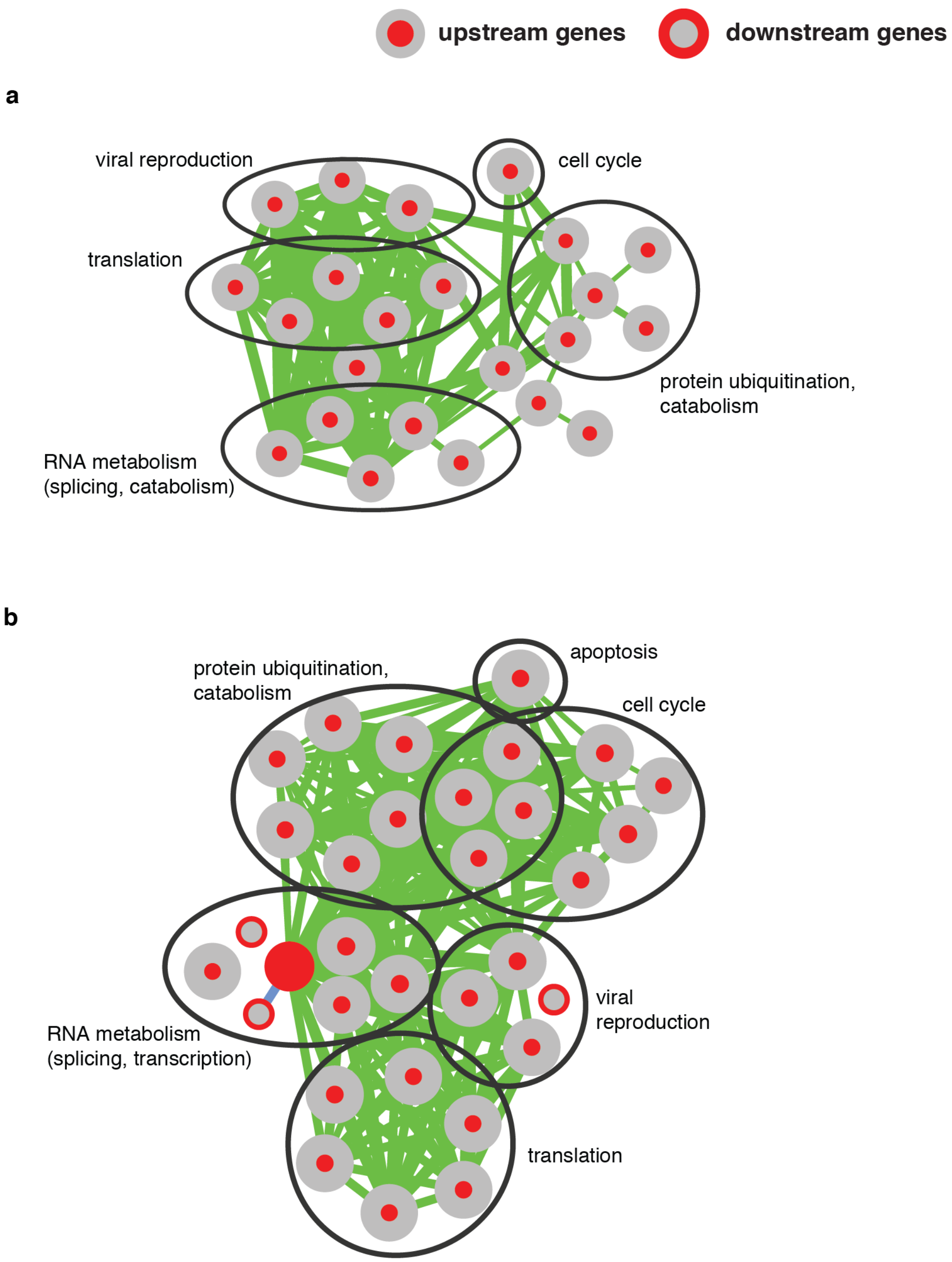

Supplementary Figure 28: Enrichment map for upstream and downstream partner conjoined genes. **a** HCT116 stranded and **b** 184hTERT RNA-Seq datasets. Each node represents a GO biological process gene set. Biological processes enriched in CG upstream partners have red cores, while biological processes enriched in downstream partners have red outer rings. Edge thickness indicates the level of CG partner overlap between gene sets.

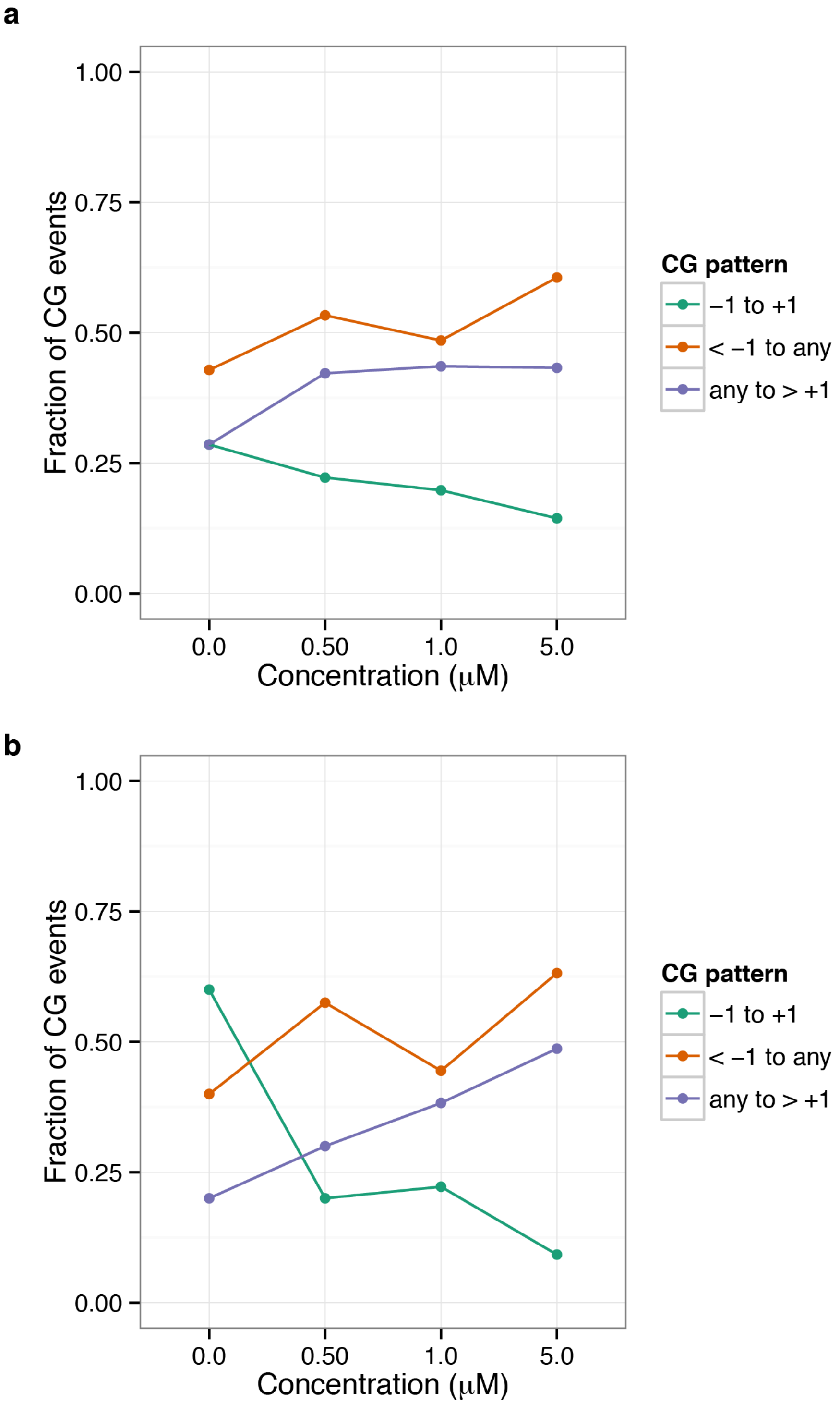

Supplementary Figure 29: CG splicing pattern proportions across T3 concentrations. **a** HCT116 stranded RNA-Seq dataset. **b** 184hTERT RNA-Seq dataset.

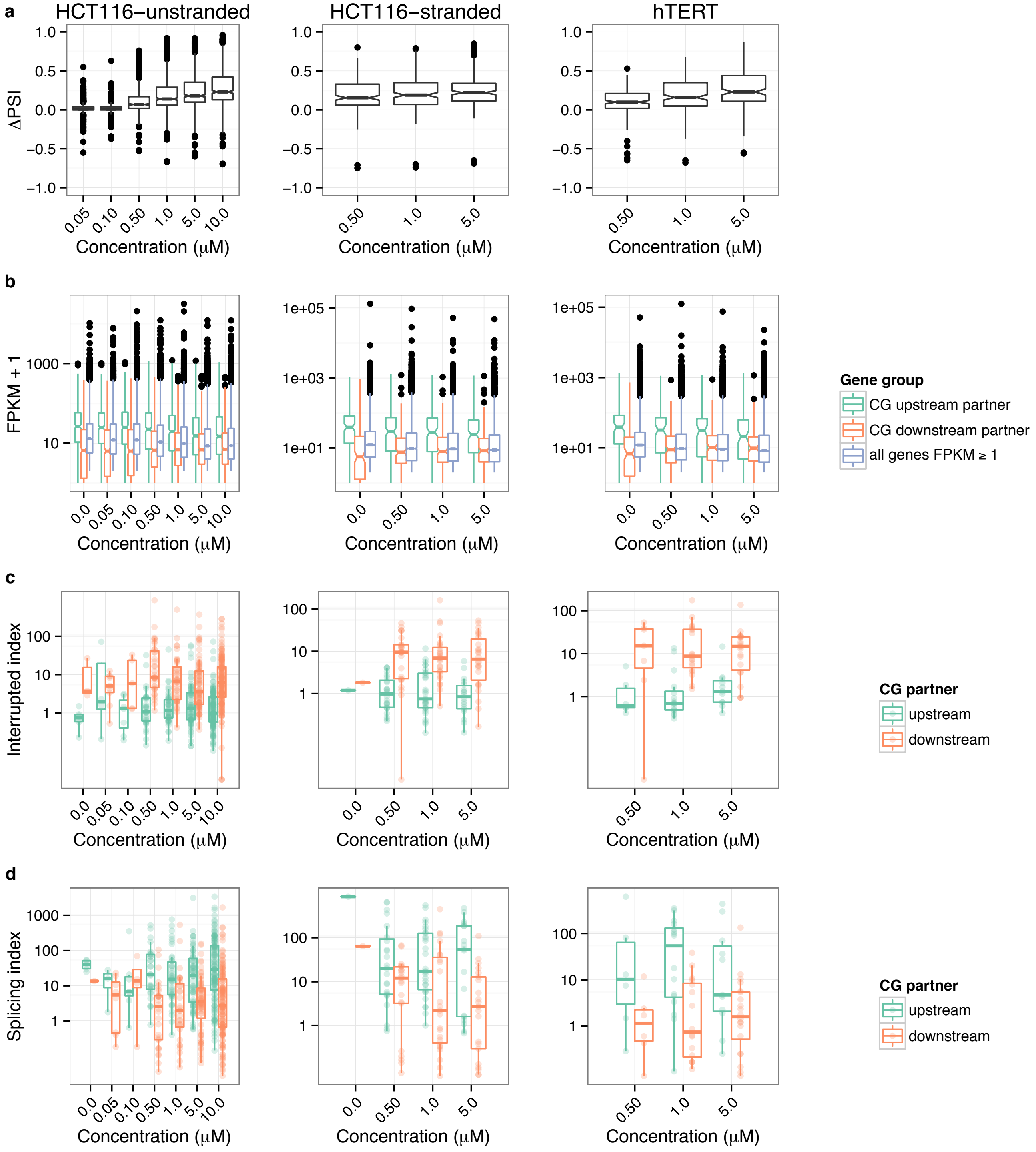

Supplementary Figure 30: Expression features of conjoined gene transcripts induced by inhibition of CLK. Figure legend on next page.

Supplementary Figure 30: (Previous page.) Expression and genomic features of conjoined genes for HCT116 unstranded libraries, stranded and 184hTERT, respectively (across page). **a** CG PSI change boxplots, per T3 treatment concentration. **b** Boxplots of FPKM values for CG upstream and downstream participants, compared to genes with FPKM ≥ 1 (*i.e.* expressed genes). **c** Boxplots of interrupted indices for CGs across T3 concentrations. Interrupted indices are calculated as the ratio of coverage between the portions of a gene retained and removed by CGs. Boxplots are shown for the upstream and downstream CG participants. **d** Boxplots of splicing indices for CGs across T3 concentrations. Splicing indices are calculated as the number of concordant read pairs spanning a CG splice junction in a CG participant, divided by the number of CG splice junction spanning reads that support the presence of a CG. Boxplots are shown for the upstream and downstream CG participants.

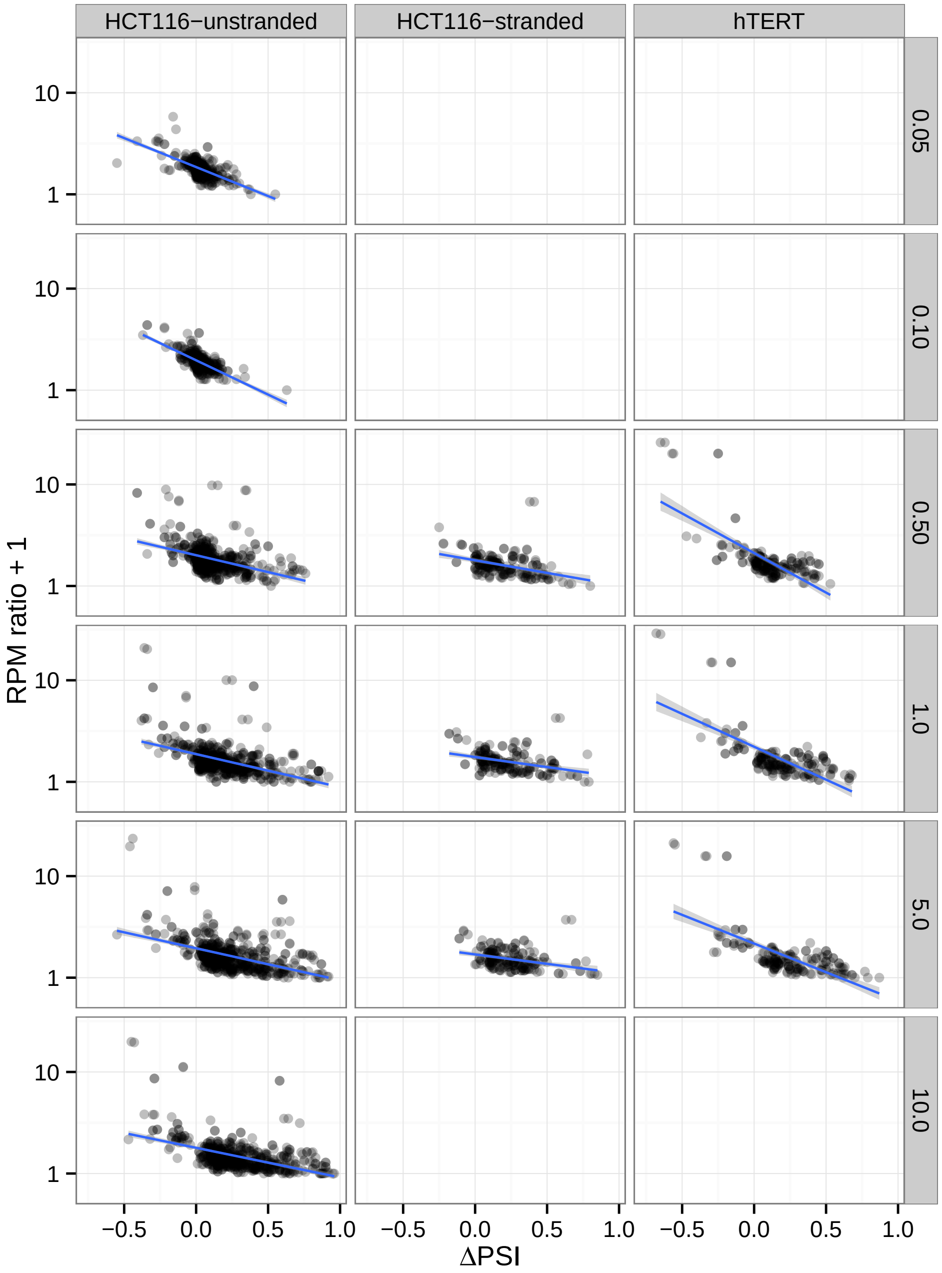

Supplementary Figure 31: Non-conjoined upstream transcript expression ratio *vs.* CG PSI change in the HCT116 and 184hTERT RNA-Seq datasets. Upstream non-conjoined transcript expression is reads per million (RPM) mapped reads supporting the non-conjoined isoform from the CG MISO analysis. RPM ratio is the RPM of the upstream gene in the treated sample divided by the RPM in the control sample. PSI change is the difference in PSI from the control sample to the treated sample for the CG event. Negative regression line slope indicates decrease in non-conjoined transcription with CG PSI increase.

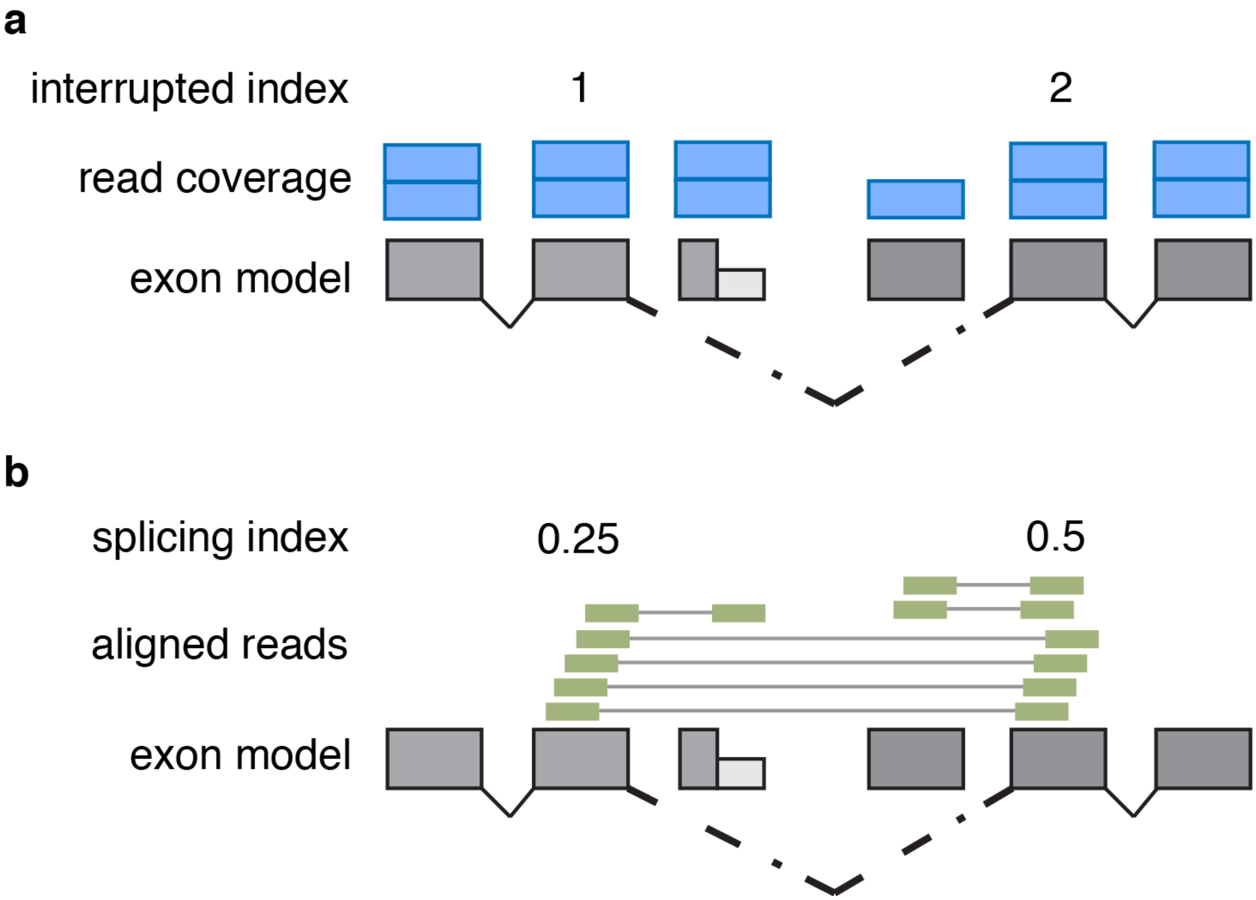

Supplementary Figure 32: Diagram describing interrupted and splicing indices. **a** Interrupted indices for the upstream (left) and downstream (right) CG partner genes. The interrupted index is the ratio of coverage between the retained and removed portion of a CG gene partner. Blue bars indicate level of read coverage for each exon (grey boxes). **b** Splicing indices for the upstream (left) and downstream (right) CG partner genes. The splicing index is the ratio of concordant to CG-supporting reads spanning the CG splice junction. Green bars indicate reads aligned to exons (grey boxes).

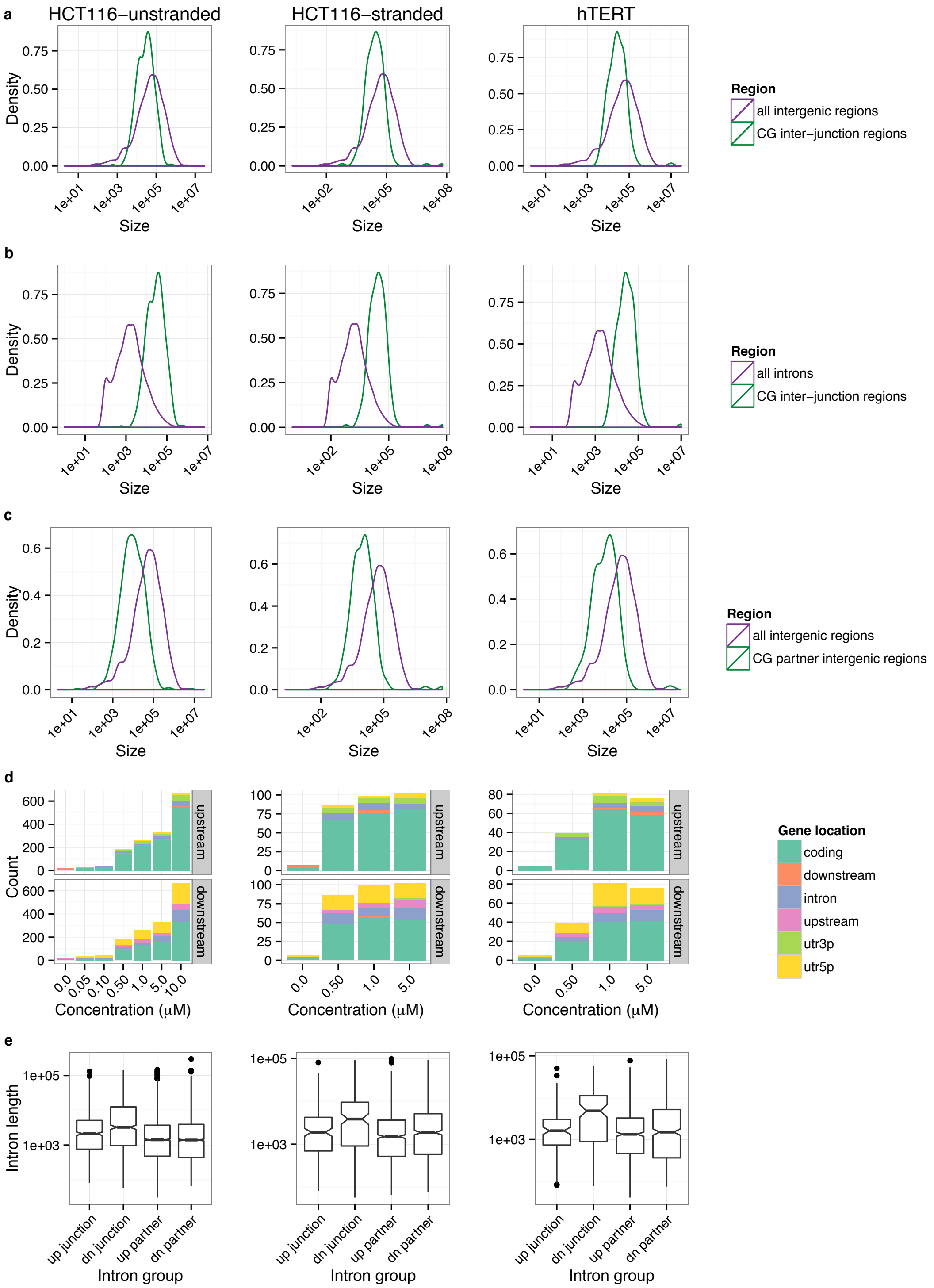

Supplementary Figure 33: Genomic features of conjoined gene transcripts induced by inhibition of CLK. Figure legend on next page.

Supplementary Figure 33: (Previous page.) Genomic features of conjoined genes for HCT116 unstranded libraries, stranded and 184hTERT, respectively (across page). **a** Density plot of CG splice junction distances *vs.* gene distances in the genome. **b** Density plot of CG splice junction distances *vs.* intron lengths of multi-exonic protein coding genes in the genome. **c** Density plot of CG participant distances *vs.* consecutive gene distances in the genome. **d** Barplots showing the number of CG splice junctions falling within different annotated gene locations, across T3 concentrations. Barplots for the upstream and downstream CG participants are shown. **e** Boxplots of intron lengths for introns adjacent to the upstream and downstream CG splice junction, and all introns in upstream and downstream CG participants.

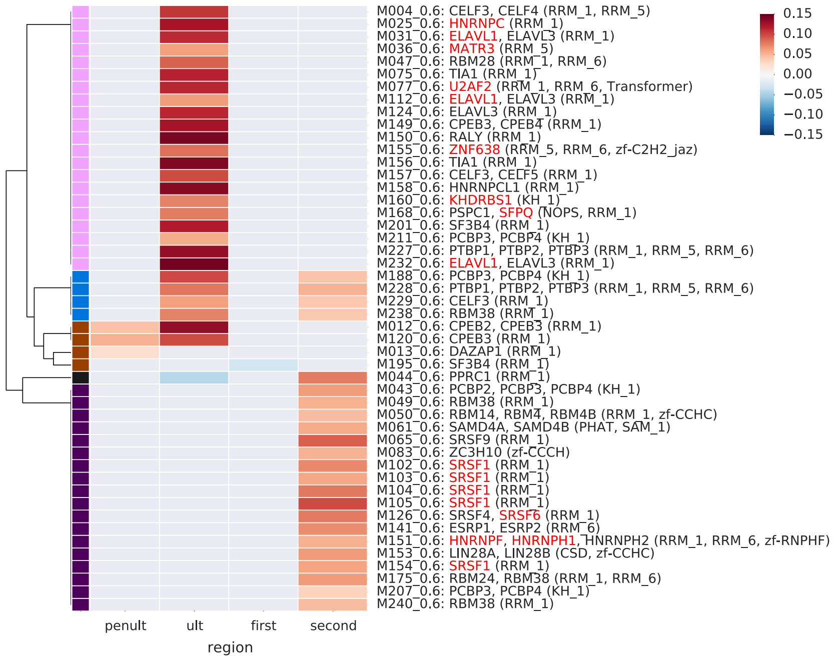

Supplementary Figure 34: Heatmap showing RNA binding protein (RBP) motifs with a significant density difference in CG sequences compared to non-CG sequences. Heatmap is for all HCT116 and 184hTERT CGs. X-axis represents 4 CG sequence regions: penultimate exon, ultimate exon (terminal exon of upstream participant), first exon downstream (first exon of downstream participant), second exon (downstream partner). Y-axis shows the RBP motif id and RBP name (highlighted red: CLK interactors). Cells with non-significant differences are coloured grey. Coloured cells represent a positive (red) or negative (blue) effect size. Color bars on the left y-axis represent a possible motif cluster assignment based on significance and effect size patterns.

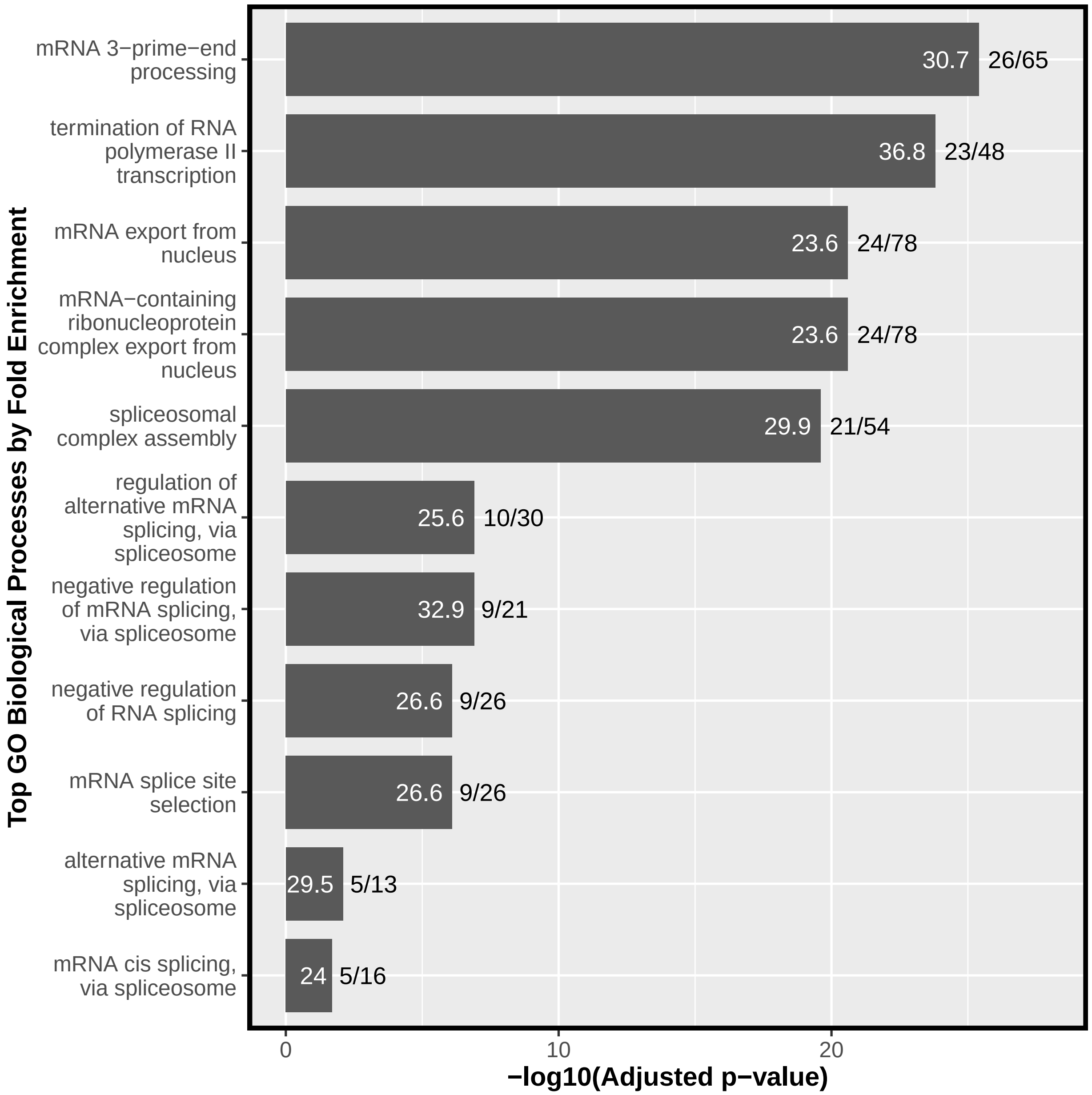

Supplementary Figure 35: Bar chart showing the top biological processes by fold enrichment as annotated by GeneOntology, for CLK2 IP-MS. Fold enrichment as annotated by GeneOntology (http://www.pantherdb.org/) [3] using the statistical overrepresentation test of the interactors from the IP-MS experiment against the statistical significance of the test as represented by the Bonferroni corrected P-value (in -log10 scale). The fractional value on the top of each bar is the number of genes in the dataset annotated belonging to the biological process over the total number of annotated genes in the human reference in that same category. The graph is filtered by the score of the functional enrichment testing [3] for each of the biological processes (indicated in white font on the bar respective bars; cut-off, Fold Enrichment >23.5) but sorted by their statistical significance value.

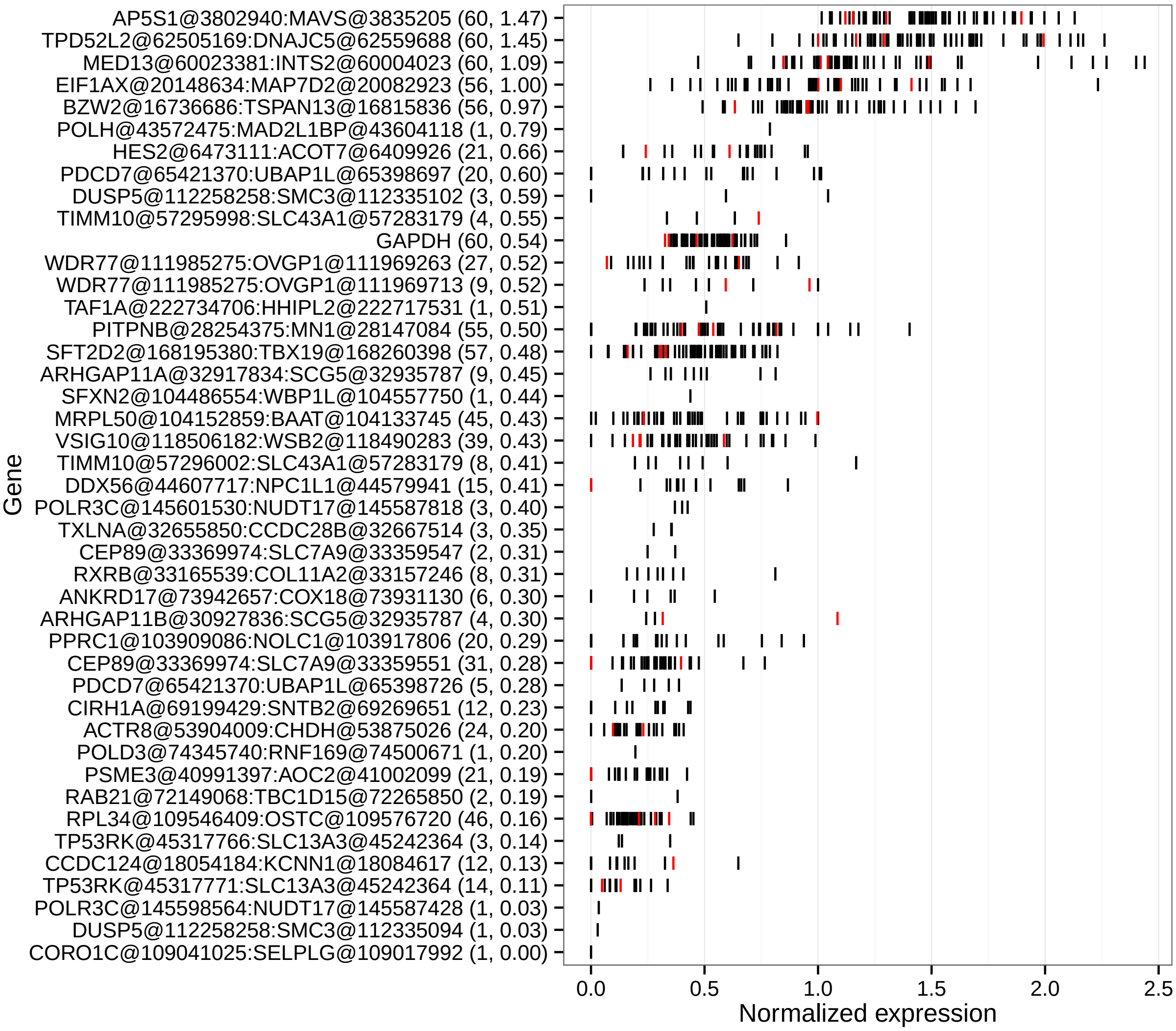

Supplementary Figure 36: Upper-quartile normalized expression of CGs and GAPDH for siRNA experiment targeting CG associated RNA-binding proteins. CGs are denoted as upstream-gene@junction:downstream-gene@junction. Red bars indicate expression in control (siNT) libraries. Number of observations and median expression in parentheses.

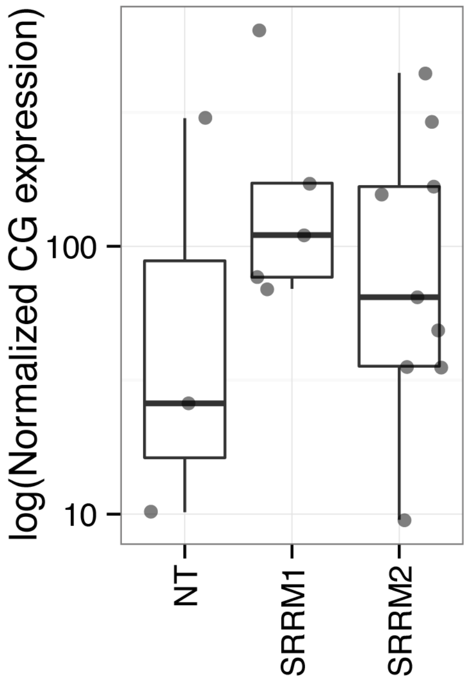

Supplementary Figure 37: Repeat quantification of CG expression 48 hours after siRNA knockdown of SRRM1/2. Expression determined by targeted NGS sequencing panel for CG events, expression normalized to a PCR amplicon for GAPDH transcripts.

Supplementary Table 1: Summary of the HTS screening conditions and T3 compound characteristics

Category	Parameter	Description
Assay	Type of assay	*in vitro*, Homogeneous Kinase Glo assay
	Target	SRPK1
	Primary measurement	Detection of Luminescence
	Key reagents	SRPK1(internal preparation), peptide (GRSRSRSRSRSRSRSR; customized, Thermo Fisher), ATP, Kinase Glo Reagents (Promega)
	Assay protocol	Compound was incubated with 30 ng/mL SPRK1 and 3 μM peptide (GRSRSRSRSRSRSRSR) for 10 min, and then kinase reaction was initiated by adding ATP to a final concentration of 0.5 μM, followed by incubation for 30 min. The kinase reaction was conducted in assay buffer composed of 25 mM HEPES (pH 7.5), 10 mM magnesium acetate, 1 mM DTT, 0.01% BSA, and 0.01% Tween-20. The kinase reaction was terminated by adding Kinase-Glo reagent. Remained ATP after enzyme reaction was detected as luminescent signals by Envision.
	Additional comments	N/A
Library	Library size	880,000
	Library composition	880,000 lead-like molecules
	Source	Takeda internal screening library.
	Additional comments	N/A
Screen	Format	384-well plates
	Concentration(s) tested	1 μM compound, 2% DMSO
	Plate controls	Total reaction for 0% inhibition and total reaction without enzyme for 100% inhibition. IC_{50}s of staurosporin were monitored in each batch.
	Reagent/ compound dispensing system	Multidrop Combi , FlexDrop, HORNET HTS100-SA
	Detection instrument and software	PerkinElmer, Envision
	Assay validation/QC	Z' > 0.5
	Correction factors	N/A
	Normalization	N/A
	Additional comments	N/A
Post-HTS analysis	Hit criteria	> 30% inhibition
	Hit rate	1.3%
	Additional assay(s)	Radioactive kinase activity assay was followed by first screening to remove false positives in Kinase Glo assay. ATP competitive enzyme assay and CLK1 kinase assay were conducted to profile mechanism of action and selectivity.
	Confirmation of hit purity and structure	Purity of compounds was checked by LC/MS.
	Additional comments	N/A

Supplementary Table 2: Summary of the HTS screening conditions and T3 compound characteristics

T3

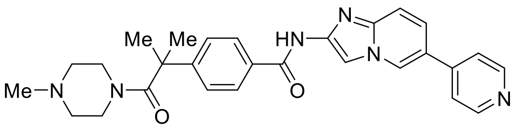

Category	Parameter	Description
Compound	Additional names	4-(2-methyl-1-(4-methylpiperazin-1-yl)-1-oxopropan-2-yl)-N-(6-(pyridin-4-yl)imidazo[1,2-a]pyridin-2-yl)benzamide
	Citation	
	Chemical descriptors	
	Chemical compound page	
	Entries in chemical databases	
	Availability	Synthetic procedure was described in Methods
	Additional comments	N/A
In vitro profiling	Target	CLK1/2
	Potency	0.67 nM (IC_{50}) against CLK1 and 15 nM (IC_{50}) against CLK2 in kinase activity assay; see Fig 1b
	Selectivity	Profiled against CLK3, DYRK1A, 1B and 70 kinases. Nearly 100 times selective toward CLK1/2 over DYRKs; Supplementary Fig 3a
	Potential reactivities	Not known
	SAR	Pyridine ring is important for tight binding with Lys193 and N-methylpiperazine moiety which expose in solvent increase activity.
	Mechanism of action	ATP competitive inhibition. IC_{50} values against CLK2 were changed on the basis of the Cheng-Prusoff equation, with 0.76 nM (IC_{50}) under 20μM ATP (1 x Km) and 15 nM (IC_{50}) under 1mM ATP (50 x Km) ; see Supplementary Fig 1c; See also the structural docking model (Supplementary Fig 2) which is compatible with ATP-competitive mode.
	Structure of target-probe complex	N/A
	Additional comments	N/A
Cellular profiling	Validation of cellular target	Dose-dependently inhibited phospho SR, SRSF1, SRSF4, and SRSF6, at 1 μM in HCT116 cells, while KH-CB19, which has weaker inhibitory activity against CLKs, shows an intermediate dose dependent effect at 10 μM; see Supplementary Fig 5. Additionally, T3 induced exon skipping of S6K and con-joined gene in HCT 116 and 184h TERT cells, whereas KH-CB19 had intermediate effect; see Supplementary Fig 10. RNAi against CLK isoforms followed by RNA-seq shows a similar splicing event network is perturbed with 55% events in common to T3.
	Validation of cellular specificity	Global RNA-seq strongly shows the expected canonical splicing event pattern for CLK function in exon recognition as the major event type – ie. skipped exons and retained introns. This is the main biological point of the paper.

Supplementary Table 3: Stability of T3 in tissue culture medium.

Incubation time (hours)	Concentration of T3 (µM)	Residual rate (%)
Initial	5.50	100
6	5.44	98.9
24	5.38	98.8
48	5.30	98.7

Supplementary Table 4: Summary of RNA-Seq libraries

Library	Cell line	Inhibitor	Treatment (µM/siRNA)	Library type	Mapped reads	ΔAS	CG events
SA464	HCT116	T3	0.0	unstranded	128206183	-	25
SA465	HCT116	T3	0.05	unstranded	133328391	799	33
SA466	HCT116	T3	0.10	unstranded	123013369	1088	42
SA467	HCT116	T3	0.50	unstranded	145759273	4474	190
SA468	HCT116	T3	1.0	unstranded	109950486	5354	261
SA469	HCT116	T3	5.0	unstranded	134334174	6751	339
SA470	HCT116	T3	10.0	unstranded	207512120	7761	681
SA537	HCT116	T3	0.0	stranded	96393177	-	7
SA538	HCT116	T3	0.50	stranded	106916112	3290	90
SA539	HCT116	T3	1.0	stranded	111327564	3909	101
SA540	HCT116	T3	5.0	stranded	106445336	4143	104
SA502	184hTERT	T3	0.0	stranded	125448803	-	5
SA503	184hTERT	T3	0.50	stranded	98321379	3369	40
SA504	184hTERT	T3	1.0	stranded	112028493	4162	81
SA505	184hTERT	T3	5.0	stranded	80923086	4158	76
SA564	HCT116	RNAi	CLK1	stranded	107680228	608	11
SA565	HCT116	RNAi	CLK2	stranded	123650625	340	21
SA566	HCT116	RNAi	CLK3	stranded	110354904	497	13
SA567	HCT116	RNAi	CLK4	stranded	152255547	351	23
SA568	HCT116	RNAi	CLK1,CLK2,CLK3	stranded	147157298	521	25
SA569	HCT116	RNAi	CLK3,CLK4	stranded	103064592	452	10
SA570	HCT116	RNAi	CLK1,CLK2,CLK4	stranded	122441796	443	17
SA571	HCT116	RNAi	CLK1,CLK2,CLK3,CLK4	stranded	115411269	451	14
SA572	HCT116	RNAi	NT3	stranded	115576925	-	9
SA573	HCT116	RNAi	None	stranded	131430447	209	18
SA612	HCT116	KH-CB19	0.0	stranded	118668675	-	7
SA618	HCT116	KH-CB19	0.2	stranded	122359066	286	7
SA619	HCT116	KH-CB19	0.5	stranded	127295945	157	4
SA620	HCT116	KH-CB19	1.0	stranded	113583588	142	6
SA621	HCT116	KH-CB19	5.0	stranded	107621728	214	11
SA622	HCT116	KH-CB19	10.0	stranded	141665393	284	19
SA828	HCT116	T3	0.0	stranded	136142374	-	20
SA828-R1	HCT116	T3	0.0	stranded	155666036	-	27
SA830	HCT116	T3	0.50	stranded	131371945	4301	169
SA830-R1	HCT116	T3	0.50	stranded	119932780	4351	173
SA831	HCT116	T3	1.0	stranded	150316252	5486	259
SA831-R1	HCT116	T3	1.0	stranded	140090439	5763	299
SA832	HCT116	T3	5.0	stranded	129726673	5668	397
SA832-R1	HCT116	T3	5.0	stranded	133619826	5734	334

Supplementary Table 5: Summary of PacBio libraries

Library	Cell line	Inhibitor	Treatment (µM)	Mapped reads	CG candidate events
PacBio0	HCT116	T3	0.0	836341	425
PacBio0.5	HCT116	T3	0.5	1135506	1484
PacBio5	HCT116	T3	5.0	1114255	2077

Supplementary Table 6: Level of target knock down in *CLK* siRNA treated HCT116 samples determinned by qRT-PCR. The relative expressions were calculated against a non targetting siRNA (NT3) and normalized to the expression of *GAPDH*.

		Level of knockdown			
Sample	siRNA targets	CLK1	CLK2	CLK3	CLK4
SA564	NT3	1	1	1	1
SA565	CLK1	80%			
SA566	CLK2		70%		
SA567	CLK3			90%	
SA568	CLK4				85%
SA569	CLK1+2+3	50%	45%	99%	
SA570	CLK3+4			70%	45%
SA571	CLK1+2+4	50%	45%		60%
SA572	CLK1+2+3+4	55%	55%	70%	45%

Supplementary Table 7: Summary of MiSeq targeted-sequencing libraries

Library	Cell line	Inhibitor	Treatment (µM/siRNA)	Mapped reads	CG events
SA464	HCT116	T3	0.0	1072028	15
SA465	HCT116	T3	0.05	1692476	23
SA466	HCT116	T3	0.10	2276213	22
SA467	HCT116	T3	0.50	2792373	35
SA468	HCT116	T3	1.0	2718358	36
SA469	HCT116	T3	5.0	3167103	37
SA470	HCT116	T3	10.0	2991186	35
SA537	HCT116	T3	0.0	1993739	17
SA538	HCT116	T3	0.50	2458757	36
SA539	HCT116	T3	1.0	4044954	38
SA540	HCT116	T3	5.0	2528781	35
SA502	184hTERT	T3	0.0	1163379	10
SA503	184hTERT	T3	0.50	2691585	26
SA504	184hTERT	T3	1.0	3469214	33
SA505	184hTERT	T3	5.0	2436103	35
siCPEB2-1	HCT116	RNAi	siCPEB2	1795434	15
siCPEB3-1	HCT116	RNAi	siCPEB3	1137842	11
siCPEB3-2	HCT116	RNAi	siCPEB3	1222301	14
siDAZAP1-1	HCT116	RNAi	siDAZAP1	1295516	14
siELAVL1-1	HCT116	RNAi	siELAVL1	1282781	13
siELAVL1-2	HCT116	RNAi	siELAVL1	1386141	13
siELAVL3-4	HCT116	RNAi	siELAVL3	1636686	15

Continued on next page

Supplementary Table 7 – *Continued from previous page*

Library	Cell line	Inhibitor	Treatment (µM/siRNA)	Mapped reads	CG events
siHNRNPC-4	HCT116	RNAi	siHNRNPC	435560	15
siHNRNPF-3	HCT116	RNAi	siHNRNPF	452086	16
siHNRNPH1-3	HCT116	RNAi	siHNRNPH1	623350	14
siHNRNPH1-4	HCT116	RNAi	siHNRNPH1	451090	10
siHNRNPH2-1	HCT116	RNAi	siHNRNPH2	414926	14
siHNRNPH2-4	HCT116	RNAi	siHNRNPH2	589859	13
siKHDRBS1-1	HCT116	RNAi	siKHDRBS1	434422	21
siKHDRBS1-4	HCT116	RNAi	siKHDRBS1	459043	15
siLIN28A-4	HCT116	RNAi	siLIN28A	416999	14
siLIN28B-2	HCT116	RNAi	siLIN28B	536707	11
siMATR3-1	HCT116	RNAi	siMATR3	988789	10
siMATR3-2	HCT116	RNAi	siMATR3	544399	12
siNT-Plate-1	HCT116	RNAi	siNT	1341437	9
siNT-Plate-2	HCT116	RNAi	siNT	327807	15
siNT-Plate-3	HCT116	RNAi	siNT	357371	12
siNT-Plate-4	HCT116	RNAi	siNT	1107507	18
siPCBP2-1	HCT116	RNAi	siPCBP2	598853	14
siPCBP2-4	HCT116	RNAi	siPCBP2	603853	11
siPCBP3-1	HCT116	RNAi	siPCBP3	837908	14
siPCBP3-2	HCT116	RNAi	siPCBP3	1100105	13
siPCBP4-3	HCT116	RNAi	siPCBP4	1538869	16
siPCBP4-4	HCT116	RNAi	siPCBP4	1526952	11
siPRPF8-4	HCT116	RNAi	siPRPF8	1485583	11
siPTBP1-2	HCT116	RNAi	siPTBP1	354449	13
siPTBP1-4	HCT116	RNAi	siPTBP1	385453	13
siPTBP2-2	HCT116	RNAi	siPTBP2	1097976	16
siPTBP2-4	HCT116	RNAi	siPTBP2	1460952	14
siPTBP3-3	HCT116	RNAi	siPTBP3	1428639	15
siRALY-1	HCT116	RNAi	siRALY	407811	13
siRALY-2	HCT116	RNAi	siRALY	654231	11
siRBM24-3	HCT116	RNAi	siRBM24	364338	13
siRBM24-4	HCT116	RNAi	siRBM24	498238	12
siRBM38-2	HCT116	RNAi	siRBM38	1247874	13
siRBM38-3	HCT116	RNAi	siRBM38	1407111	13
siRBM4-1	HCT116	RNAi	siRBM4	904572	15
siRBM4-2	HCT116	RNAi	siRBM4	509191	18
siSAMD4B-1	HCT116	RNAi	siSAMD4B	987735	17
siSAMD4B-4	HCT116	RNAi	siSAMD4B	865785	13
siSFPQ-3	HCT116	RNAi	siSFPQ	8304084	13
siSFPQ-4	HCT116	RNAi	siSFPQ	2310387	10
siSRRM2-3	HCT116	RNAi	siSRRM2	1401827	16
siSRRM2-4	HCT116	RNAi	siSRRM2	1343687	17
siSRSF1-1	HCT116	RNAi	siSRSF1	1119163	15
siSRSF1-2	HCT116	RNAi	siSRSF1	1488973	18
siSRSF1-3	HCT116	RNAi	siSRSF1	1264966	17
siSRSF4-2	HCT116	RNAi	siSRSF4	3156452	12
siSRSF4-4	HCT116	RNAi	siSRSF4	1115689	14
siSRSF6-1	HCT116	RNAi	siSRSF6	754119	16

Continued on next page

Supplementary Table 7 – *Continued from previous page*

Library	Cell line	Inhibitor	Treatment (µM/siRNA)	Mapped reads	CG events
siSRSF6-2	HCT116	RNAi	siSRSF6	665174	11
siSRSF9-3	HCT116	RNAi	siSRSF9	595660	17
siTIA1-1	HCT116	RNAi	siTIA1	1076817	12
siU2AF2-3	HCT116	RNAi	siU2AF2	422170	12
siU2AF2-4	HCT116	RNAi	siU2AF2	528991	14
NT-48hr	HCT116	RNAi	siNT	208132	3
SRRM1-48hr	HCT116	RNAi	siSRRM1	139746	5
SRRM2-48hr	HCT116	RNAi	siSRRM2	337490	9

Supplementary Note 1

In the past, several inhibitors have been developed to target CLK proteins each with limited interactions with the different CLK isoforms and inhibitory effects achieved with relatively high compound concentrations [4, 5]. TG003, a permeable benzothiazole compound [5], does not inhibit CLK3 and shows cross reactivity with casein kinase (CK1d and CK13), dual-specificity tyrosine phosphorylation-regulated kinase (DYRK1B), Yeast Sps1/Ste20-related kinase (YSK4) and proviral insertion site in Moloney Murine Leukemia Virus (PIM) kinase isoforms [6]. Recently, KH–CB19 (dichloroindolyl enaminonitriles) has been described [4] and although more potent than TG003, still has sub-optimal potency, selectivity and physiochemical properties.

To arrive at compound T3 (Supplementary Fig. 1a) we investigated the SAR surrounding a reference scaffold compound identified by a high throughput screen (see Supplementary Tables 1, 2 for the summary conditions of the screen). Compound 1 (Cpd-1)(Supplementary Fig. 1b) was identified by HTS of our library and has been previously described [1]. Our effort was focused on improvement of CLK2 inhibitory activity. Introduction of pyridine ring into the 6-position (R2) of imidazopyridine core boosted inhibitory activity (Supplementary Fig. 1b) likely because the nitrogen atom of the pyridine may tightly bind Lys193 (compound 2). Considering R1, a piperazine moiety was tolerated (compound 3) and N-alkylation increased activity. T3 and compound 4 showed significant CLK2 inhibition (IC50: 15 nM). T3 was selected as a tool compound due to its high solubility in water. The ATP competitive nature of the compound is revealed by the shift in CLK2 inhibition curves under different concentrations of ATP (Supplementary Fig. 1c) and by the structural docking model for T3 (Supplementary Fig. 2).

For the docking model, T3 was docked to the crystal structure of CLK2 (PDB ID: 3NR9) using the Induced Fit Docking protocol (Schrödinger, LLC, New York). The crystal structure was protonated and minimized using the Protein Preparation Wizard, and then the ligand and water molecules were removed. T3 structure was prepared using the LigPrep (Schrödinger, LLC, New York). The docking study was performed with two hydrogen bond constraints aimed at interactions with Leu246. The pose with the top rank of the IFDScore was selected.

The structural features of T3 result in high specificity and potency of this molecule for CLK family inhibition. The specificity of T3 to CLK family members was further confirmed using other dual specificity kinases such as DYRK1A and DYRK1B as substrates in the kinase enzymatic assay which showed 200–300 times weaker inhibition compared with CLK inhibition (Fig. 3a). Due to the very high potency of T3 against CLK protein kinases, a higher ATP concentration of 1 mM was used in the kinase assay to obtain measurable inhibition in comparison with the previously published data. To further confirm specificity, the inhibition spectrum of this compound was also measured across an available panel of 71 kinases involved in multiple signaling pathways critical for proliferation and cell homeostasis. In order to directly measure the inhibition spectrum of T3 on CLK kinase activities in comparison with KH-CB19, a LANCE® Ultra kinase enzymatic assay was utilized. While percent inhibition of activity for each kinase was measured with two concentrations of T3 (100 nM and 1000 nM) in duplicate data points in an ATP competitive assay at 100 µM, CLKs, DRYKs and SRPKs kinase activity was measured in a 10 point assay with two of the reference points reported (1110 and 123 nM). No additional kinases beyond the CMGC family were inhibited from this panel (Supplementary Data 1), further confirming the selectivity of the T3 inhibitor (Fig. 3a).

Several features of T3 likely contribute to its CLK inhibitory properties. The hinge binder of T3 is aminoimidazo[1,2-a]pyridine moiety to allow for an improved binding to the catalytic subunit of the CLK proteins (Supplementary Fig. 2). Additionally, the intermolecular hydrogen bond between the main-chain carbonyl of Leu246 and amide NH allows for abundant protein interaction. Protein-inhibitor interaction is also further stablilized by the bond between Lys193 and the nitrogen atom of the terminal pyridine. The solvent exposed piperazine moiety and hydrophobic phenyl ring could potentially be the reason for the improved potency and selectivity (Fig. 3b) in a head to head comparison with KH-CB19 (the most potent previously described CLK small molecule inhibitor) under the same assay conditions.

Supplementary Note 2

The T3 compound reduces CLK phosphorylation activity. Therefore, we hypothesized that artificially reducing CLK expression may have similar effects on RNA splicing. To test this notion, we knocked down *CLK* expression by transfecting *CLK* siRNA into HCT116 cells and sequencing the resulting transcriptomes with RNA-Seq (Supplementary Table 4).

We compared each *CLK* knockdown library and the reagent-only library to the NT3 siRNA control, and produced a list of AS events found to be differentially spliced in any of the *CLK* siRNA libraries but not in the reagent-only library. We then compared this event list to lists of differentially spliced events from the T3-treated HCT116 datasets (Supplementary Fig. 11b).

In total, 1580 unique AS events were differentially spliced in any of the *CLK* knockdown libraries. Of these events, 875 (55%) were found in at least one of the two T3-treated HCT116 AS event lists, confirming that many of the effects of T3 treatment are due to loss of CLK phosphorylation activity as opposed to off-target effects. Almost half of the events resulting from *CLK* knockdown were not found to be differentially spliced in the T3 treated datasets, which can be partially explained by differences in biological response to depleting *CLK* RNA versus inhibiting CLK phosphorylation activity and possible off target effects of RNAi.

Genes differentially spliced in both T3 treated cells and cells transfected with *CLK* siRNA are likely to be specifically affected by loss of CLK activity. Biological processes likely to be affected by splicing changes in this common set of genes were identified by constructing a gene interaction network with the ReactomeFI Cytoscape plugin [7]. Functional enrichment analysis was then performed using the genes in the network (Table 13). Biological processes enriched among genes differentially spliced in both T3 treated and *CLK* siRNA transfected cells included "gene expression", "mitotic cell cycle", "chromatin modification", and "nuclear mRNA splicing, via spliceosome". CLK activity likely plays a crucial role in these biological processes in particular.

Supplementary Note 3

The RNA binding protein (RBP) class of SR proteins are the canonical effectors of CLK function in splicing. To assess the consequence of CLK inhibition by T3 in a cellular assay (MDA-MB-468, 3 hours exposure to T3), SR protein phosphorylation was measured by western blotting using anti-pan-phospho-SR antibody, which selectively recognizes phosphorylated variants of multiple classical members of the SR family. Treatment of non-stimulated HCT116 cells with T3 led to a dramatic reduction in the basal level of phosphorylated SRSF4 (SRp75), SRSF6 (SRp55), and SRSF1 (SF2) in a concentration–dependent manner (Supplementary Fig. 5). The reduced phosphorylation of SR proteins is clearly evident at subnanomolar concentrations with T3 treatment (log -7.4 M). In contrast and consistent with the differences in the IC_{50} values, KH–CB19 showed a lesser decrease in the basal phosphorylation level of these SR proteins evident only at the highest experimental concentration of (log -5.0 M = 10 nM). These data indicate a significantly greater inhibitory effect of T3 on CLK activity compared to KH–CB19 which is a different chemical scaffold, but also of lower potency.

Supplementary Note 4

Next, we examined the effectiveness of CLK inhibition by T3 on alternative splicing of a few target mRNAs in comparision with KH–CB19. Alternatively spliced transcripts of *S6K* have previously been shown to be expressed in response to the modulation of CLK activity [1]. Therefore, to assess the potency of T3 treatment, we quantified the abundance of the novel transcripts of *S6K* as well as three novel conjoined gene events identified in our RNA-seq data sets in a real time quantitative RT–PCR (qRT–PCR) assay. We designed primer sets spanning the identified event junctions and measured their expression level in HCT116 and 184-hTERT cell lines treated with increasing concentrations of both T3 and KH–CB19 (Supplementary Fig. 10). The expression level of the alternatively spliced isoform of *S6K* was only evident in T3 treated

cell lines while this transcript was undetectable with KH–CB19 treatment in both HCT116 and 184-hTERT lines. Additionally, the relative abundance of all three conjoined gene events examined in this assay were significantly higher in T3 treated cell lines. While the abundance of *MRPS10-GUCA1B* transcript was increased in response to both T3 and KH-CB19, indicating some affects with both inhibitors on the splicosome machinery, the other two conjoined gene transcript variant tested in this assay were only detected with T3 treatment in both HCT116 or 184-hTERT cell lines (Supplementary Fig. 10). This data further confirms T3 to be a more efficacious inhibitor with a more profound effect on alternative splicing machinery than KH–CB19.

Supplementary Note 5

We validated the detection of CG events with two orthogonal methods, first using genome-wide PacBio long read sequencing [8] and second by targeted PCR-sequencing of selected events.

The proportion of CG events with PacBio read support was 117/205 (57%) in HCT116 stranded libraries and 344/988 (35%) in HCT116 unstranded libraries. PacBio sequencing suffers from low throughput and lower sensitivity compared to RNA-Seq which may result in a large number of false negative validations. Therefore, a set of 52 conjoined gene events were selected from the short read RNA-seq libraries, for targeted sequencing (Supplementary methods) in HCT116 and 184-hTERT RNA. A total of 37 of 52 (71.2%) CG isoforms were validated by targeted sequencing (Supplementary Data 23). Interestingly, 5 apparently new CG isoforms were detected in the validation dataset. Upon inspection, 4 were found to be alternative isoforms of other CGs selected for validation (2 of which were previously detected in the RNA-Seq datasets); the other is similar to another validation input isoform except that it involves a paralog of the upstream gene. This CG isoform is likely due to reads misaligned to the paralog gene. Considering CG parent genes only, and ignoring specific splice sites, 40 (76.9%) of the CG events targeted for validation were confirmed as present.

Of the 52 CGs selected for validation, 40 were found only in the HCT116 RNA-Seq CG lists, 2 were found in only the 184-hTERT RNA-Seq CG list, and 10 were found in both cell types. All but one detected CG in the targeted sequencing dataset were found in both HCT116 and 184-hTERT cell types. One CG, which was not chosen for validation, was found in just HCT116 cells.

Supplementary Note 6

Since transcription and splicing are coupled, we also sought to determine how CLK inhibition affects overall transcription in the genome. To address this question we elucidated gene expression trends in an analgous manner to ΔAS events, by clustering FPKM ranked gene expression (Supplementary Information). This resulted in 6 clusters for the unstranded HCT116 RNA-Seq dataset, 5 clusters each for the stranded HCT116 RNA-Seq and 184-hTERT datasets (Supplementary Data 17, 18). All three datasets exhibit similar FPKM profile clusters (Supplementary Fig. 19a). The dominant effect is monotonic T3 induced gene downregulation, as seen in the largest cluster 1, and cluster 3 (Supplementary Fig. 19a, 72% and 11% of clustered genes) with a smaller proportion of genes up-regulated (clusters 2 and 4, 11% and 5% of clustered genes). A small number of genes exhibit non-monotonic responses (clusters 5 and 6) similar to a minority pattern observed for AS events, implying these genes may be affected by the secondary consequences of blocking exon recognition with T3.

To determine the biological processes represented in FPKM gene clusters, we performed functional enrichment analysis separately for each set of clustered genes (Supplementary Fig. 19b, Supplementary Data 19). For the HCT116 datasets, only analysis of clusters 1–3 resulted in a list of enriched biological processes; For the 184-hTERT dataset, only clusters 1–4 produced significantly enriched biological process terms ($FDR < 0.05$). In both HCT116 and 184-hTERT cells, treatment causes the down-regulation of RNA splicing and RNA processing genes. Additionally genes involved in cell cycle regulation and spindle assembly checkpoints, as well as DNA repair related genes (*e.g.* BRCA1) were significantly down regulated. Down-regulation of cell cycle regulators upon T3 treatment suggests that CLK inhibition may disrupt cell cycle regulation and

is consistent with the observation of cell cycle arrest and DNA damage response signals with T3 treatment. RNA splicing is inhibited during mitosis [9] and appears to involve the dephosphorylation of SRSF10 proteins [10]. In addition, down-regulation of SRSF3 induces G1 cell cycle arrest in HCT116 colon cancer cells [11]. Splicing repression via CLK inhibition may thus have a similar effect. Up-regulated genes were many fewer than down-regulated genes and thus affected fewer biological processes. Histone genes were found to be enriched among only statistically significant up-regulated gene expression clusters in all three datasets.

Supplementary Note 7

CGs apparently occurring exclusively in 184-hTERT cells may be partially explained by cell-type specific gene expression profiles (*e.g.* low gene expression in HCT116 vs. 184-hTERT at a given locus). To investigate this possibility, we calculated FPKM values for genes involved in 184-hTERT-specific CGs for each of the HCT116 and 184-hTERT datasets. The FPKM distributions of 184-hTERT-specific CG partner genes reveal a pattern of higher expression in 184-hTERT samples (Supplementary Fig. 27). Therefore, the presence of a large number of 184-hTERT-specific CGs may be at least partially explained by reduced expression of participating genes in HCT116 cells.

Supplementary Note 8

NMD (non-sense mediated mRNA decay) is an inherent cytoplasmic cellular surveillance mechanism triggered in response to the formation of transcripts with premature translation termination codons (PTC) [12]. To evaluate the effect of T3 on NMD we transfected a previously described [13] NMD reporter plasmid in HeLa cells 48 hours before T3 treatment. Inhibition of CLK activity by T3 exhibited no effect on NMD pathway based on the luciferase reporter assay. There was only a minimal increase in luciferase activity observed at the 50.0 µM concentration (Supplementary Fig. 8). Inhibition of NMD regultors such as *UPF1* and *SMG1* with siRNA is reported as a positive control for this NMD assay.

Supplementary References

[1] Araki, S. *et al.* Inhibitors of CLK protein kinases suppress cell growth and induce apoptosis by modulating pre-mRNA splicing. *PLOS ONE* **10**, 10.1371/journal.pone.0116929 (2015).

[2] Langfelder, P. & Horvath, S. WGCNA: an R package for weighted correlation network analysis. *BMC Bioinf.* **9**, 10.1186/1471–2105–9–559 (2008).

[3] Mi, H., Poudel, S., Muruganujan, A., Casagrande, J. T. & Thomas, P. D. PANTHER version 10: expanded protein families and functions, and analysis tools. *Nucleic Acids Res.* **44**, D336–D342 (2015).

[4] Fedorov, O. *et al.* Specific CLK inhibitors from a novel chemotype for regulation of alternative splicing. *Chem. Biol.* **18**, 67–76 (2011).

[5] Muraki, M. *et al.* Manipulation of alternative splicing by a newly developed inhibitor of Clks. *J. Biol. Chem.* **279**, 24246–24254 (2004).

[6] Mott, B. T. *et al.* Evaluation of substituted 6-arylquinazolin-4-amines as potent and selective inhibitors of cdc2-like kinases (Clk). *Bioorg. Med. Chem. Lett.* **19**, 6700–6705 (2009).

[7] Wu, G., Feng, X. & Stein, L. A human functional protein interaction network and its application to cancer data analysis. *Genome Biol.* **11**, 10.1186/gb–2010–11–5–r53 (2010).

[8] Eid, J. *et al.* Real-time DNA sequencing from single polymerase molecules. *Science* **323**, 133–138 (2009).

[9] Blencowe, B. J. Splicing regulation: the cell cycle connection. *Curr. Biol.* **13**, R149–R151 (2003).

[10] Shin, C. & Manley, J. L. The SR protein SRp38 represses splicing in M phase cells. *Cell* **111**, 407–417 (2002).

[11] Kurokawa, K. *et al.* Downregulation of serine/arginine-rich splicing factor 3 induces G1 cell cycle arrest and apoptosis in colon cancer cells. *Oncogene* **33**, 1407–1417 (2013).

[12] Behm-Ansmant, I. *et al.* mRNA quality control: an ancient machinery recognizes and degrades mRNAs with nonsense codons. *FEBS Lett.* **581**, 2845–2853 (2007).

[13] Yamashita, A. *et al.* SMG-8 and SMG-9, two novel subunits of the SMG-1 complex, regulate remodeling of the mRNA surveillance complex during nonsense-mediated mRNA decay. *Genes Dev.* **23**, 1091–1105 (2009).

[14] Ray, D. *et al.* A compendium of RNA-binding motifs for decoding gene regulation. *Nature* **499**, 172–177 (2013).

[15] Gerstberger, S., Hafner, M. & Tuschl, T. A census of human RNA-binding proteins. *Nat. Rev. Genet.* 829–845 (2014).

[16] The UniProt Consortium. UniProt: a hub for protein information. *Nucleic Acids Res.* **43**, D204–D212 (2014).

[17] Stark, C. *et al.* BioGRID: a general repository for interaction datasets. *Nucleic Acids Res.* **34**, D535–D539 (2006).

[18] Orchard, S. *et al.* The MIntAct project–IntAct as a common curation platform for 11 molecular interaction databases. *Nucleic Acids Res.* **42**, D358–D363 (2014).
