# Supplementary material for: CLK-dependent exon recognition and conjoined gene formation revealed with a novel small molecule inhibitor
Source: Nat Commun. 2017 Feb 23;8:7. doi: 10.1038/s41467-016-0008-7 (PMC5431906; doi:10.1038/s41467-016-0008-7)
Supplement: Supplementary file 40 — Reviewer reports and authors' response from the peer review of this Article at Nature Communications. [file 41467_2016_8_MOESM40_ESM.pdf]

Reviewers' Comments:

Reviewer #1 (Remarks to the Author)

In this work the authors identify a novel inhibitor of the splicing factor CLK kinases named T3 by high-throughput screening of synthetic compounds. Through extensive and detailed pharmacological assays, they thoroughly test the functionality of this inhibitor and demonstrate that T3 displays lower cytotoxicity and higher specificity than previous CLK inhibitors.
To investigate the genome-wide impact of inhibition of CLK activity on transcription and splicing regulation, the authors carried out RNA-seq experiments using non-transformed immortalized cells and cancer cells treated with different doses of T3. These experiments revealed a widespread impact of CLK activity on alternative splicing regulation, with alternative exon cassette being the most regulated events. Moreover a significant overlap was found between T3-regulated splicing events and those regulated by CLK silencing, thus confirming the specificity of this drug.
Interestingly, analysis of the RNA-seq datasets also revealed that T3-mediated inhibition of CLKs induces the production of several conjoined gene transcripts. Consensus motifs for different RNA-binding proteins (RBPs) were identified within these transcripts and notably several of these RBPs are found to interact with CLK2 by mass-spec analysis. These results suggest a previously unreported role for CLK kinases in transcript termination, whose inhibition can lead to aberrant expression of conjoined transcripts from flanking genes.
Although the amount of data produced and described in this manuscript is impressive and of high quality, the overall message is somewhat lost due to excessive technical details. The amount of supplementary tables and figures is really overwhelming for the reader, and the manuscript appears more as a report of what was done rather than a report of a specific discovery.
Description of the Figures is also sometimes confusing. For instance, different panels of Figure 3 are described in distant sections of the Results, and it is unclear why these results are shown in the same figure if the authors decided to describe them independently. A more consistent description of figures in the text would improve the clarity of the manuscript. Regarding the analysis of gene expression, splicing and conjoined transcripts, the authors remain very descriptive and do not attempt to identify mechanistic features. As an example, they do not search for the enrichment of consensus motifs for splicing factors regulated by CLKs in the exons/introns affected by T3. Similarly, although they search for motifs in the conjoined regions, they do not assess whether the RNA binding proteins potentially binding to these motifs are regulated by CLKs and/or are actually involved in the defect observed after treatment with T3. They also report detailed analysis of a number of clusters of splicing events, but the biological relevance of such clusters is not addressed. Lastly, the discussion appears as the summary of the description of the results, without providing additional insight to the reader. Overall, the suggestion is to limit the manuscript to one specific message and to go more in detail into its description. In particular, the role of CLKs in transcript termination and the induction of conjoined transcripts upon their inhibition appear the most novel and appealing findings. The detailed description of the compound identification and its biochemical characterization could be reported in a separate paper for a more specialized journal.

Reviewer #2 (Remarks to the Author)

This paper reports a new inhibitor of CLK family kinases, T3, with greater potency and selectivity compared to previous inhibitors. The paper is well written and the characterization of T3 as a chemical tools is well documented. The authors go on to use T3 to characterize the effects of inhibiting CLK catalytic activity on alterative splicing and discovered a role for CLKi in promoting formation of conjoined genes. While the later is due to inhibition of CLK phosphorylation activity, the authors also demonstrate that knock down of many members of a network of CLK2 associated proteins can have similar results. These results and the T3 tool compound are important contributions to understanding regulation of RNA splicing. I just have a few minor comments and suggestions.

• Page 5: "...mirrored in cell-based in vitro conditions in a long term (>24 hours) cytotoxicity assay, when compared with KH-CB19 (Fig. 1b, Supplemental Fig. S5b)."
Fig 1b is does not show a cytotox assay.
• Page 9: "In contrast with the above, genes involved in toll-like receptor signaling were statistically over-represented only in the higher concentration 1.0-10.0 μM contrasts, suggesting the latter to be a secondary consequence of CLK inhibition." Might off target effects of inhibitors be a more reasonable explanation?
• Page 18: "Thus a major initial consequence of CLK inhibition is likely reduced expression of many RNA-processing factors." Can the authors support this statement with analysis from their RNA-seq data? Does T3 affect the protein levels of of CLKs?
• Will the authors make T3 available to the community to use in the future?

Reviewer #3 (Remarks to the Author)

Transcriptome dynamics of CLK dependent exon recognition and conjoined gene formation revealed with a novel small molecule inhibitor.

Simplified approach and findings
The authors developed a small molecule inhibitor of the CLK family –a protein family involved in gene splicing – that was many times more potent than previous inhibitors. They used their inhibitor to inactivate this protein family for six hours at different drug concentrations in two cell types. Then they used two different RNA sequencing technologies and a total of three sequencing approaches to identify splicing differences that result from this inactivation. Most of the splicing changes they found became more extreme at higher doses of the inhibitor, and most of the changes found in the smaller sequencing set were found in the larger sequencing set. About half of the identified alternative splicing events were skipped exon events. Inhibition of the protein family also resulted in conjoined genes. The authors identified clusters and motifs associated with splicing changes. They independently knocked down downstream genes and also observed increased conjoined gene formation. They also observed a preponderance of splicing factors among earliest responders to CLK inhibition, which they interpreted as autoregulation.

Strengths
-S1. Developed a much more potent inhibitor of CLK.

-S2. Good use of causal reason (looking for monotonic changes, earliest changes, independent knockdown of downstream genes)

-S3. Good use of biological and technical replicates to show reproducibility

-S4. Generated a wealth of data potentially useful for follow-up

-S5. Offered some insight into mechanism of conjoined gene formation when they knocked down associated factors

-S6. Identification of autoregulation was a meaningful interpretation of their generated data set

Weaknesses
-W1. Incomplete story about role of CLK inhibition in human health and disease

The authors show that if CLK is inhibited, then various splicing events follow. This observation is most relevant if two other conditions are met: 1) That there are some health and disease states that correspond to lesser or greater CLK activity; and 2) The consequent splicing events in turn meaningfully impact human health and disease. It would be appropriate for these two conditions to be instead firmly established in a later paper, but clues at least pointing in the affirmative on these conditions should be more explicitly stated in the paper.

-W2. Usefulness of new drug incompletely argued for

Although the potency of their new inhibitor is impressive, to show that their new inhibitor is also helpful, they should explain the limitations of the previous inhibitor (KH-CB19) – for example, they should address the questions as to whether sufficient knockdown of CLK could be achieved by compensating for KH-CB19's lower potency through use of a higher concentration.

-W3. The conspicuous absence of a particular biological replicate

Although the authors do a strong job in general with biological and technical replicates, I was surprised that they did not attempt to replicate their findings in hematopoietic cells, given this lineage's involvement in the myelodysplastic syndrome and chronic lymphocytic leukemia mentioned in the paper's introduction as diseases in which aberrant splicing is common. Nonetheless, restarting the experiment with hematopoietic cells would cause an inappropriate delay at this juncture.

Verdict: *Accept, with revisions to the manuscript but without new experiments.*

Responses to the reviewers.

We thank all three reviewers for comments which we have addressed with the revised manuscript, details below:

Reviewer #1 (Remarks to the Author):

In this work the authors identify a novel inhibitor of the splicing factor CLK kinases named T3 by high-throughput screening of synthetic compounds. Through extensive and detailed pharmacological assays, they thoroughly test the functionality of this inhibitor and demonstrate that T3 displays lower cytotoxicity and higher specificity than previous CLK inhibitors.

To investigate the genome-wide impact of inhibition of CLK activity on transcription and splicing regulation, the authors carried out RNA-seq experiments using non-transformed immortalized cells and cancer cells treated with different doses of T3. These experiments revealed a widespread impact of CLK activity on alternative splicing regulation, with alternative exon cassette being the most regulated events. Moreover a significant overlap was found between T3-regulated splicing events and those regulated by CLK silencing, thus confirming the specificity of this drug.
Interestingly, analysis of the RNA-seq datasets also revealed that T3-mediated inhibition of CLKs induces the production of several conjoined gene transcripts. Consensus motifs for different RNA-binding proteins (RBPs) were identified within these transcripts and notably several of these RBPs are found to interact with CLK2 by mass-spec analysis. These results suggest a previously unreported role for CLK kinases in transcript termination, whose inhibition can lead to aberrant expression of conjoined transcripts from flanking genes.

Although the amount of data produced and described in this manuscript is impressive and of high quality, the overall message is somewhat lost due to excessive technical details. The amount of supplementary tables and figures is really overwhelming for the reader, and the manuscript appears more as a report of what was done rather than a report of a specific discovery. Description of the Figures is also sometimes confusing. For instance, different panels of Figure 3 are described in distant sections of the Results, and it is unclear why these results are shown in the same figure if the authors decided to describe them independently. A more consistent description of figures in the text would improve the clarity of the manuscript.

RESPONSE: We have taken these comments on board and shortened the manuscript by almost 2000 words, relegating some technical material to the supplement and focusing the text on the main findings. We have also shortened the supplement, by removing a section on the technical comparison of RNA-seq libraries and by combining figures and shortening the methods descriptions with more reference to published literature where appropriate. We have also reordered the text to make references to Fig3 less jumpy in the text, we have also split figure three so that the heatmap showing motif enrichment now comes as a separate figure, to reduce the off-sequence figure referencing.

Regarding the analysis of gene expression, splicing and conjoined transcripts, the authors

remain very descriptive and do not attempt to identify mechanistic features. As an example, they do not search for the enrichment of consensus motifs for splicing factors regulated by CLKs in the exons/introns affected by T3.

RESPONSE: In fact this was done, but we realize it was not clearly explained. The RNA binding factor motif enrichment, as shown in Fig 2c and the related supplemental figures, show the pattern of binding motifs that are relatively enriched or depleted based on an unbiased statistical background calculation for all binding factors, as described in the supplement. The list of binding factors seen as enriched above background, does include many CLK associated SR binding factors, which we mentioned in the text, but did not highlight clearly in the main figure. We have modified the Figure 2c and legend to put a red dot by the binding factor motifs that are CLK associated RNA binding factors and as can be seen, this draws out that many of the motifs exhibiting statistically significant enrichment from an unbiased search, are indeed CLK associated.

Similarly, although they search for motifs in the conjoined regions, they do not assess whether the RNA binding proteins potentially binding to these motifs are regulated by CLKs and/or are actually involved in the defect observed after treatment with T3.

RESPONSE: This is shown in (old Fig 5), figure 6 and associated supplemental figures. We first showed that the cell lines being used contain known (as well as some new) SR binding factors in association with CLK complexes, by IP-MS. These contain for example, canonical SR proteins that are known targets of CLK (referenced), as well as CLK complex associated proteins. We then systematically knocked down, with siRNA, the factors whose motifs appeared as enriched or depleted in the conjoined gene exons and flanking sequences/associated with CLK complexes. We show that this results in an increase in conjoined gene transcripts, by two sequencing methods. The list of factors is also heavily enriched in 3' end processing factors associating with CLK, which provides a further mechanistic link. We have tried to make the text clearer to bring this point out.

They also report detailed analysis of a number of clusters of splicing events, but the biological relevance of such clusters is not addressed.

RESPONSE: The main reason for clustering the splicing events, was to establish the core set of responses that exhibit clearly monotonic responses to CLKi, so that motif analysis could be conducted. This is in fact the majority of events, grouping into two or three clusters in each set. The remaining clusters are small and exhibit variable, non-monotonic responses that likely represent measurement noise, or secondary biological effects. The strong event type distributions between the largest clusters (reciprocal distributions of SE and RI events for example) shows that the biological relevance of the largest clusters is related to the regulatory effects of CLK, also evidenced by the strong enrichment of binding factor motifs in the events associated with those clusters. We have tried to make the wording simpler to bring these points out.

Lastly, the discussion appears as the summary of the description of the results, without providing additional insight to the reader.

RESPONSE: We have shortened the discussion and tried to bring in some insights into the possible mechanism and consequences of CLK associated CG formation.

Overall, the suggestion is to limit the manuscript to one specific message and to go more in detail into its description. In particular, the role of CLKs in transcript termination and the

induction of conjoined transcripts upon their inhibition appear the most novel and appealing findings. The detailed description of the compound identification and its biochemical characterization could be reported in a separate paper for a more specialized journal.

RESPONSE: We have shortened the manuscript in the sections other than CG, as noted above and also shortened the supplement. The description of the chemical structure and synthesis route would be essential for anyone wishing to synthesize and repeat the experiments, as noted by other reviewers, and also a requirement of the journal, so we have left this in, but we have reduced the amount of technical description in the main text, so as not to distract the reader.

Reviewer 2:

This paper reports a new inhibitor of CLK family kinases, T3, with greater potency and selectivity compared to previous inhibitors. The paper is well written and the characterization of T3 as a chemical tools is well documented. The authors go on to use T3 to characterize the effects of inhibiting CLK catalytic activity on alterative splicing and discovered a role for CLKi in promoting formation of conjoined genes. While the later is due to inhibition of CLK phosphorylation activity, the authors also demonstrate that knock down of many members of a network of CLK2 associated proteins can have similar results. These results and the T3 tool compound are important contributions to understanding regulation of RNA splicing. I just have a few minor comments and suggestions.

• Page 5: "...mirrored in cell-based in vitro conditions in a long term (>24 hours) cytotoxicity assay, when compared with KH-CB19 (Fig. 1b, Supplemental Fig. S5b)."
Fig 1b is does not show a cytotox assay.

Response: this is an erroneous figure ref which has been fixed.

• Page 9: "In contrast with the above, genes involved in toll-like receptor signaling were statistically over-represented only in the higher concentration 1.0-10.0 μM contrasts, suggesting the latter to be a secondary consequence of CLK inhibition." Might off target effects of inhibitors be a more reasonable explanation?

Response: potentially off target effects, or secondary consequences of splicing, and we have added a note to that effect in the text.

• Page 18: "Thus a major initial consequence of CLK inhibition is likely reduced expression of many RNA-processing factors." Can the authors support this statement with analysis from their RNA-seq data? Does T3 affect the protein levels of of CLKs?

Response: (i) In figure S21 we show from the Cytoscape enrichment map of clustered transcripts (FPKM) that cluster 1, which represents down regulated transcripts, is enriched for splicing factors. The expression/transcript analysis is described in more detail in the supplemental information (ii) We have added a western blot to the supplemental figures which shows the effect of T3 on the CLK proteins. During the time exposure of the experiments conducted, there is either no change or a small increase in CLK proteins, whereas as shown already, phosphorylation of target SR proteins is decreased under the same conditions. This is consistent with autoregulation, which is a described

feature of splicing. We have added a note to the text to this effect.

• Will the authors make T3 available to the community to use in the future?

Response: We have a small supply of T3 that we can share with collaborators, however we have taken care to include full details of the synthesis route and physical-chemical characterization, precisely because we wish others to be able to make and use this molecule for their own studies. With the information in the supplement, any small molecule chemistry provider company can produce sufficient material for very reasonable costs, the route is not difficult and uses fairly standard intermediates. The molecular structure is that of a drug-like small molecule and these are typically straightforward to synthesize, as is the case here. In fact, our experience is that the commercial chemical reagent providers usually pick up new molecules from the literature, especially kinase inhibitors and make them available off the shelf.

Reviewer 3

Transcriptome dynamics of CLK dependent exon recognition and conjoined gene formation revealed with a novel small molecule inhibitor.
Simplified approach and findings

The authors developed a small molecule inhibitor of the CLK family –a protein family involved in gene splicing – that was many times more potent than previous inhibitors. They used their inhibitor to inactivate this protein family for six hours at different drug concentrations in two cell types. Then they used two different RNA sequencing technologies and a total of three sequencing approaches to identify splicing differences that result from this inactivation. Most of the splicing changes they found became more extreme at higher doses of the inhibitor, and most of the changes found in the smaller sequencing set were found in the larger sequencing set. About half of the identified alternative splicing events were skipped exon events. Inhibition of the protein family also resulted in conjoined genes. The authors identified clusters and motifs associated with splicing changes. They independently knocked down downstream genes and also observed increased conjoined gene formation. They also observed a preponderance of splicing factors among earliest responders to CLK inhibition, which they interpreted as
autoregulation.

Strengths
-S1. Developed a much more potent inhibitor of CLK.
-S2. Good use of causal reason (looking for monotonic changes, earliest changes, independent knockdown of downstream genes)
-S3. Good use of biological and technical replicates to show reproducibility
-S4. Generated a wealth of data potentially useful for follow-up
-S5. Offered some insight into mechanism of conjoined gene formation when they knocked down associated factors
-S6. Identification of autoregulation was a meaningful interpretation of their generated data set

Weaknesses
-W1. Incomplete story about role of CLK inhibition in human health and disease
The authors show that if CLK is inhibited, then various splicing events follow. This observation is most relevant if two other conditions are met: 1) That there are some health and disease states that correspond to lesser or greater CLK activity; and 2) The consequent splicing events in turn

meaningfully impact human health and disease. It would be appropriate for these two conditions to be instead firmly established in a later paper, but clues at least pointing in the affirmative on these conditions should be more explicitly stated in the paper.

Response:

Indeed there have been previous reports (Yoshia et al, 2015 & Dominguez et al, eLife, 2016) showing that:

1. CLK2 is amplified and overexpressed in a subset of breast cancers. CLK1 has higher expression (which appears to be prognostic) and CLK1-regulated alternative splicing is deregulated in kidney cancers.

2. KD of CLK2 inhibits growth of the breast tumours in cell and animal models of the disease, correlating with splicing alterations. KD of CLK1 by TG003 or KH-CB19 blocks proliferation (Dominguez et al, eLife, 2016) similar to T3 treatment suggesting that T3 may be relevant in treatment of these diseases where CLKs are overexpressed.

Reference to these is made in the paper.

-W2. Usefulness of new drug incompletely argued for
Although the potency of their new inhibitor is impressive, to show that their new inhibitor is also helpful, they should explain the limitations of the previous inhibitor (KH-CB19) – for example, they should address the questions as to whether sufficient knockdown of CLK could be achieved by compensating for KH-CB19's lower potency through use of a higher concentration.

Response: we've highlighted this in the introductory text and throughout, to draw out the limitations of previous molecules. We've also mentioned this in the discussion, pointing out that although some of the effects can be seen with a weaker inhibitor, KHCB19, they are much lower in magnitude and we suspect that is why they have not been described before. Using higher concentrations of a weaker inhibitor tends to promote off-target effects at the expense of on-target, which is why potency is so important.

Our genome wide analyses were critically dependent on the high potency of T3 vs KH-CB19 to generate enough data points for the analyses. To illustrate the point, here are the relevant comparisons from Table S2.

Cell Line	Method	Drug	Conc	AS events	CG events
HCT116	Stranded-RNA-seq	T3	1 µM	3909	104
HCT116	Stranded-RNA-seq	KH-CB19	10 µM	284	19

-W3. The conspicuous absence of a particular biological replicate
Although the authors do a strong job in general with biological and technical replicates, I was surprised that they did not attempt to replicate their findings in hematopoietic cells, given this lineage's involvement in the myelodysplastic syndrome and chronic lymphocytic leukemia mentioned in the paper's introduction as diseases in which aberrant splicing is common. Nonetheless, restarting the experiment with hematopoietic cells would cause an inappropriate delay at this juncture.

Response: we thank the reviewer for this suggestion, which as he/she indicates, would work best in a future manuscript. The MDS splicing discoveries have been linked principally to SF3B proteins, which have a different function (branch point recognition) in splicing than CLK (exon recognition). Longer reference to this was included in the introduction for completeness and in the shortened paper we have reduced this to avoid confusing readers. We do however highlight the recent findings suggesting CLK2 is an oncogene in breast cancer, as noted above, and also the role of CLK splicing functions in cell cycle control.

Verdict: Accept, with revisions to the manuscript but without new experiments.

Reviewers' Comments:

Reviewer #1 (Remarks to the Author)

The authors have provided answers to the comments and criticisms raised to the first version of the manuscript. The effort made in shortening and focusing the work presented has improved the manuscript. It remains an immense source of data, often excessive and not sufficiently described, which certainly represents an optimal resource but not an easy article to read and to convey a message.

Reviewer #2 (Remarks to the Author)

this manuscript is now acceptable for publication.

Reviewer #3 (Remarks to the Author)

The authors responded satisfactorily to each of my concerns.

1) In the rebuttal, the authors highlight references included in this manuscript that argue for the clinical relevance of CLK expression levels and CLK-mediated alternative splicing events

2) I agree with the authors that the excerpt from Table S2 demonstrates the usefulness of the greater potency of their compound

3) The revised introduction appropriately no longer creates the unmet expectations for a hematological replicate
